# Author Correction: SMARCA4/2 loss inhibits chemotherapy-induced apoptosis by restricting IP3R3-mediated Ca^2+^ flux to mitochondria

**DOI:** 10.1038/s41467-023-37144-1

**Published:** 2023-03-21

**Authors:** Yibo Xue, Jordan L. Morris, Kangning Yang, Zheng Fu, Xianbing Zhu, Fraser Johnson, Brian Meehan, Leora Witkowski, Amber Yasmeen, Tunde Golenar, Mackenzie Coatham, Geneviève Morin, Anie Monast, Virginie Pilon, Pierre Olivier Fiset, Sungmi Jung, Anne V. Gonzalez, Sophie Camilleri-Broet, Lili Fu, Lynne-Marie Postovit, Jonathan Spicer, Walter H. Gotlieb, Marie-Christine Guiot, Janusz Rak, Morag Park, William Lockwood, William D. Foulkes, Julien Prudent, Sidong Huang

**Affiliations:** 1 Department of Biochemistry, McGill University, Montreal, QC Canada; 2 Rosalind and Morris Goodman Cancer Research Centre, McGill University, Montreal, QC Canada; 3 Department of Human Genetics, McGill University, Montreal, QC Canada; 4 Division of Medical Genetics, McGill University Health Centre, and Cancer Research Program, Research Institute of the McGill University Health Centre, McGill University, Montreal, QC Canada; 5 Medical Research Council Mitochondrial Biology Unit, University of Cambridge, Cambridge, UK; 6 Department of Integrative Oncology, British Columbia Cancer Agency, Vancouver, BC Canada; 7 Interdisciplinary Oncology Program, University of British Columbia, Vancouver, BC Canada; 8 Department of Pathology and Laboratory Medicine, University of British Columbia, Vancouver, BC Canada; 9 Department of Pediatrics, Research Institute of the McGill University Health Centre, Montreal Children’s Hospital, McGill University, Montreal, QC Canada; 10 Department of Specialized Medicine, Lady Davis Institute, Jewish General Hospital, McGill University, Montreal, QC Canada; 11 Division of Gynecologic Oncology, Segal Cancer Center, Jewish General Hospital, McGill University, Montreal, QC Canada; 12 Department of Oncology, Department of Obstetrics and Gynecology, University of Alberta, Edmonton, AB Canada; 13 Department of Pathology, McGill University Health Centre, Montreal, QC Canada; 14 Department of Medicine, Division of Respiratory Medicine, McGill University Health Centre, Montreal Chest Institute, Montreal, QC Canada; 15 Department of Biomedical and Molecular Sciences, Queen’s University, Kingston, ON Canada; 16 Department of Surgery, McGill University Health Center, Montreal, QC Canada; 17 Department of Pathology, Montreal Neurological Hospital/Institute, McGill University Health Centre, Montreal, QC Canada

**Keywords:** Cancer epigenetics, Cancer therapeutic resistance, Tumour-suppressor proteins, Apoptosis

Correction to: *Nature Communications* 10.1038/s41467-021-25260-9, published online 13 September 2021

The original version of this Article contained errors in the Supplementary Information and the Source Data. The actin Western blot in Supplementary Fig. 4c inadvertently duplicated the actin Western blot in Supplementary Fig. 4a. Supplementary Fig. 4c in the Supplementary Information has been updated with the correct Western blot. The Western blot in Supplementary Fig. 8b was performed incorrectly, providing an inaccurate pattern for SMARCA2. The experiment was repeated, and Supplementary Fig. 8b in the Supplementary Information was updated with the correct Western blot. The Source Data file was also updated with the correct Western blot.

