# Supplementary material for: SMARCA4/2 loss inhibits chemotherapy-induced apoptosis by restricting IP3R3-mediated Ca2+ flux to mitochondria
Source: Nat Commun. 2021 Sep 13;12:5404. doi: 10.1038/s41467-021-25260-9 (PMC8438089; doi:10.1038/s41467-021-25260-9)
Supplement: Supplementary file 1 — Supplementary Information [file 41467_2021_25260_MOESM1_ESM.pdf]

SUPPLEMENTARY INFORMATION

SMARCA4/2 loss inhibits chemotherapy-induced apoptosis by restricting IP3R3-mediated Ca^{2+} flux to mitochondria

Xue Y[1,2,3,4]*, Morris JL[5]*, Yang K[1,2]*, …, Prudent J[5§], Huang S[1,2§].

[1]Department of Biochemistry, McGill University, Montreal, Quebec H3G 1Y6, Canada.
[2]Goodman Cancer Research Centre, McGill University, Montreal, Quebec H3A 1A3, Canada.
[3]Department of Human Genetics, McGill University, Montreal, Quebec H3A 0C7, Canada.
[4]Department of Medical Genetics and Cancer Research Program, McGill University Health Centre, Montreal, Quebec H4A 3J1, Canada.
[5]Medical Research Council Mitochondrial Biology Unit, University of Cambridge, Cambridge Biomedical Campus, Cambridge CB2 0XY, UK.

* These authors contributed equally
§ These authors jointly supervised this work

Supplementary Figures 1-18

Supplementary References

Supplementary Tables 1-3

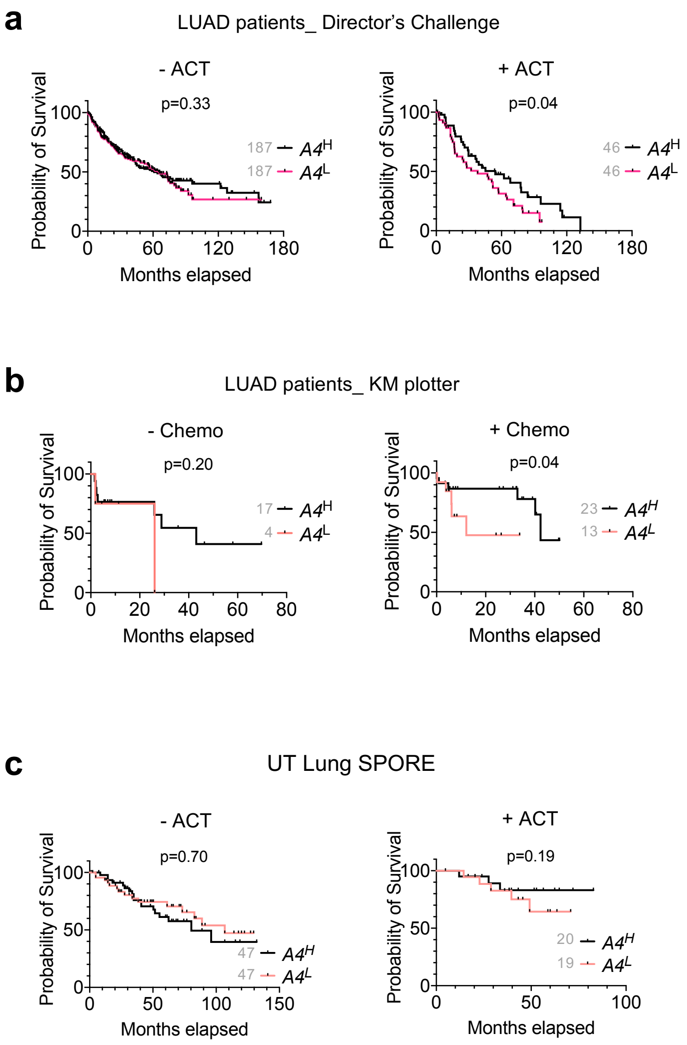

Supplementary Fig. 1 Reduced *SMARCA4* expression is associated with chemoresistance in non-small cell lung cancer, related to Fig. 1.

a Kaplan–Meier (KM) curves of overall survival in lung adenocarcinoma (LUAD) patients ± adjuvant chemotherapy (ACT). Director's Challenge Consortium for the Molecular Classification of Lung Adenocarcinoma [1] was analyzed and patients were stratified based on median of *SMARCA4* mRNA expression (jetset probe, Affy ID 213720_s_at). **b** KM curves of overall survival in lung adenocarcinoma patients ± chemotherapy. Kaplan–Meier Plotter [2] was used and patients were stratified based on auto select best cut-off of *SMARCA4* mRNA expression (jetset probe, Affy ID 213720_s_at). **c** KM curves of overall survival in lung cancer patients ± adjuvant chemotherapy. The UT Lung SPORE dataset [3] was analyzed and patients were stratified based on median of *SMARCA4* mRNA expression (jetset probe, Affy ID 213720_s_at). One-tailed Mantel-Cox test, p: *p*-value.

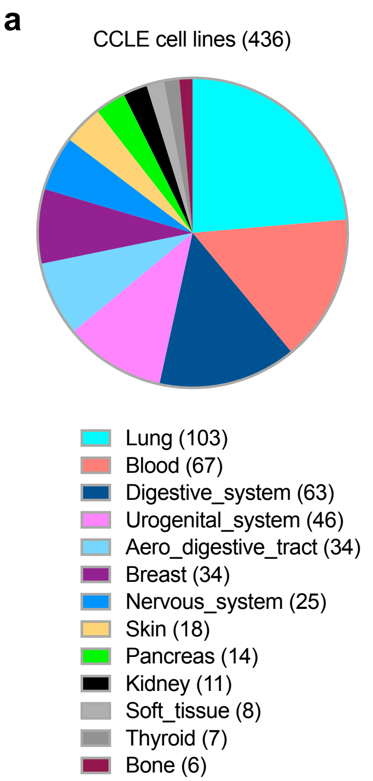

b

	Group	*SMARCA4* ranking	*SMARCA2* ranking
	*A4*H *A2*H	>70%	>70%
	*A4*H *A2*L	>70%	<30%
	*A4*L *A2*H	<30%	>70%
	*A4*L *A2*L	<30%	<30%

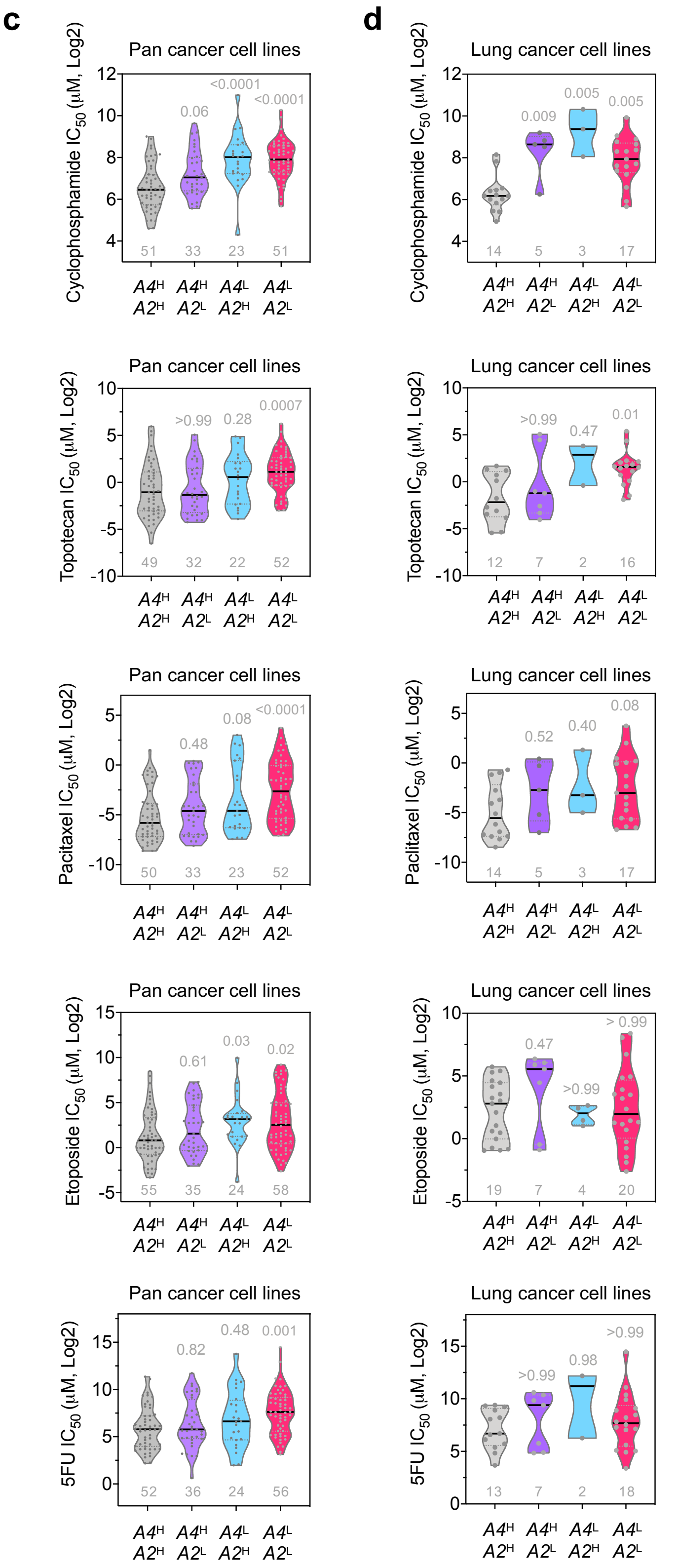

Supplementary Fig. 2 Reduced SMARCA4/2 expression is associated with resistance to chemotherapeutics in cancer cell lines, related to Fig. 1.

a Pie chart depicting the tissue of origins of cell lines with both mRNA expression and IC_{50} data available. Numbers of cell lines are indicated in parentheses for each tissue type. **b** Stratification of cell lines according to the mRNA expression of *SMARCA4/2*. $A4^{H}$: $SMARCA4^{High}$; $A4^{L}$: $SMARCA4^{Low}$; $A2^{H}$: $SMARCA2^{High}$; $A2^{L}$: $SMARCA2^{Low}$. **c, d** IC_{50} of indicated chemotherapy drugs in pan cancer cell lines (**c**) and lung cancer cell lines (**d**) with different mRNA expression levels for *SMARCA4* and *SMARCA2*. Cell line numbers are indicated in grey below each group. One-way ANOVA Kruskal–Wallis test followed by Dunn's test for multiple comparisons to $A4^{H}A2^{H}$ group. *p*-values are indicated in grey above each group.

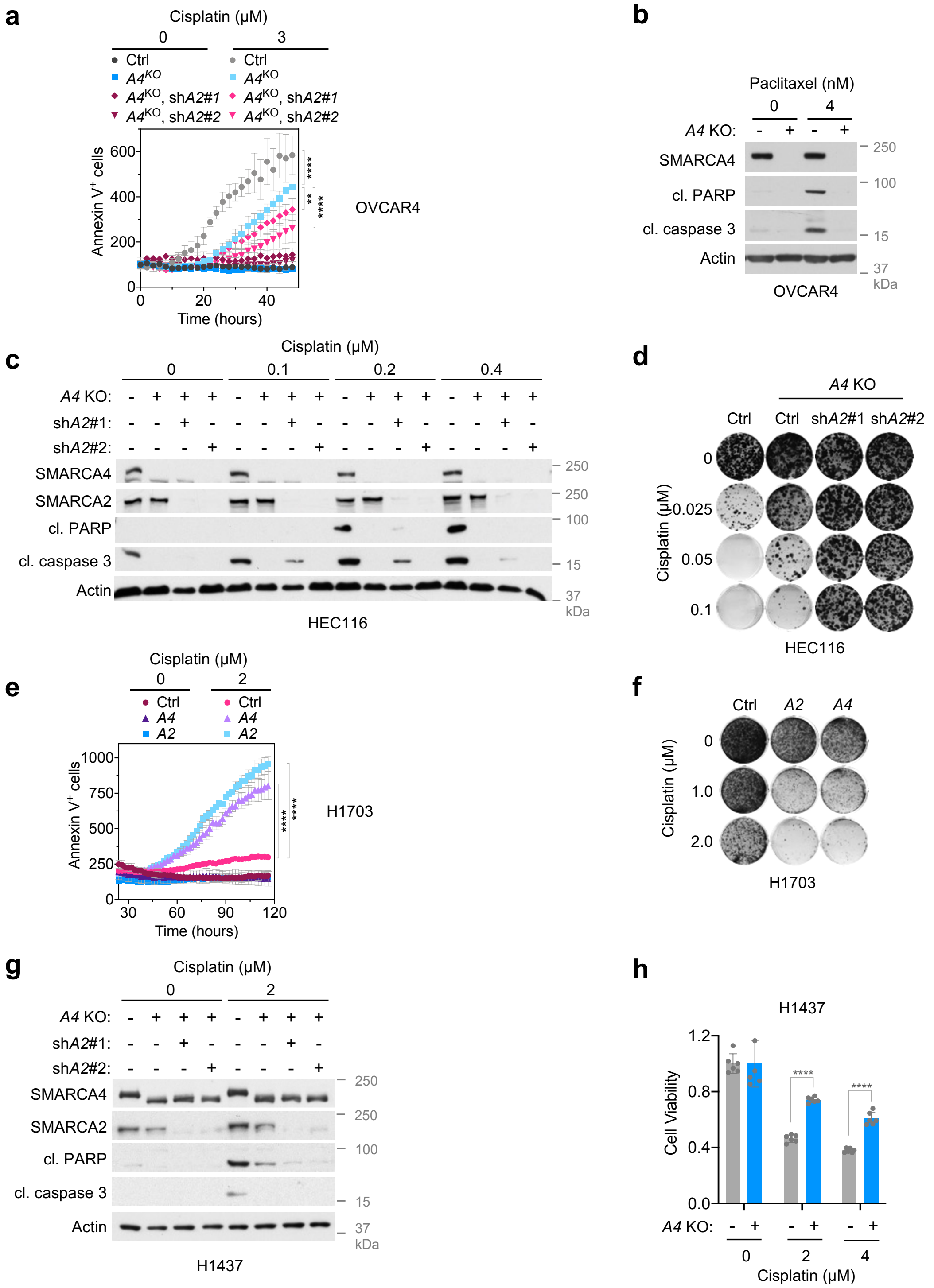

Supplementary Fig. 3 SMARCA4/2 loss causes resistance to chemotherapy drug in ovarian and lung cancers, related to Fig. 1.

a Annexin V staining of OVCAR4 cells with the indicated *SMARCA4* (*A4*) perturbation and cisplatin treatments. **b** Immunoblot analysis of OVCAR4 cells with the indicated *A4* perturbation and paclitaxel treatments (48 hours). **c, d** Immunoblot analysis (**c**, 48 hours) and colony formation (**d**, 12 days) of HEC116 cells with indicated *SMARCA4/2* (*4/2*) perturbations and cisplatin treatments. **e** Annexin V staining of H1703 cells with the indicated *A4* perturbation and cisplatin treatments. **f** Colony formation of H1703 cells with the indicated *A4/2* perturbations and cisplatin treatments (12 days). **g, h** Immunoblot analysis (**g**, 48 hours) and cell viability assay (**h**, 4 days) of H1437 cells with indicated *A4/2* perturbations and cisplatin treatments. Ctrl: Control; $A4^{KO}$: *SMARCA4* knockout; sh*A2*: shRNA targeting *SMARCA2*; cl.: cleaved. **a**, **e**, mean ± SD, n = 3 independent experiments, two-way ANOVA; **h**, mean ± SD, n = 6 independent experiments, two-tailed *t*-test; *p*-values (p), cisplatin treated: (**a**) Ctrl vs $A4^{KO}$ < 0.0001, $A4^{KO}$_shA2#1 vs $A4^{KO}$ – 0.0010, $A4^{KO}$_shA2#2 vs $A4^{KO}$ <0.0001; (**e, h**) all <0.0001. **p < 0.01, ****p < 0.0001.

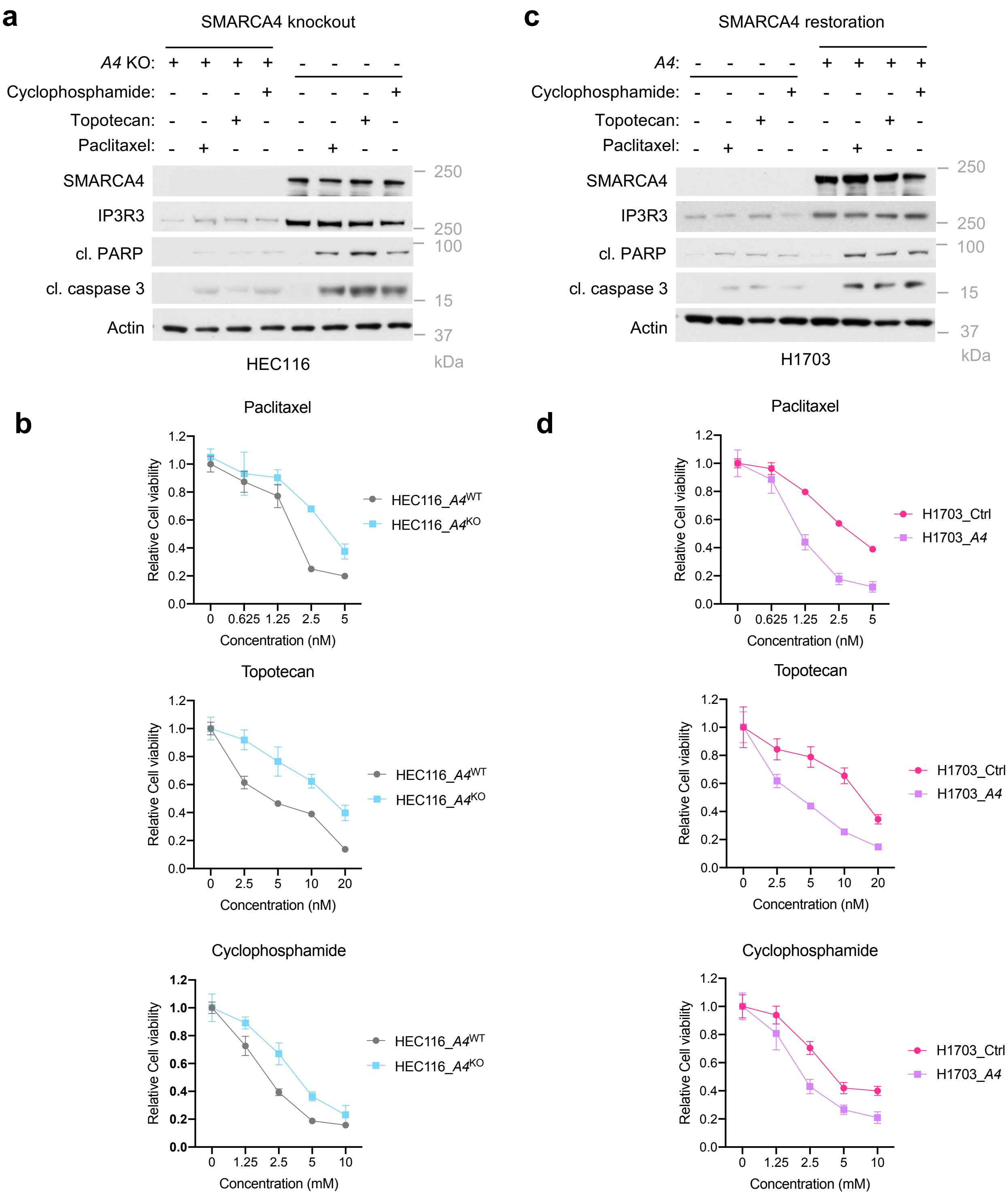

Supplementary Fig. 4 SMARCA4/2 loss causes resistance to cyclophosphamide, topotecan and paclitaxel in ovarian and lung cancer cells, related to Fig. 1.

a-d Immunoblot analysis (**a, c**) and cell viability assay (**b, d**) of HEC116 (**a, b**) and H1703 (**c, d**) cells with indicated *SMARCA4/2* perturbations and treatments of cyclophosphamide, topotecan and paclitaxel. **a**, **c**, cells were collected 48 hours after the treatment of 2 mM cyclophosphamide, 4 nM topotecan and 2 nM paclitaxel. $A4^{KO}$: *SMARCA4* knockout; *A4*: *SMARCA4*. **b**, **d**, mean ± SD, n = 6 independent experiments for all groups.

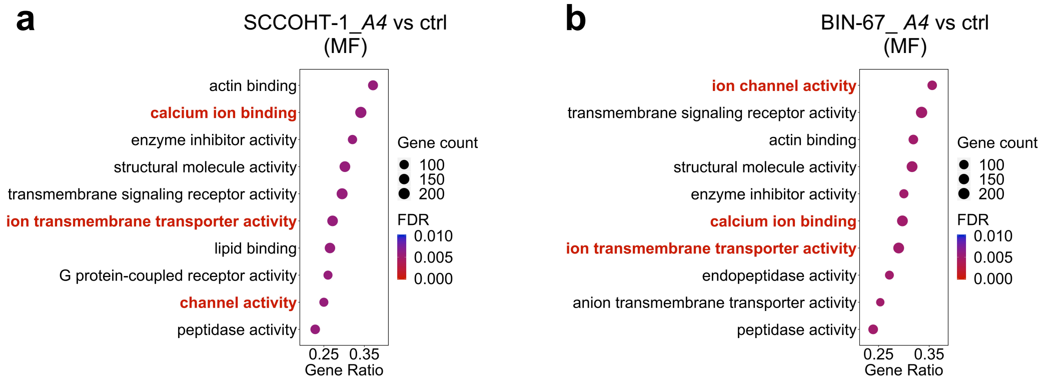

Supplementary Fig. 5 Gene Set Enrichment Analysis of published gene expression datasets in SCCOHT with SMARCA4 restoration, related to Fig. 2.

a, b Top 10 enriched gene ontology terms in SCCOHT-1 (**a**) and BIN-67 (**b**) cells with SMARCA4 restoration [4]. MF: gene sets derived from the GO Molecular Function Ontology. FDR: False Discovery Rate. Calcium/ion transportation terms are highlighted with red.

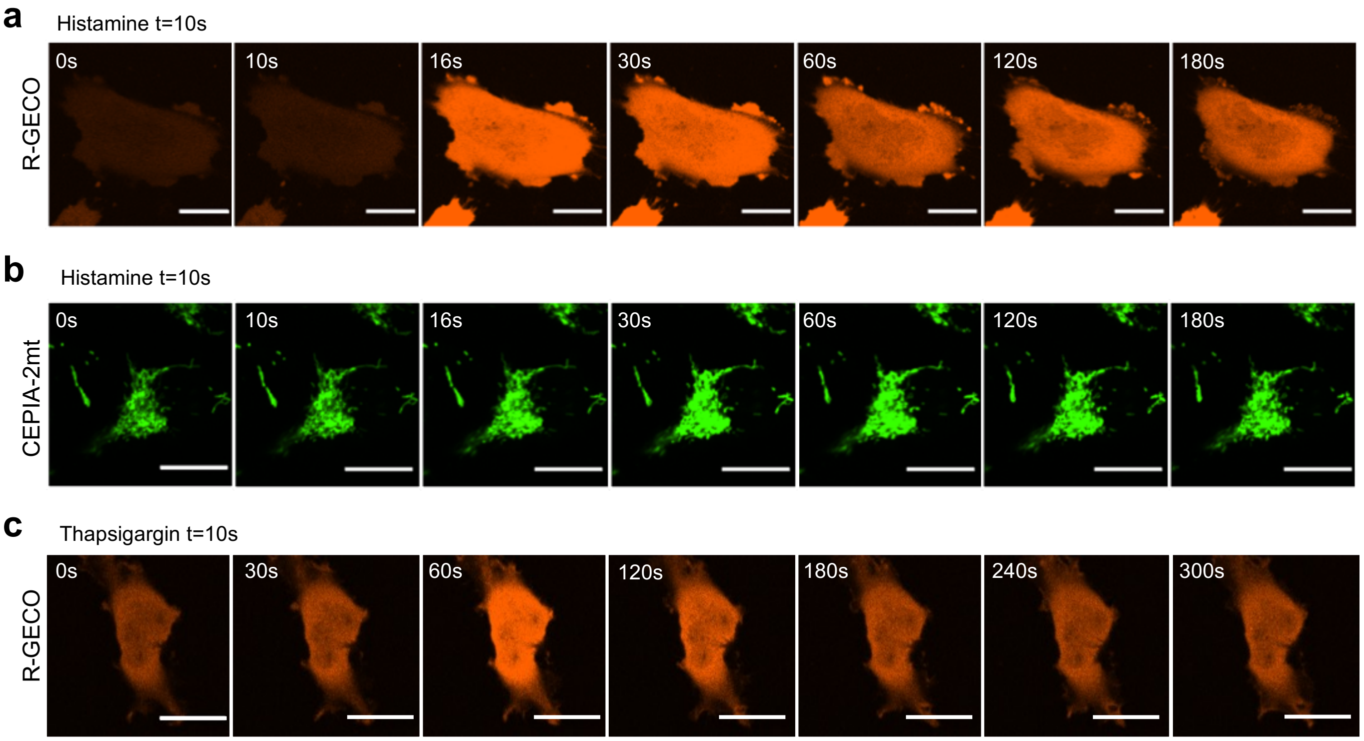

Supplementary Fig. 6 SMARCA4 modulates Ca^{2+} flux from ER to mitochondria, related to Fig. 2.
a Representative confocal time-lapse images of SMARCA4 expressing H1703 cells transfected with the cytosolic Ca^{2+} probe R-GECO. 100 µM histamine final was added at t = 10s. Scale bar, 25 µm. Corresponding to Fig. 2g. **b** Representative confocal time-lapse images of SMARCA4 expressing H1703 cells transfected with the mitochondrial Ca^{2+} probe CEPIA-2mt. 100 µM histamine final was added at t = 10s. Scale bar, 25 µm. Corresponding to Fig. 2h. **c** Representative confocal time-lapse images of SMARCA4 expressing H1703 cells transfected with the cytosolic Ca^{2+} probe R-GECO. 10 µM thapsigargin final was added at t = 10s. Scale bar, 25 µm. Corresponding to Supplementary Fig. 7b.

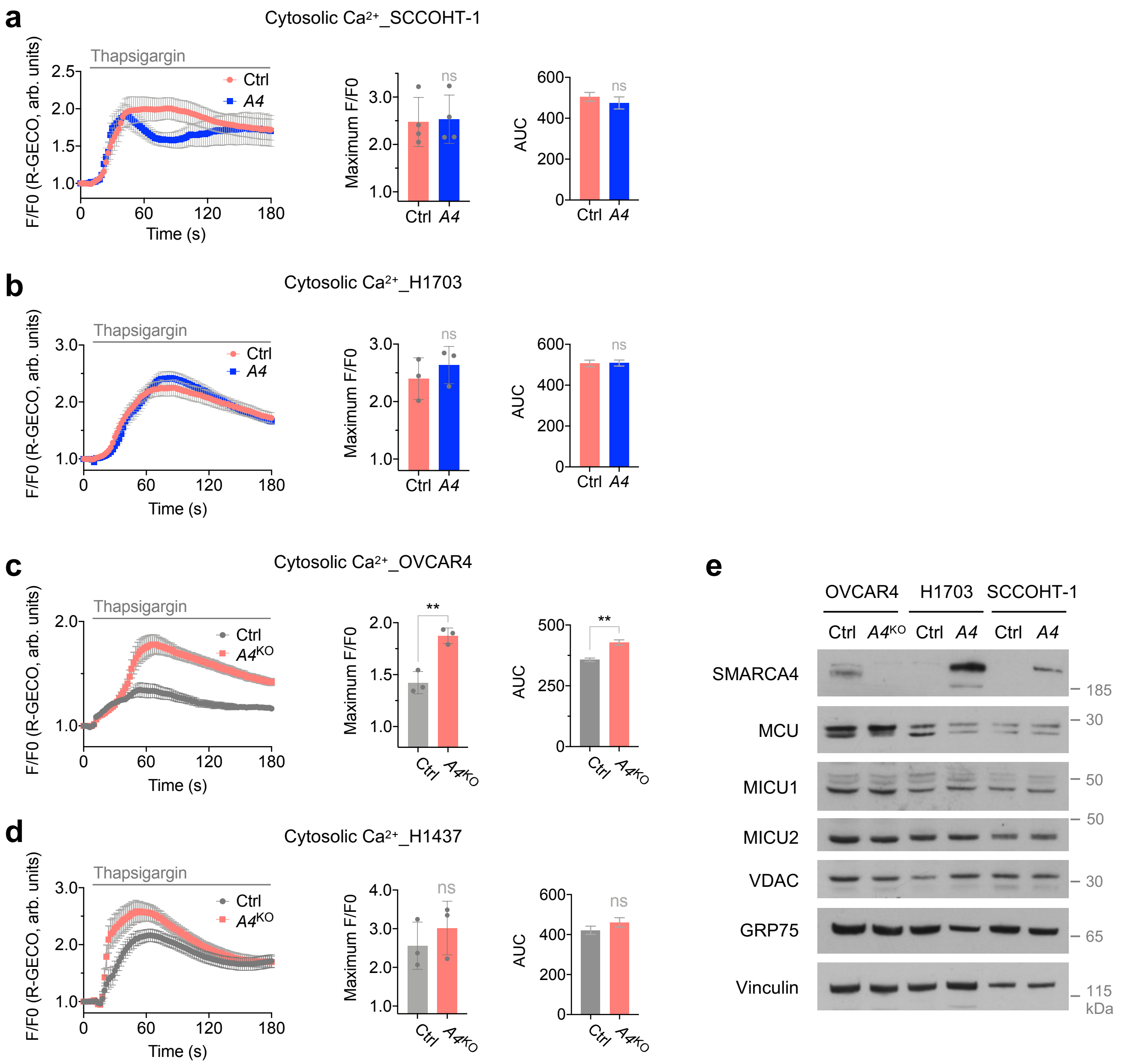

Supplementary Fig. 7 SMARCA4 loss does not reduce Ca^{2+} storage in the ER, related to Fig 2.

a Changes of cytosolic Ca^{2+} content in SCCOHT-1 cells ± SMARCA4 restoration upon thapsigargin stimulation. For all panels, 21 control (Ctrl) cells and 29 *SMARCA4* (*A4*)-expressing cells from n=4 independent experiments were analyzed. **b** Changes of cytosolic Ca^{2+} content in H1703 cells ± SMARCA4 restoration upon thapsigargin stimulation. For all panels, 31 Ctrl cells and 39 *A4* restored cells from n=3 independent experiments were analyzed. **c** Changes of cytosolic Ca^{2+} content in OVCAR4 cells ± *SMARCA4* knockout (*A4*^{KO}) upon thapsigargin stimulation. For all panels, 57 Ctrl cells and 59 *A4*^{KO} cells from n=3 independent experiments were analyzed. **d** Changes of cytosolic Ca^{2+} content in H1437 cells ± *SMARCA4* knockout (*A4*^{KO}) upon thapsigargin stimulation. For all panels, 46 Ctrl cells and 48 *A4*^{KO} cells from n=3 independent experiments were analyzed. **e** Immunoblots of the indicated proteins in OVCAR4, H1703 and SCCOHT-1 cells with the indicated *SMARCA4* perturbations. **a-d**, Left: traces of cytosolic Ca^{2+} content in indicated cell lines upon 10 µM thapsigargin stimulation (mean ± SEM). Middle: quantification of the maximal Ca^{2+} signal peaks induced by thapsigargin stimulation (mean ± SD). Right: quantification of the area under the curve (AUC) from (**a**) (mean ± SEM). The Ca^{2+} probe R-GECO (R-GECO F/F0) was used to monitor cytosolic Ca^{2+}. Arb. units: arbitrary units. Two-tailed unpaired t-test, *p*-values (p): (**a**) F/F0 – 0.8833, AUC – 0.1529; (**b**) F/F0 – 0.4453, AUC – 0.9043; (**c**) F/F0 – 0.0039, AUC – 0.0007; (**d**) F/F0 – 0.4386, AUC – 0.0925. **p < 0.01; ns, not significant.

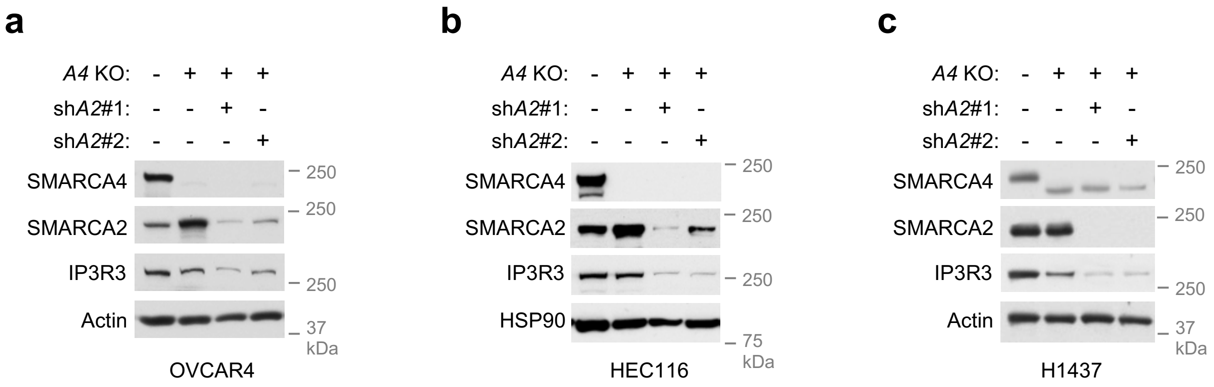

Supplementary Fig. 8 SMARCA4/2 regulate IP3R3 expression in ovarian and lung cancer cells, related to Fig. 3.
Immunoblots of indicated proteins in OVCAR4 (**a**), HEC116 (**b**) and H1437 (**c**) cancer cell lines with indicated *SMARCA4/2* perturbations. *A4*^{KO}: *SMARCA4* knockout; sh*A2*: shRNA targeting *SMARCA2*.

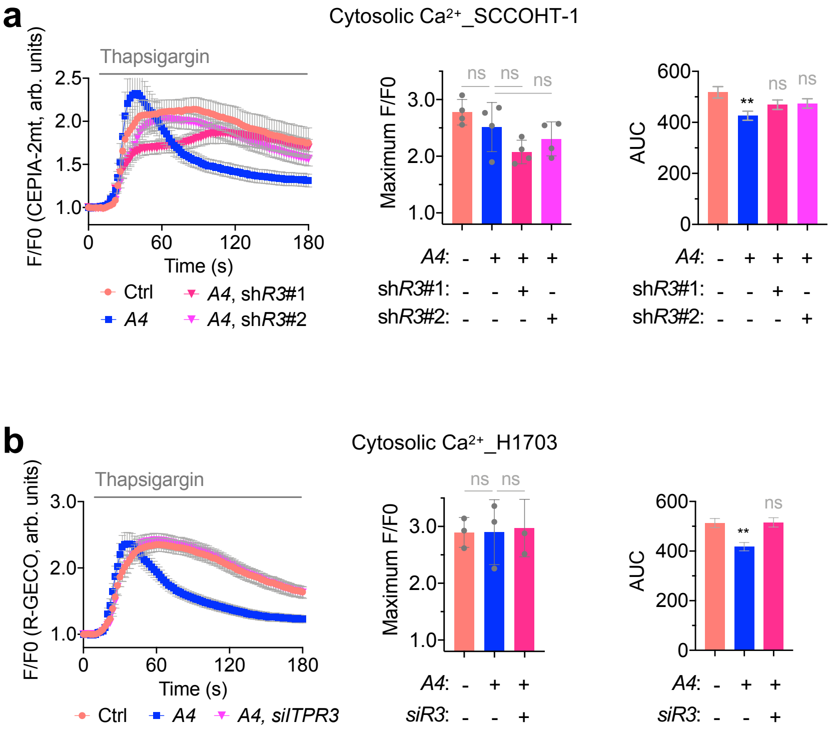

Supplementary Fig. 9 Perturbations of *ITPR3* do not affect Ca^{2+} storage in the ER, related to Fig. 4.
a Changes of cytosolic Ca^{2+} content in SCCOHT-1 cells with indicated *SMARCA4* and *ITPR3* perturbations upon thapsigargin stimulation. 25 Ctrl, 29 *A4*, 52 *A4* Sh*R3*#1, and 58 *A4* Sh*R3*#2 cells from n=4 independent experiments were analyzed. Ctrl: control; *A4*: *SMARCA4*; sh*R3*: shRNA targeting *ITPR3*. Corresponding to Fig. 4a-c. **b** Changes of cytosolic Ca^{2+} content in H1703 cells with indicated *SMARCA4* and *ITPR3* perturbations upon thapsigargin stimulation. 50 Ctrl, 50 *A4*, and 50 *A4* si*R3*#1 cells from n=3 independent experiments were analyzed. Ctrl: control; *A4*: *SMARCA4*; si*R3*: siRNA targeting *ITPR3*. Corresponding to Fig. 4d-f. **a, b,** Left: traces of cytosolic Ca^{2+} content in the indicated cell lines upon 10 μM thapsigargin stimulation (mean ± SEM). Middle: quantification of the maximal Ca^{2+} signal peaks induced by thapsigargin stimulation (mean ± SD). Right: quantification of the area under the curve (AUC) from (**a**) (mean ± SEM). The Ca^{2+} probe R-GECO (R-GECO F/F0) was used to monitor cytosolic Ca^{2+}. Arb. units: arbitrary units. One-way ANOVA followed by Dunnett's tests for multiple comparisons to $A4^{H}A2^{H}$ group; *p*-values (p): (**a**) AUC, *A4* – 0.0018; (**b**) AUC, *A4* – 0.0012. **p < 0.01; ns, not significant.

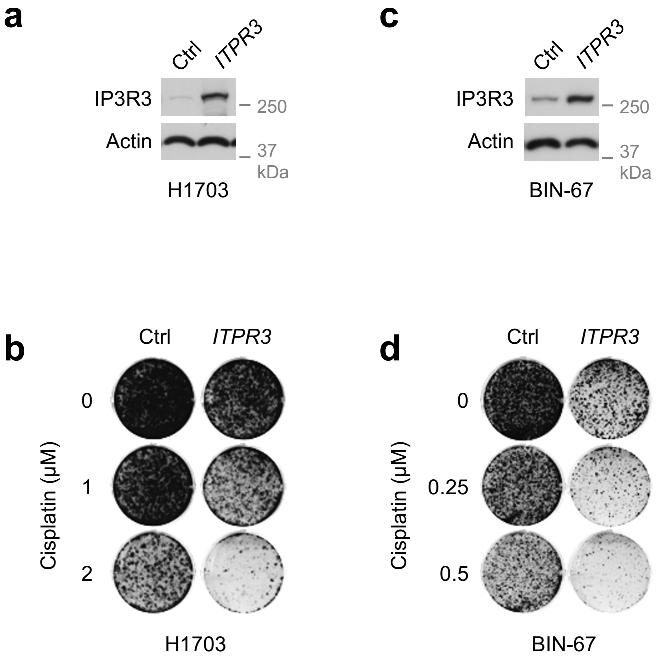

Supplementary Fig. 10 Ectopic expression of *ITPR3* sensitizes SMARCA4/2 deficient cancer cells to cisplatin, related to Fig. 4.

a Immunoblots of H1703 cells with ectopic *ITPR3* expression. **b** Colony formation of H1703 cells with ectopic *ITPR3* expression cultured with indicated cisplatin treatments. Cells were fixed and stained 18 days after plating. Drugs were refreshed every 3 days. **c** Immunoblots of BIN-67 cells with ectopic *ITPR3* restoration. **d** Colony formation of BIN-67 cells with ectopic *ITPR3* expression cultured with indicated cisplatin treatments. Cells were fixed and stained 12 days after plating. Drugs were refreshed every 3 days.

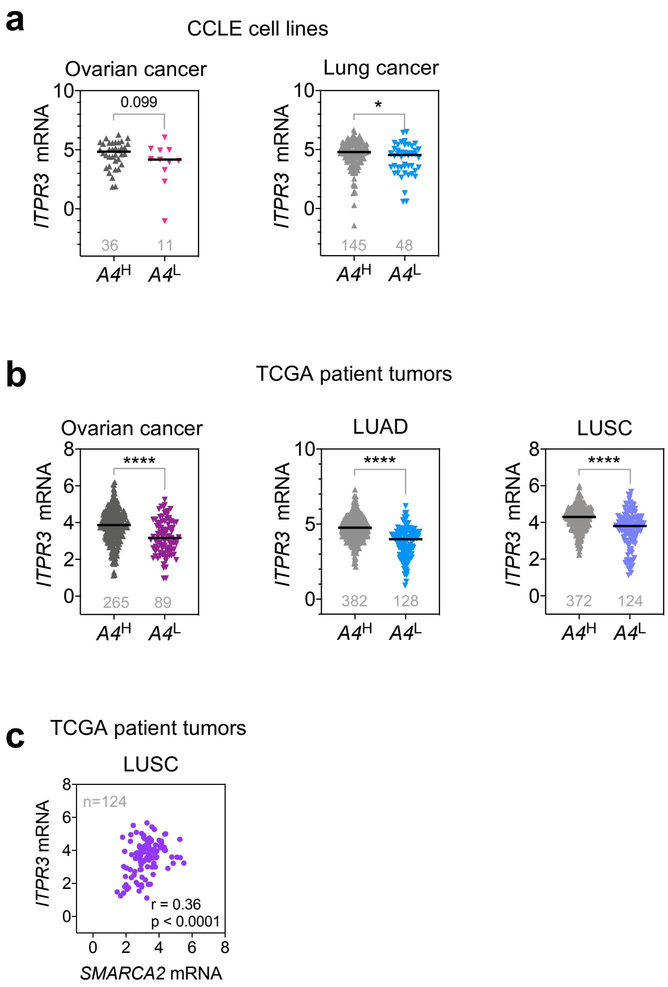

Supplementary Fig. 11 *ITPR3* mRNA expression is reduced in ovarian and lung cancers expressing lower levels of *SMARCA4/2*, related to Fig. 5.

a *ITPR3* mRNA expression in ovarian (left, n=47) and lung (right, n=193) cancer cell lines with differential *SMARCA4* expression. Gene expression data were obtained from Cancer Cell Line Encyclopedia (CCLE) and in Reads Per Kilobase Million (RPKM) [5]. *A4*^{L}: *SMARCA4*^{Low}, cell lines with the bottom quartile of *SMARCA4* expression; *A4*^{H}: *SMARCA4*^{High}, the other cell lines. Number of cell lines is indicated in grey. Two-tailed *t*-test, *p*-values (p): left – 0.099, right – 0.0391. *$p < 0.05$. Corresponding to Fig. 5a. **b** *ITPR3* mRNA expression in ovarian cancer (Left, n=454), lung adenocarcinoma (LUAD, middle, n=510) and Lung Squamous Cell Carcinoma (LUSC, right, n =496) patient tumors with different expression of *SMARCA4*. Gene expression data were obtained from UCSC Xena and in Fragments Per Kilobase Million (FPKM). *A4*^{L}: *SMARCA4*^{Low}, tumors with the bottom quartile of *SMARCA4* expression; *A4*^{H}: *SMARCA4*^{High}, the other cell lines. Number of tumor samples is indicated in grey. Two-tailed *t*-test, *p*-values (p): all < 0.0001. ****$p < 0.0001$. Corresponding to Fig. 5c. **c** Correlation of *ITPR3* and *SMARCA2* mRNA in LUSC (n=124) patient tumors with low expression of *SMARCA4*. Gene expression data were obtained from UCSC Xena and in FPKM. *A4*^{Low}: *SMARCA4*^{Low}, patient tumors with the bottom quartile of SMARCA4 expression. Number of tumor samples is indicated in grey. r, Pearson correlation; p, *p*-value.

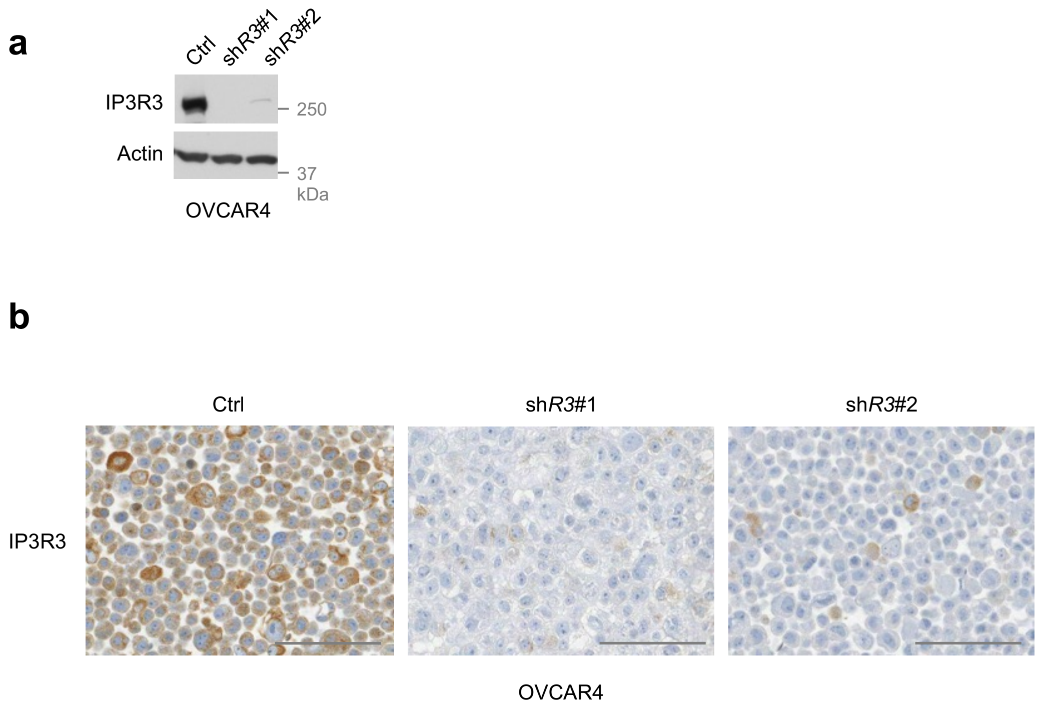

Supplementary Fig. 12 Specificity validation of IP3R3 antibody by shRNA knockdown, related to Fig. 5.
a Immunoblot analysis of IP3R3 protein expression in OVCAR4 cells expressing control vector or shRNAs targeting *ITPR3* (*R3*). **b** Representative images of immunohistochemistry analysis for IP3R3 in OVCAR4 cells described in (**a**). Scale bar, 100 μm.

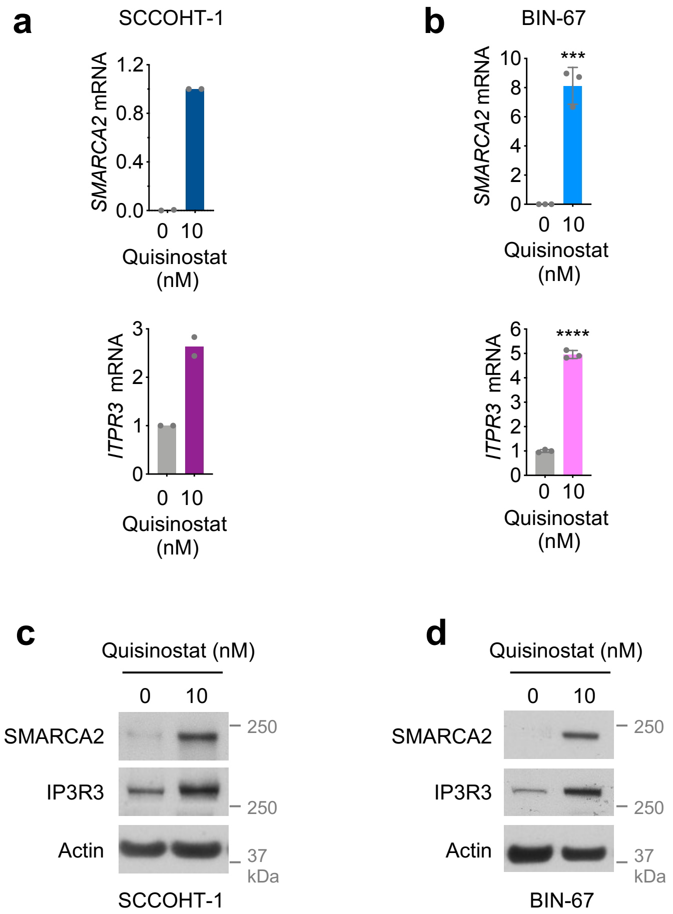

Supplementary Fig. 13 HDAC inhibitor activates *SMARCA2* and *ITPR3* expression in SCCOHT cells, related to Fig. 6.

a, b RT-qPCR measurements of *SMARCA2* (upper) and *ITPR3* (lower) mRNA expression in SCCOHT-1 (**a**) and BIN-67 (**b**) cells treated with quisinostat. Cells were collected 48 hours after the treatment. n = 2 (**a**) or 3 (**b**) independent experiments. Mean ± SD, two-tailed *t*-test (**b**), *p*-values (p): Top - 0.0004, bottom <0.0001. ***p < 0.001, **** p < 0.0001. **c, d** Immunoblot analysis of SMARCA2 and IP3R3 protein expression in BIN-67 (**c**) and SCCOHT-1 (**d**) cells treated with quisinostat for 48 hours.

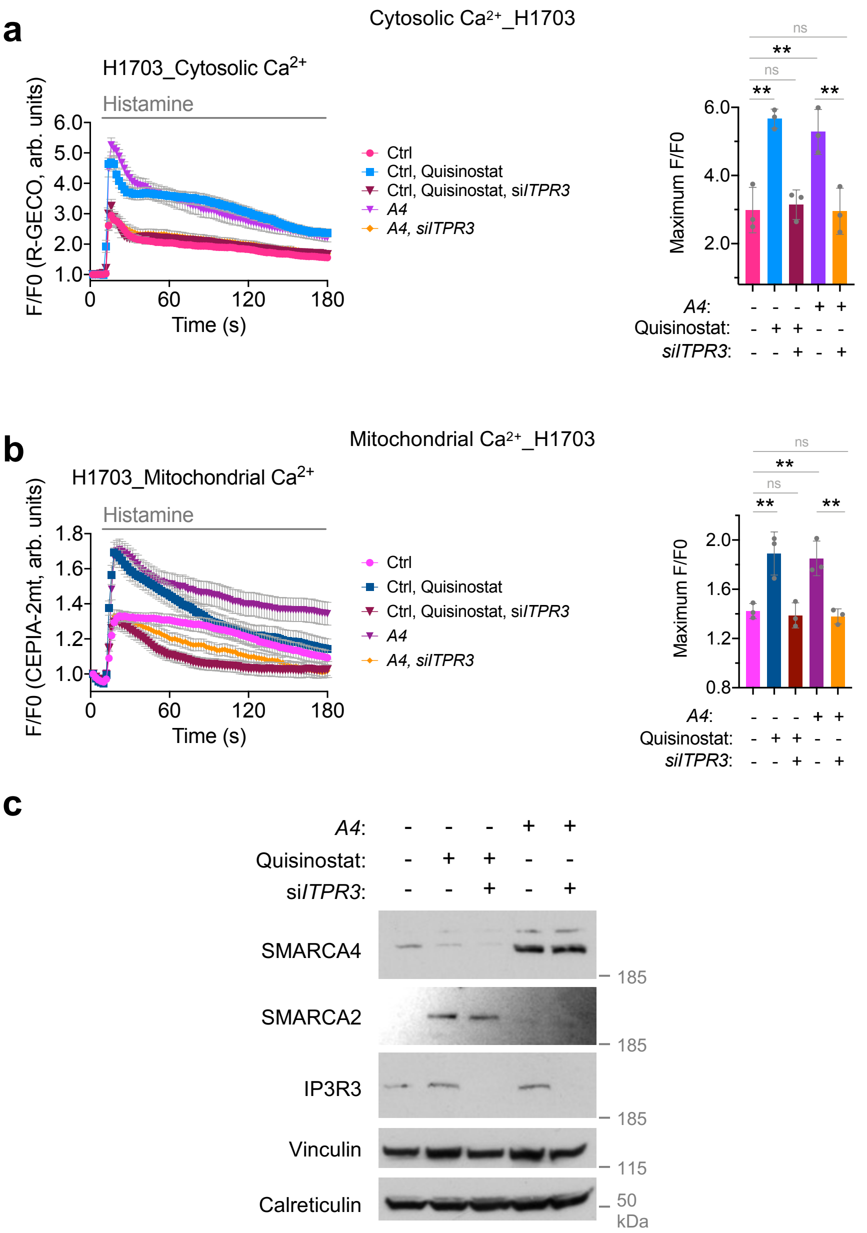

Supplementary Fig. 14 HDAC inhibitor restores Ca^{2+} flux in H1703 cells, related to Fig. 6.
a, b Changes of cytosolic (**a**) and mitochondrial (**b**) Ca^{2+} contents in H1703 cells with indicated *SMARCA4*, *ITPR3* perturbations and quisinostat treatment, upon histamine stimulation. For cytosolic Ca^{2+}, 63 control (Ctrl), 61 Ctrl, quisinostat, 51 Ctrl, quisinostat, si*ITPR3*, 50 *A4* restored, and 53 *A4* restored, si*ITP3R3* cells, from n=3 independent experiments were analyzed. For mitochondrial Ca^{2+}, 50 Ctrl, 51 Ctrl, quisinostat, 58 Ctrl, quisinostat, si*ITPR3*, 52 *A4* restored, and 50 *A4* restored, si*ITP3R3* cells from n=3 independent experiments were analyzed. Quisinostat: 40 nM for 72 hours. Left: traces of cytosolic and mitochondrial Ca^{2+} contents in indicated cell lines upon 100 µM histamine stimulation (mean ± SEM). Right: quantification of the maximal Ca^{2+} signal peaks induced by histamine stimulation (mean ± SD). The Ca^{2+} probes R-GECO (R-GECO F/F0) and CEPIA-2mt (CEPIA-2mt F/F0) were used to monitor cytosolic and mitochondrial Ca^{2+}, respectively. Arb. units: arbitrary units. One-way ANOVA followed by Tukey's multiple comparison test, *p*-values (p): (**a**) Ctrl vs Ctrl_Quisinostat (Qui) – 0.0013, Ctrl vs Qui_si*ITPR3* (si*R3*) – 0.9965, Ctrl vs *A4* – 0.0041, Ctrl vs *A4*_si*R3* >0.9999, *A4* vs *A4*_si*R3* – 0.0037; (**b**) Ctrl vs Ctrl_ Qui – 0.0045, Ctrl vs Qui_ si*R3* – 0.9955, Ctrl vs *A4* – 0.0082, Ctrl vs *A4*_si*R3* – 0.9901, *A4* vs *A4*_si*R3* – 0.0042. **p < 0.01. **c** Immunoblot analysis of the indicated proteins in H1703 cells with indicated *SMARCA4* and *ITP3R3* perturbations and quisinostat treatment. Quisinostat: 40 nM for 72 hours.

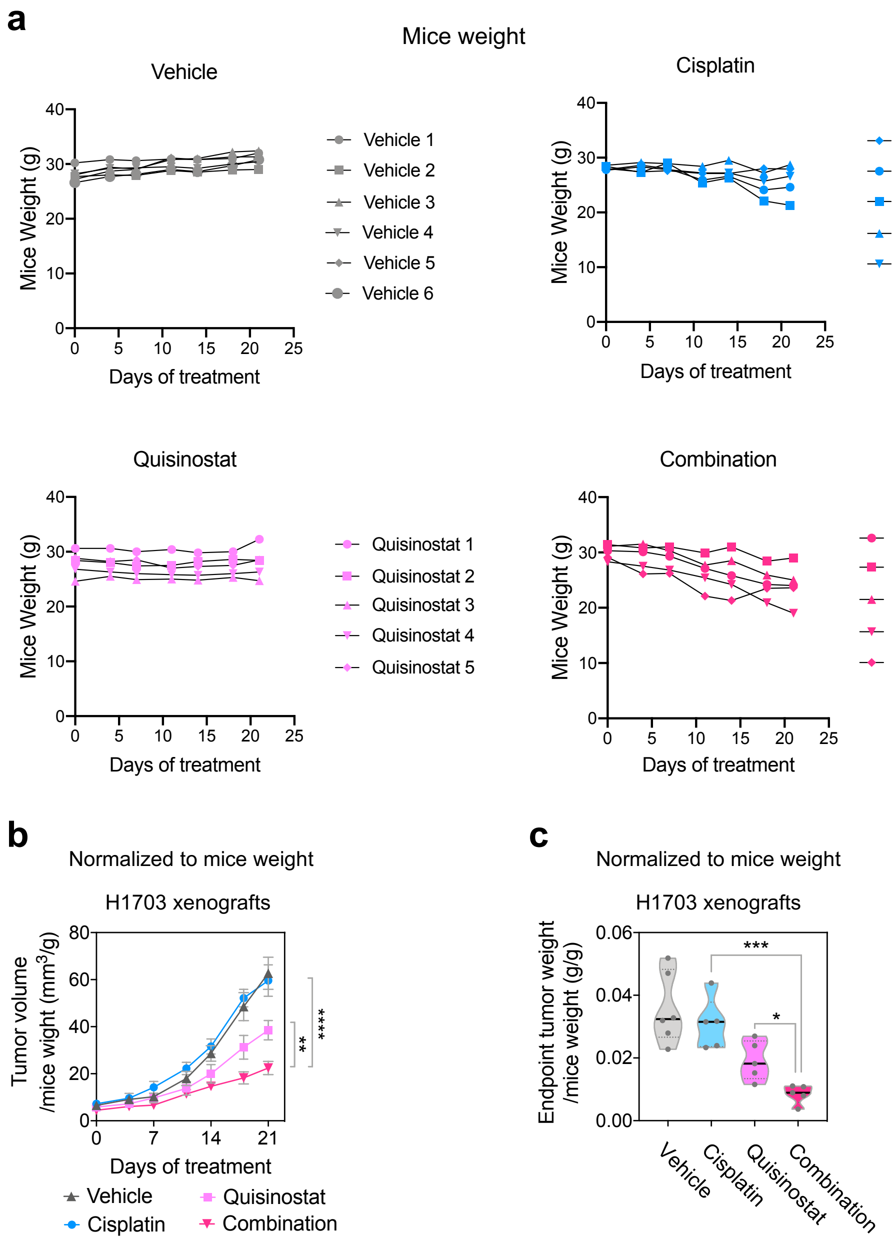

Supplementary Fig. 15 Effects of cisplatin, quisinostat or their combination on tumor growth normalized to body weight of mice, related to Fig. 6.

a Body weight of mice in xenograft models of H1703 cells treated with cisplatin, quisinostat or their combination. **b** Tumor growth normalized to body weight in xenograft models of H1703 cells treated with cisplatin, quisinostat or their combination. Mean ± SEM, vehicle group (n=6 animals), all other groups (n=5 animals), two-way ANOVA, *p*-values (p): cisplatin <0.0001, quisinostat – 0.0058. **p < 0.01, **** p < 0.0001. **c** Final tumor weight normalized to body weight at the end point of the experiment. Vehicle group (n=6 animals), all other groups (n=5 animals). One-way ANOVA followed by Dunnett's tests for multiple comparisons to the combination group, *p*-values (p): lower, cisplatin - 0.0002, quisinostat – 0.0349. *p < 0.05, ***p < 0.001. Corresponding to Fig. 6i.

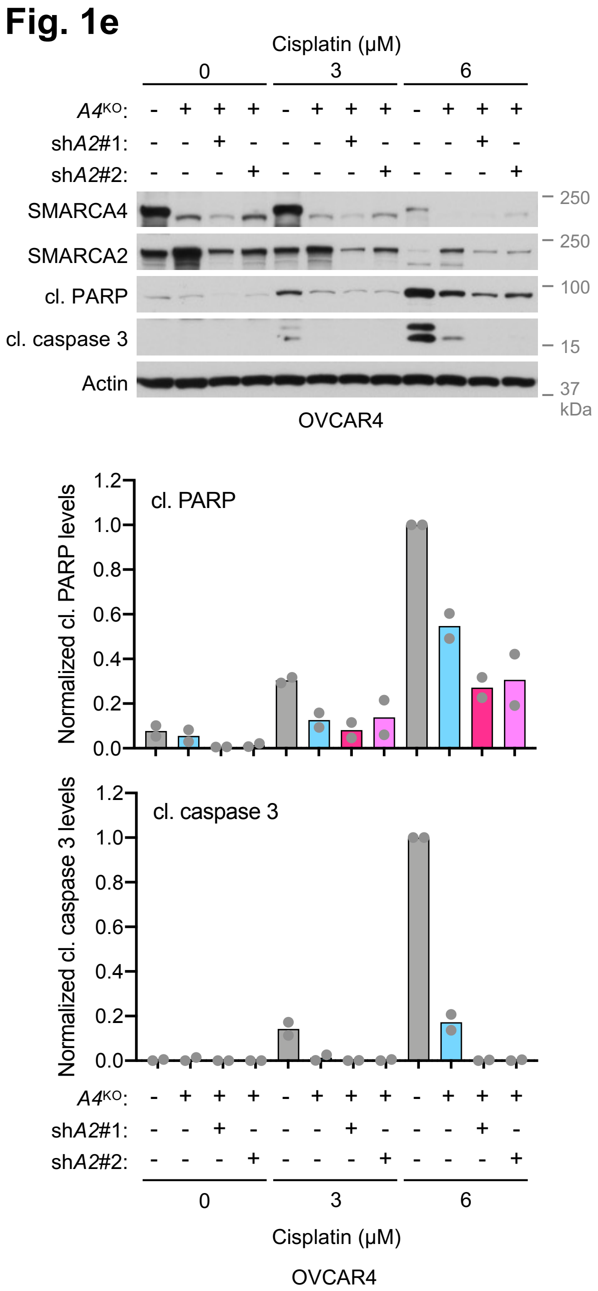

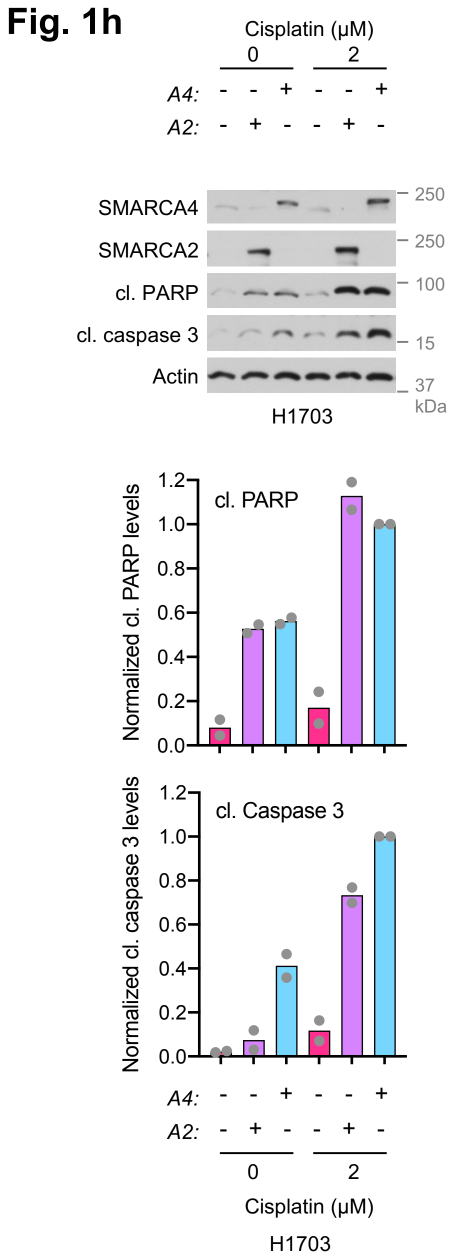

Supplementary Fig. 16a Quantification of key immunoblots in Fig. 1.
The histograms show the quantification of cleaved PARP, cleaved caspase 3 or IP3R3 corresponding to indicated figures. The quantification was performed by ImageJ from n=2 independent representative experiments and normalized to the loading control Actin.

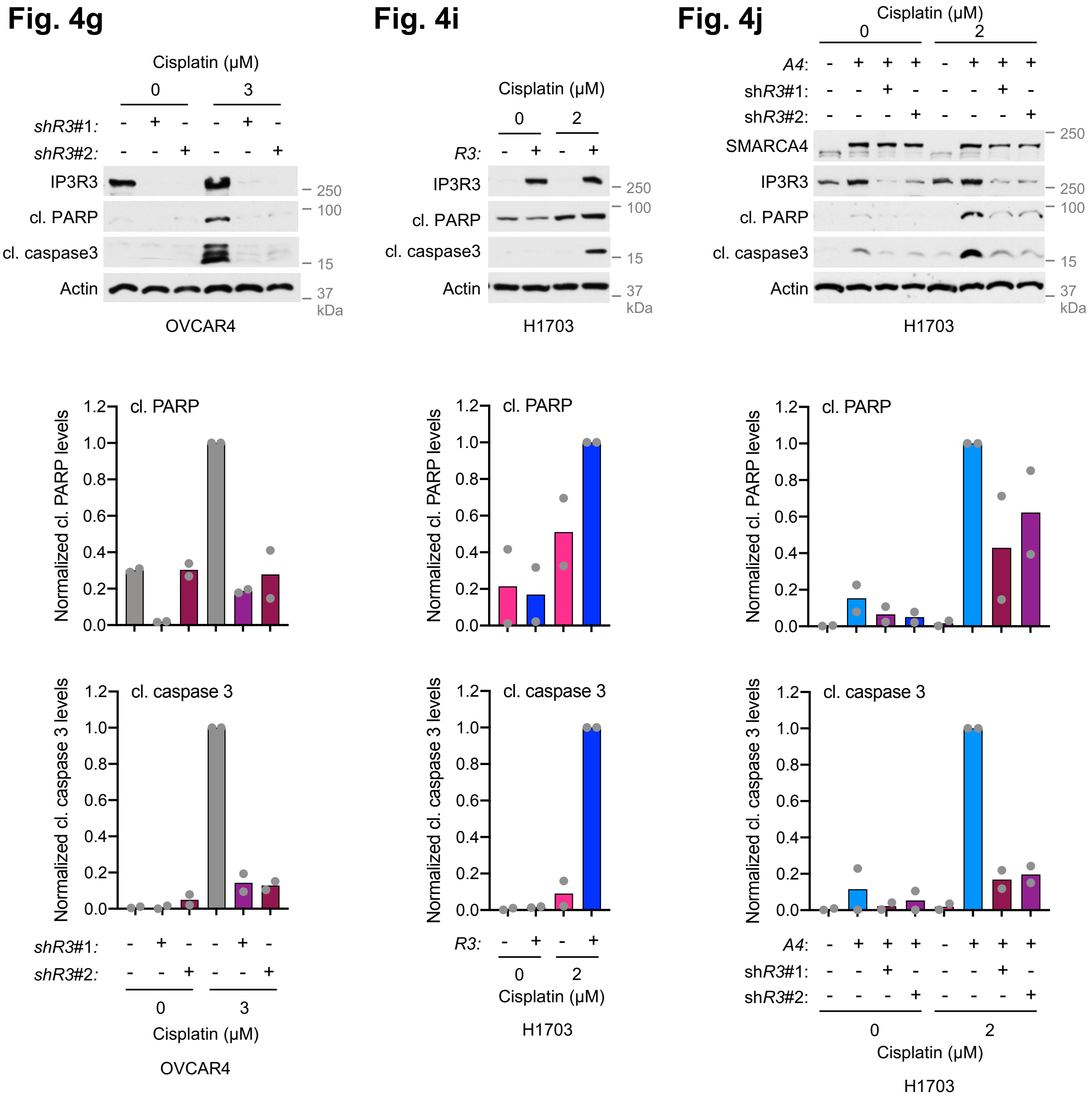

Supplementary Fig. 16b Quantification of key immunoblots in Fig. 4.
The histograms show the quantification of cleaved PARP, cleaved caspase 3 or IP3R3 corresponding to indicated figures. The quantification was performed by ImageJ from n=2 independent representative experiments and normalized to the loading control Actin.

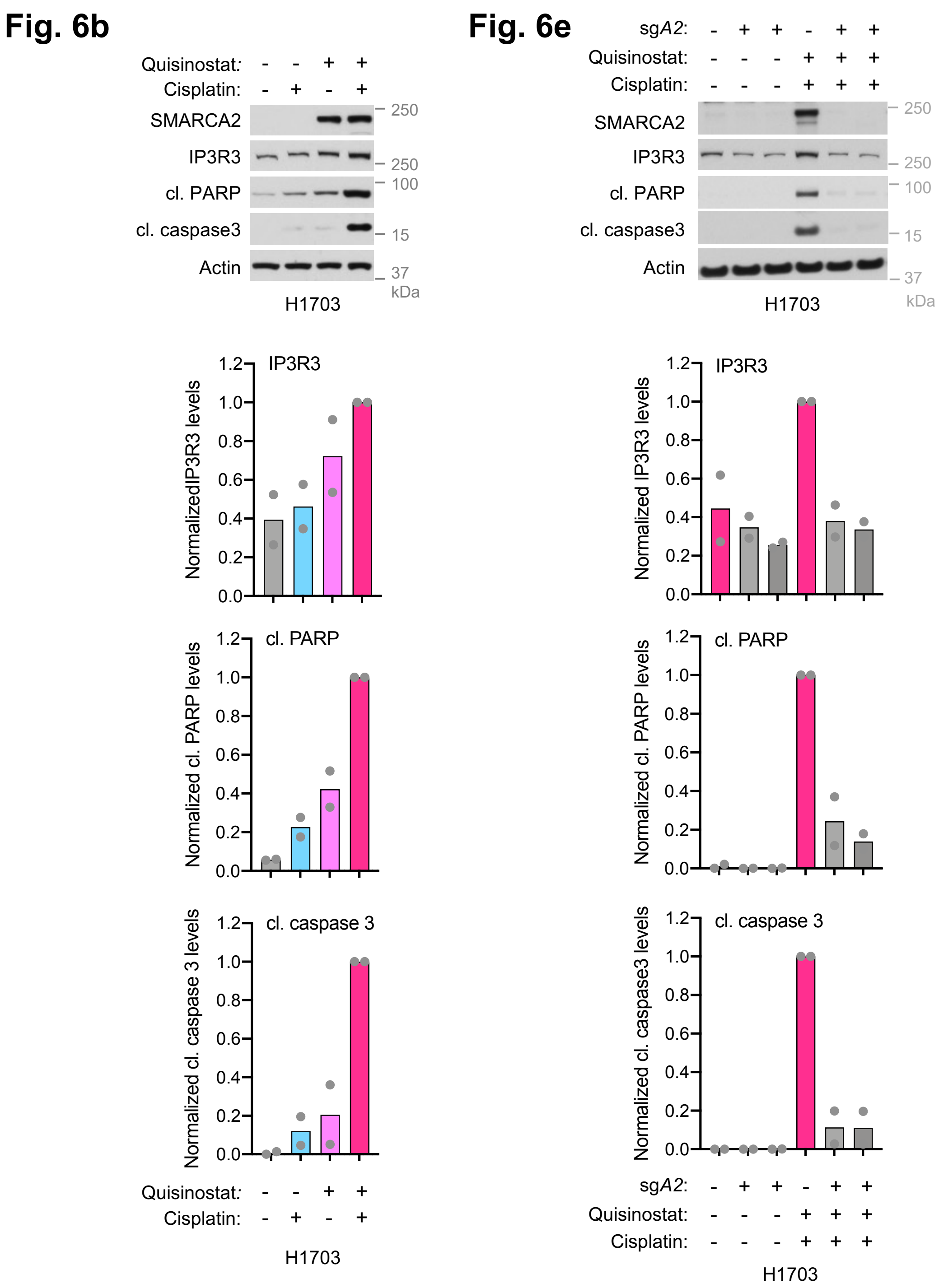

Supplementary Fig. 16c Quantification of key immunoblots in Fig. 6b, e.
The histograms show the quantification of cleaved PARP, cleaved caspase 3 or IP3R3 corresponding to indicated figures. The quantification was performed by ImageJ from n=2 independent representative experiments and normalized to the loading control Actin.

Fig. 6f

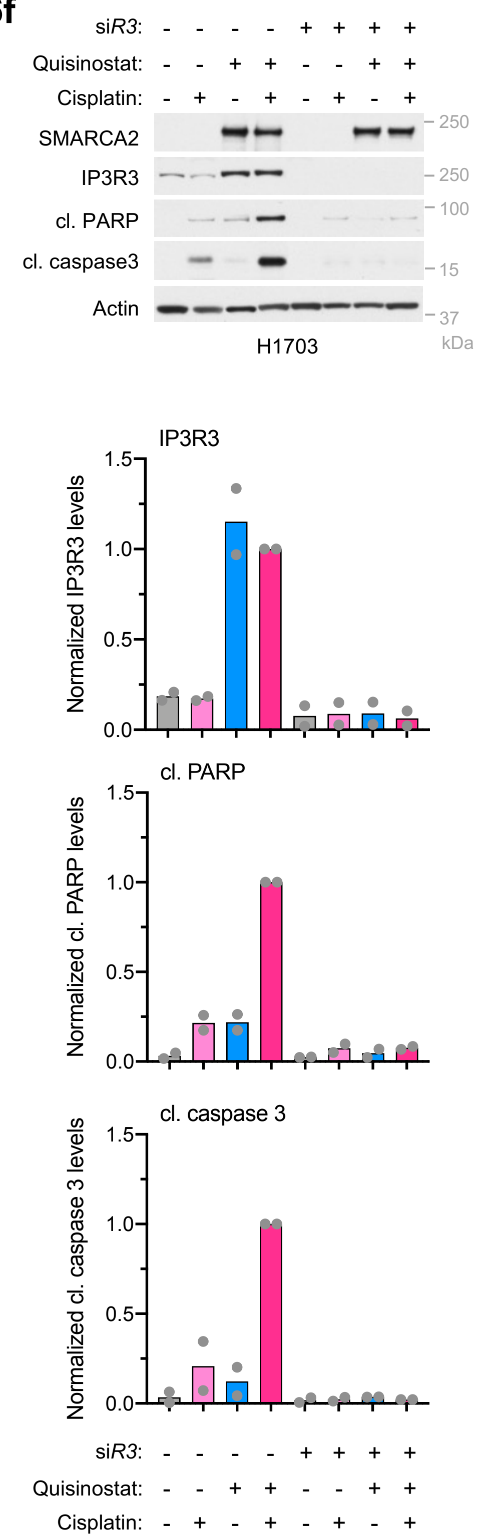

Supplementary Fig. 16d Quantification of key immunoblots in Fig. 6f.
The histograms show the quantification of cleaved PARP, cleaved caspase 3 or IP3R3 corresponding to indicated figures. The quantification was performed by ImageJ from n=2 independent representative experiments and normalized to the loading control Actin.

Fig. 1e

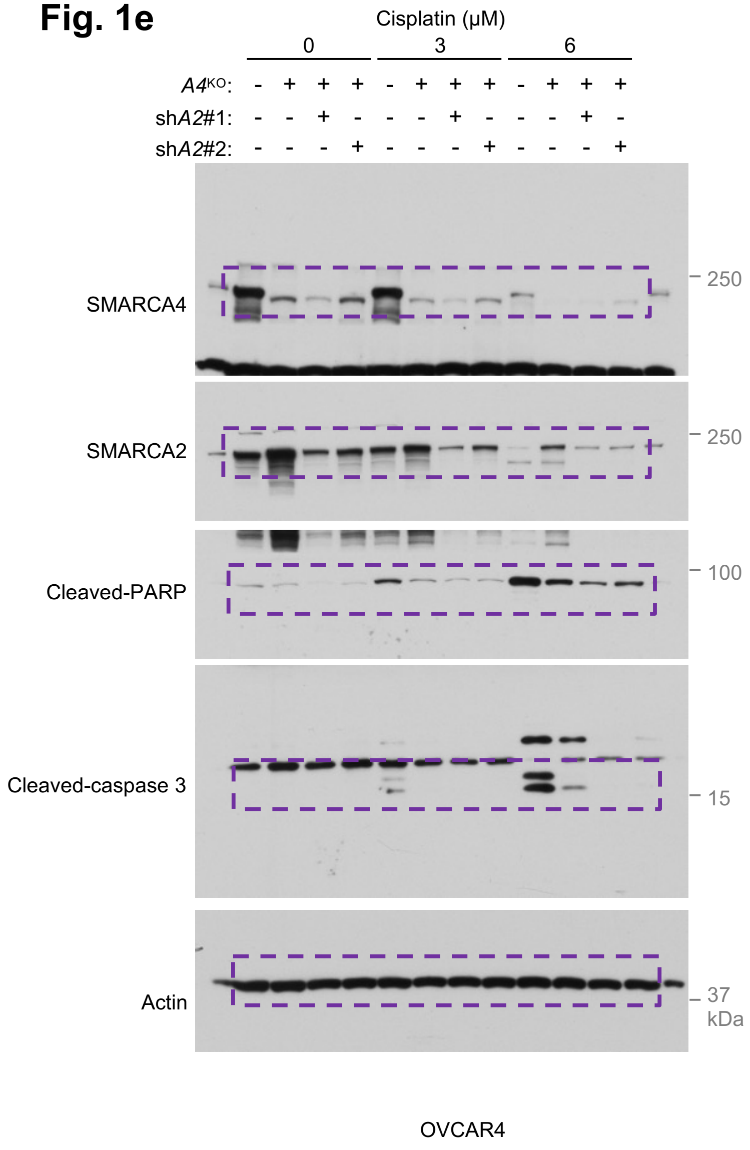

Fig. 1h

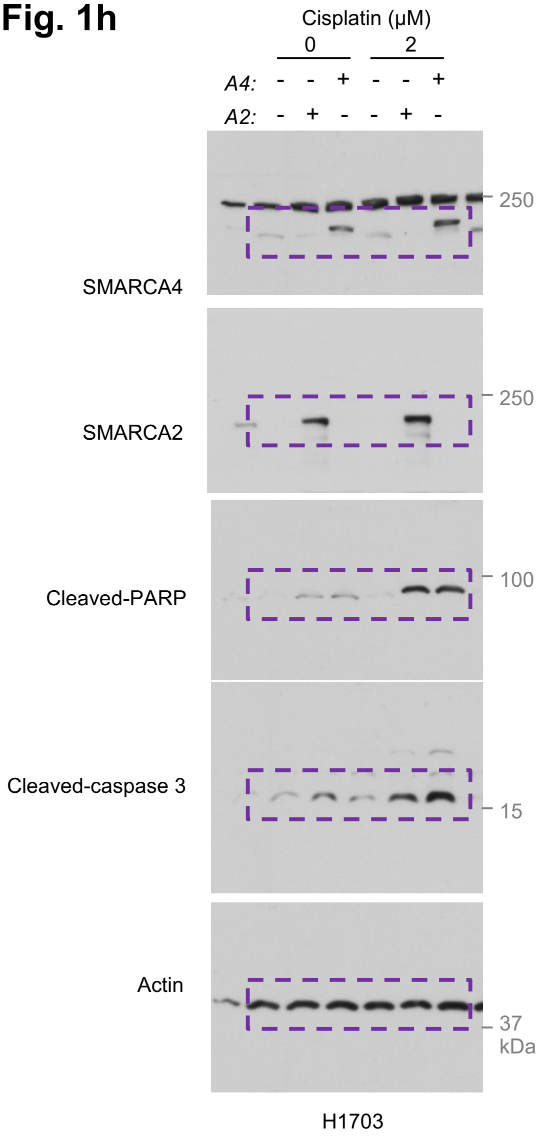

Supplementary Fig. 17a Uncropped scans for the immunoblots presented in Fig. 1.

Fig. 2c, f, i

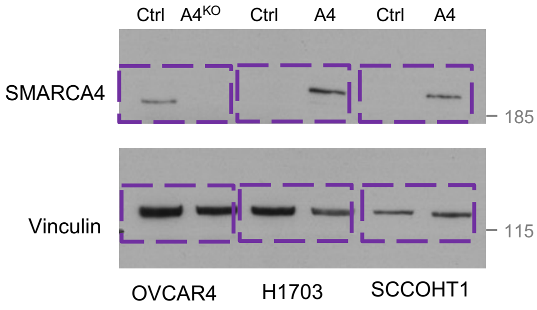

Fig. 2l

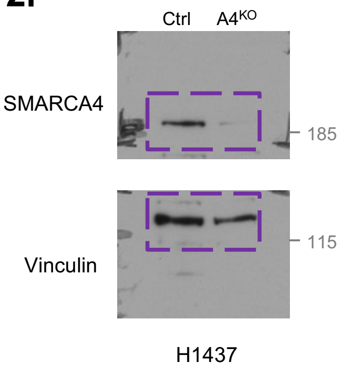

Supplementary Fig. 17b Uncropped scans for the immunoblots presented in Fig. 2.

Fig. 3d

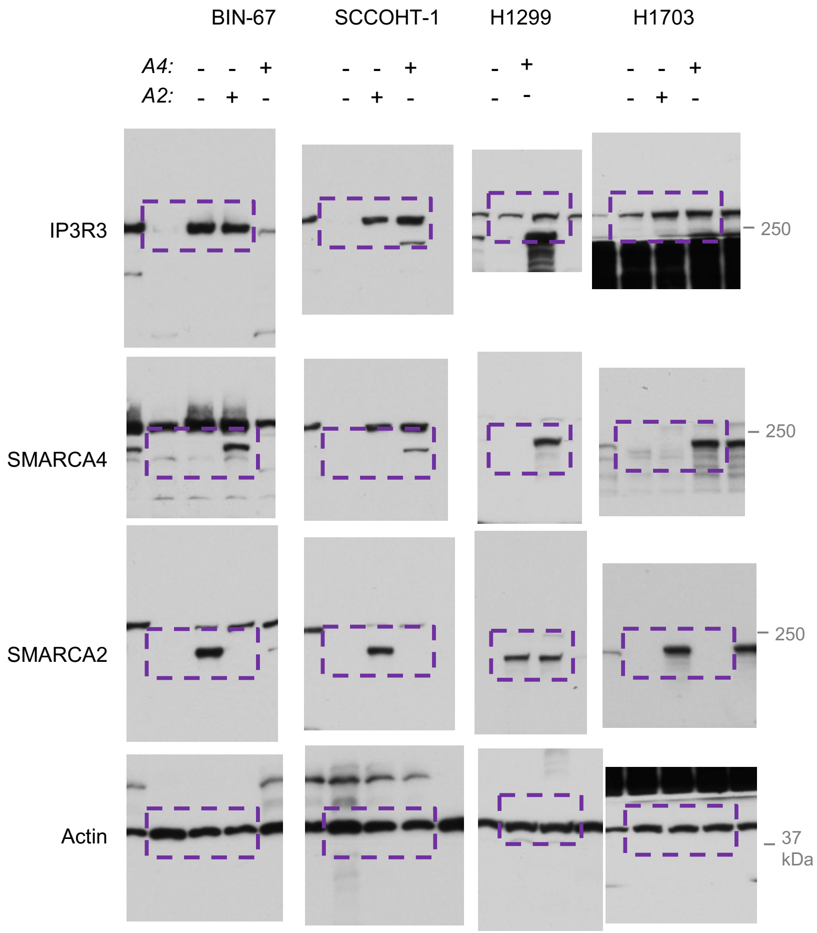

Supplementary Fig. 17c Uncropped scans for the immunoblots presented in Fig. 3.

Fig. 4a

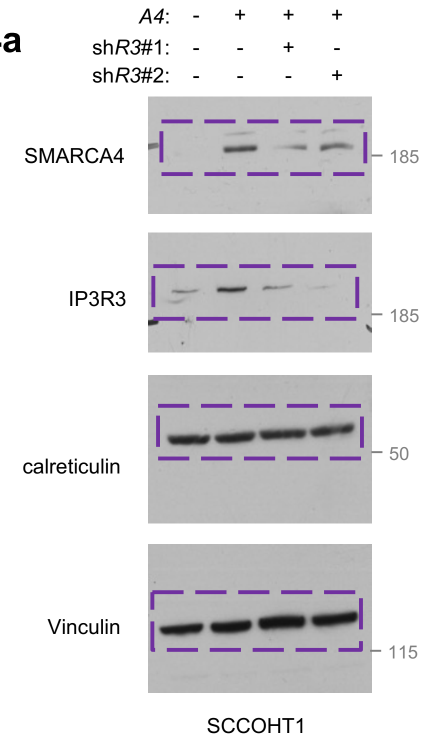

Fig. 4d

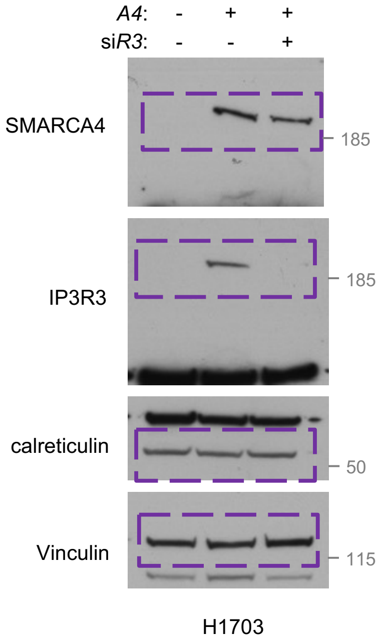

Fig. 4g

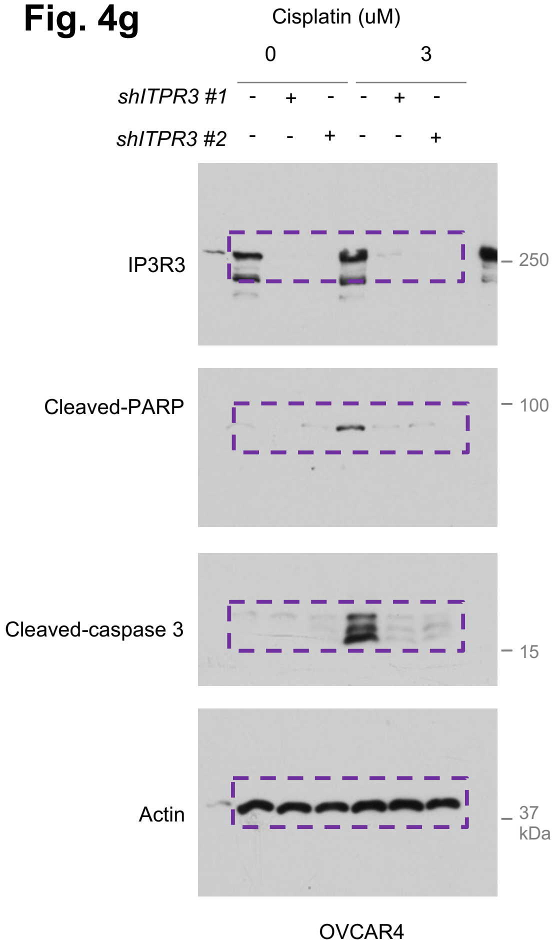

Fig. 4i

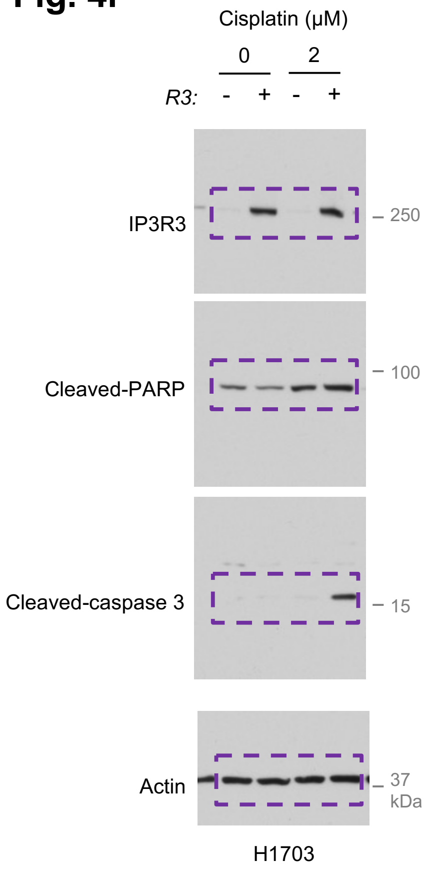

Supplementary Fig. 17d Uncropped scans for the immunoblots presented in Fig. 4.

Fig. 4j

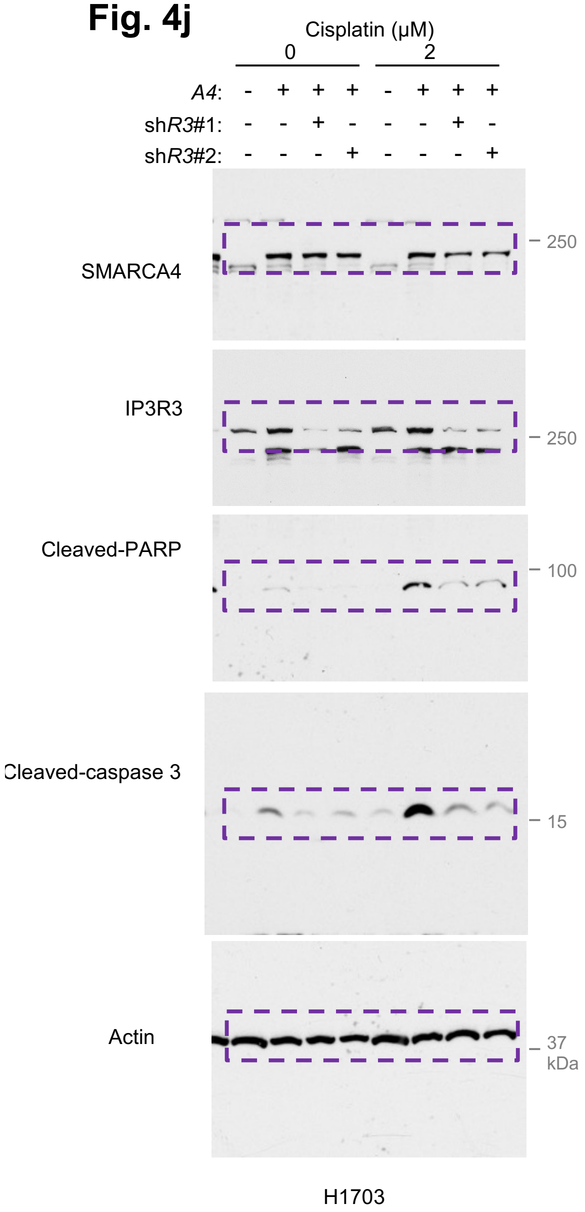

Supplementary Fig. 17e Uncropped scans for the immunoblots presented in Fig. 4.

Fig. 5b

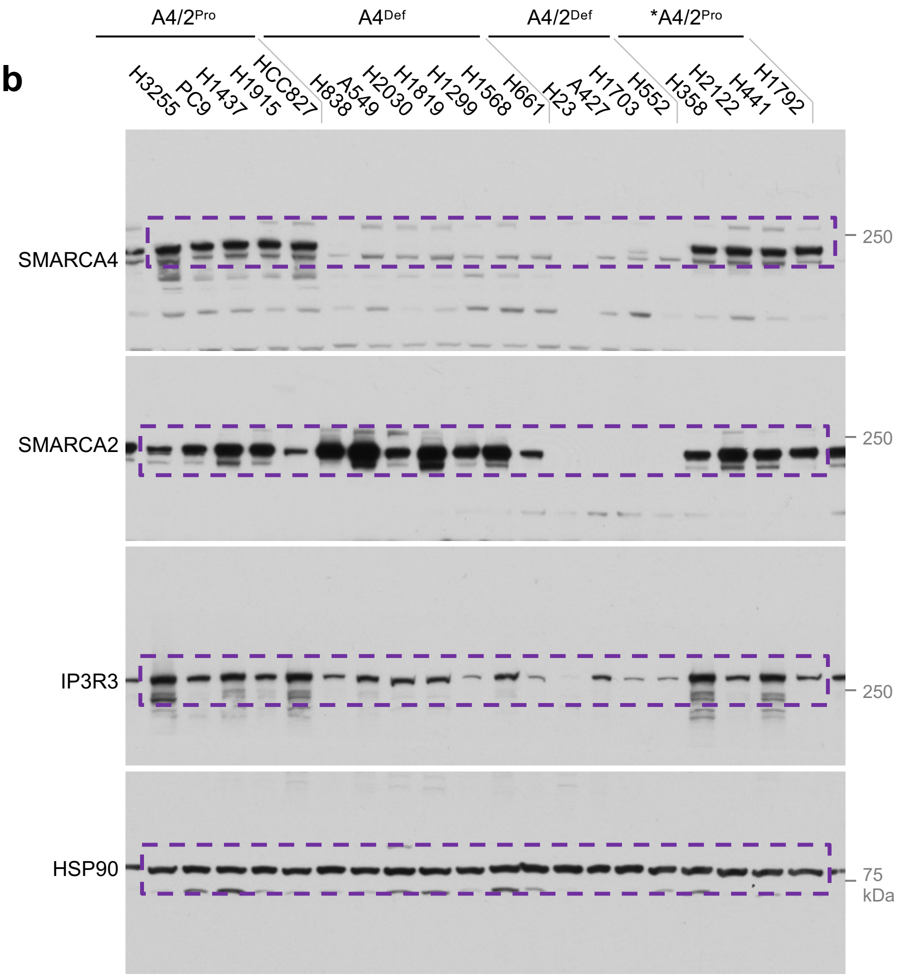

Supplementary Fig. 17f Uncropped scans for the immunoblots presented in Fig. 5.

Fig. 6b

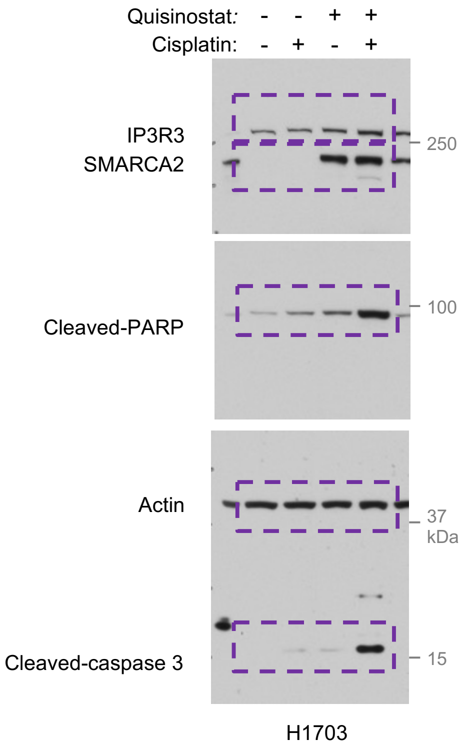

Fig. 6e

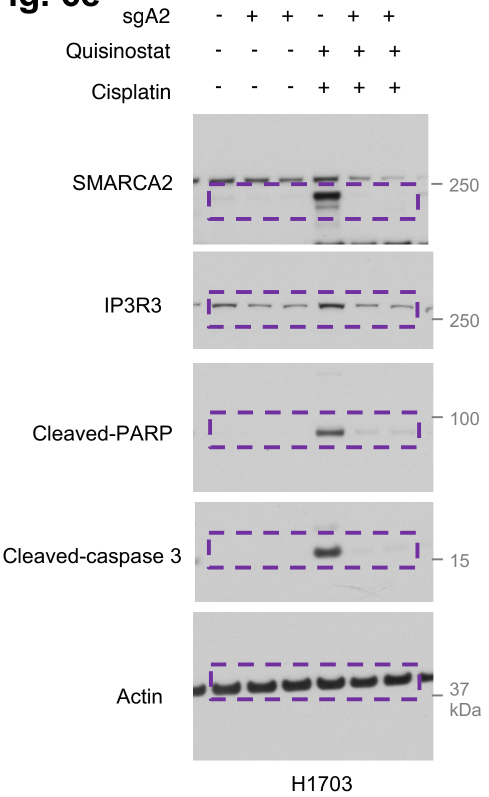

Fig. 6f

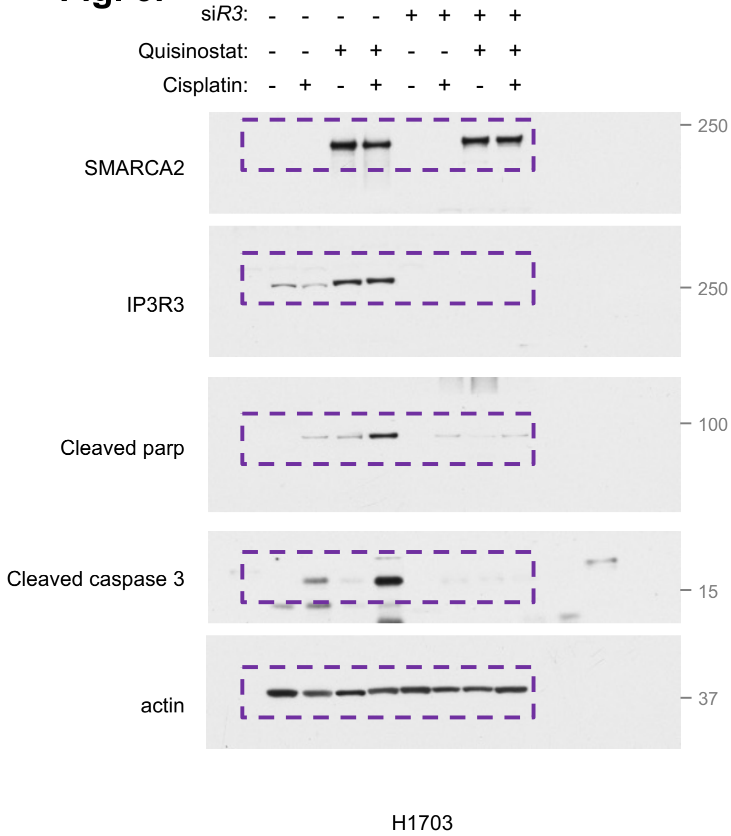

Supplementary Fig. 17g Uncropped scans for the immunoblots presented in Fig. 6.

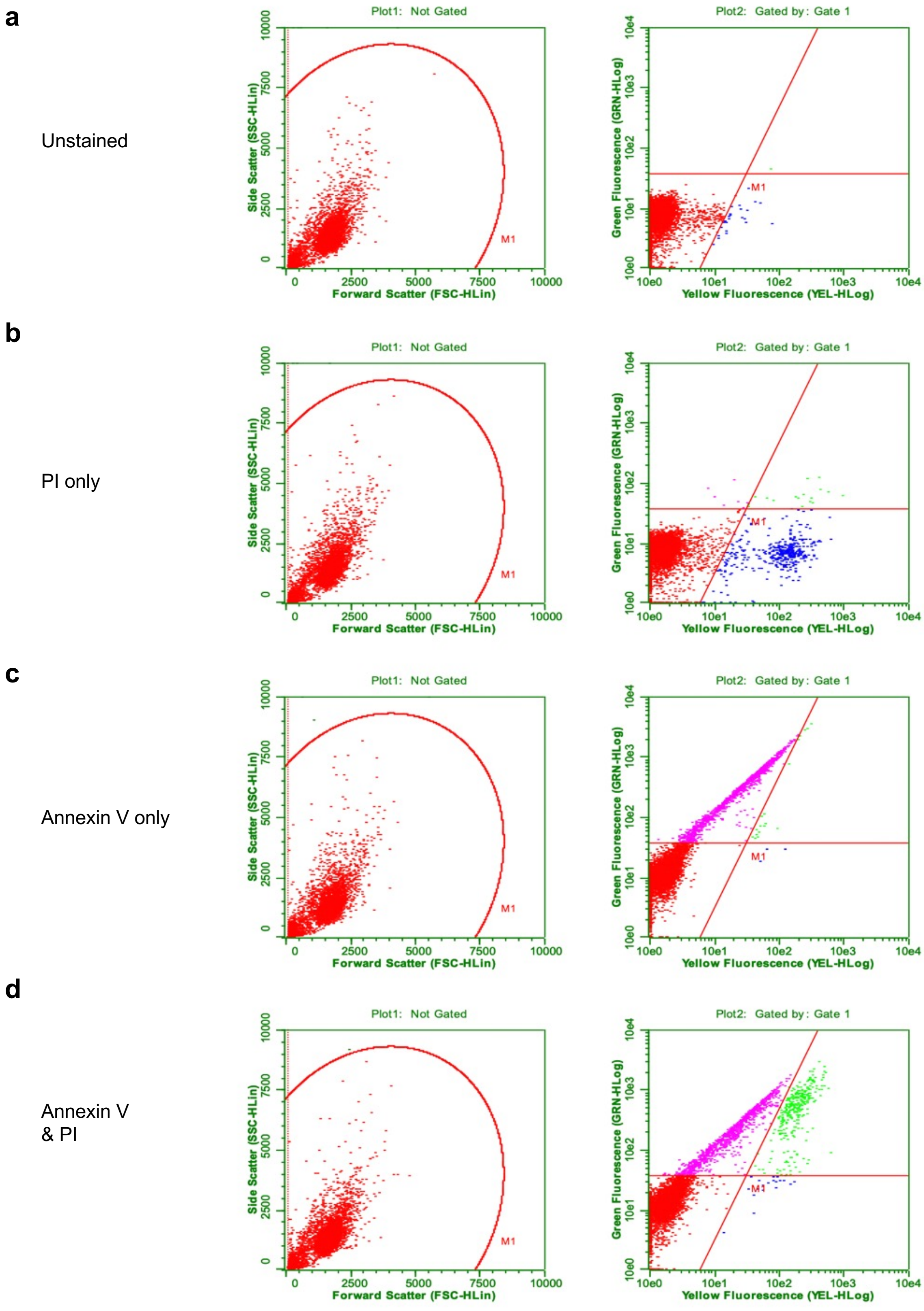

Supplementary Fig. 18 Gating strategy using Guava flow cytometer.
a-d The forward and side scatter gating (left column with the circle) and the fluorescence gating (right column with the 4 quadrants) of H1703 cells from the following conditions: without the addition of annexin V and propidium iodide (PI) fluorescent probes (**a**); with the addition of PI fluorescent probe only (**b**); with the addition of annexin V fluorescent probe only (**c**); with the addition of both annexin V and PI fluorescent probes (**d**). Gating strategy applied to Fig. 1f, i, Fig. 4h, k, Fig. 6c.

Supplementary References

1. Director's Challenge Consortium for the Molecular Classification of Lung A*, et al.* Gene expression-based survival prediction in lung adenocarcinoma: a multi-site, blinded validation study. *Nat Med* **14**, 822-827 (2008).

2. Gyorffy B, Surowiak P, Budczies J, Lanczky A. Online survival analysis software to assess the prognostic value of biomarkers using transcriptomic data in non-small-cell lung cancer. *PLoS ONE* **8**, e82241 (2013).

3. Tang H*, et al.* A 12-gene set predicts survival benefits from adjuvant chemotherapy in non-small cell lung cancer patients. *Clin Cancer Res* **19**, 1577-1586 (2013).

4. Xue Y*, et al.* CDK4/6 inhibitors target SMARCA4-determined cyclin D1 deficiency in hypercalcemic small cell carcinoma of the ovary. *Nat Commun* **10**, 558 (2019).

5. Ghandi M*, et al.* Next-generation characterization of the Cancer Cell Line Encyclopedia. *Nature* **569**, 503-508 (2019).

Supplementary Table 1

Genes (1)	RRA score	Genes (2)	RRA score	Genes (3)	RRA score	Genes (4)	RRA score	Genes (5)	RRA score	Genes (6)	RRA score	Genes (7)	RRA score	Genes (8)	RRA score
EP300	2.67E-09	HDAC1	0.023624	UHRF2	0.090401	AFF1	0.17331	KDM3A	0.28901	BAZ1B	0.42906	NCOA2	0.59619	TADA2A	0.82441
KMT2B	0.0000243	PHF7	0.023885	DPF3	0.092226	ING5	0.17349	SPHK2	0.29206	TDRD10	0.42957	SIRT2	0.59802	LEO1	0.82956
YWHAZ	0.0000436	ARID4B	0.024947	SETDB2	0.093123	ZNHIT3	0.17629	SIRT3	0.29661	TAF9	0.43006	BRD9	0.60399	ATAD1	0.83085
KMT2A	0.000064	CDK9	0.025104	SAP18	0.096263	PHF2	0.1777	CHD6	0.29807	TFDP1	0.43157	ELL2	0.60975	KANSL1	0.83338
CARM1	0.0001188	TDRD3	0.025269	TFPT	0.09646	SUZ12	0.18151	PBRM1	0.29963	PRMT6	0.43281	PRMT2	0.61043	LCOR	0.83559
YWHAE	0.0001207	CRB2	0.025654	PCGF5	0.09863	BMI1	0.18652	TDRD5	0.30113	NAT10	0.43743	LCORL	0.61411	SIRT1	0.84088
TRIM24	0.00019443	PHF20L1	0.029262	ACTR5	0.099062	CBX1	0.18669	BRWD1	0.3061	SIRT4	0.43833	PRMT5	0.61808	KDM4C	0.85247
BRPF1	0.00019715	TDRD6	0.029703	ATXN7L3	0.099444	HINFP	0.19163	C14orf169	0.30663	L3MBTL2	0.44465	MTA2	0.61816	NCOA6	0.85272
NAA60	0.00066005	TBL1Y	0.030374	ACTL6A	0.10104	SRCAP	0.19185	GLYR1	0.30732	KMT2D	0.45425	PHC3	0.61943	BRD1	0.85493
ATAD5	0.00081299	BAZ2B	0.031043	RCOR3	0.10115	TAF1A	0.19325	NAT8L	0.31001	NAT8	0.45501	PRAME	0.62422	SETD1A	0.86493
SMARCA4	0.0010377	BRDT	0.03147	EAF1	0.10245	RNF40	0.19356	YWHAH	0.31029	JMJD4	0.46917	UBE2B	0.6275	HCFC2	0.8658
SUPT20H	0.0014358	KMT2C	0.033125	BPTF	0.10386	MGA	0.19406	SUPT4H1	0.31051	NAA40	0.47086	CHD1	0.62805	JMJD6	0.86789
TADA1	0.0015269	EZH1	0.0333	SUPT5H	0.10517	NSL1	0.19739	FBXL19	0.31148	CENPA	0.47172	HMG20B	0.62907	CLOCK	0.87307
KDM1A	0.0016866	MBD2	0.033926	BRD2	0.1057	RUVBL1	0.19868	PHF19	0.31988	NCOR2	0.47375	HELLS	0.6335	SMARCD2	0.87314
SMARCA5	0.0018939	E2F6	0.03396	GATAD2A	0.10716	HSPBAP1	0.2004	BCORL1	0.32191	MLLT6	0.47534	JADE3	0.63757	ING4	0.87364
CHORDC1	0.001899	UBE2E1	0.035234	TAF12	0.10754	MSL3	0.20239	RSF1	0.32427	HDAC11	0.47539	ATAD2	0.64007	DNMT3B	0.8803
MEN1	0.0020397	CHD7	0.037429	SETD1B	0.10782	HDAC2	0.20435	HDAC7	0.32919	KDM6A	0.47783	MBD1	0.64046	CDYL	0.88319
TAF5L	0.0021228	KAT6A	0.037756	NRBF2	0.11092	SMYD5	0.20546	USP21	0.33353	RBBP4	0.47999	FBXO11	0.64373	FANCL	0.88406
PHB2	0.002161	KAT5	0.038894	CTR9	0.11115	BCOR	0.20561	SAP130	0.33469	C11orf30	0.48189	NCOR1	0.65683	SCMH1	0.88875
RCOR1	0.0023035	UTY	0.03916	WIZ	0.11252	AFF4	0.20981	UHRF1BP1L	0.33559	MBD3L4	0.48435	PHF21B	0.65906	SUPT16H	0.88903
SMARCA1	0.0023462	BRPF3	0.03976	ELL3	0.11383	DPF1	0.21303	STK31	0.33588	KDM4D	0.48467	SETD2	0.66198	DMAP1	0.9
EED	0.0027557	ARID5A	0.040991	SIRT7	0.11444	MORF4L1	0.21386	MLLT3	0.3373	TTF2	0.48612	JADE1	0.66546	NOC2L	0.9067
PRMT7	0.0027997	SMARCAL1	0.041069	CBX2	0.11504	PRMT8	0.21589	ATRX	0.33898	POLD3	0.48647	MBD3L5	0.66948	MSL1	0.91042
PADI2	0.0028217	SUPT7L	0.041336	EHMT1	0.11532	SUV420H2	0.21619	WAC	0.34064	MBIP	0.48761	CBX3	0.67313	UHRF1BP1	0.91173
ASH1L	0.0037223	KDM4B	0.04158	PHF5A	0.11583	NAT14	0.21918	RNF20	0.34321	PWWP2B	0.48781	KDM5C	0.67765	PADI3	0.91752
RAD54B	0.0049241	PHF12	0.04176	AEBP2	0.11598	NCOA7	0.22146	RBBP5	0.34638	EAF2	0.48894	MYSM1	0.6784	SMYD4	0.91796
PHC1	0.0050188	SMARCE1	0.043411	EP400	0.11953	DDX59	0.22352	NFRKB	0.35025	YWHAG	0.48968	CDK2AP1	0.67915	BRD7	0.91883
SAP30	0.0051789	WDR5	0.044249	INO80	0.12	ANP32A	0.22745	KDM5D	0.35053	ATAD3A	0.49552	CHD8	0.68063	PHF11	0.91951
VPS72	0.0065217	ARID4A	0.045749	USP16	0.12062	PHF10	0.23104	GTF3C4	0.35411	NAA50	0.49709	PADI4	0.68122	NAA15	0.92076
SFMBT2	0.0070212	KDM2A	0.048604	SKP1	0.12325	TET2	0.23124	ATXN7	0.35762	FBXO10	0.50221	ELL	0.68434	CHD5	0.93168
ZBTB4	0.007139	PHF3	0.048687	RNF17	0.12343	FXR1	0.23464	INO80E	0.36067	PCGF2	0.50517	RAD54L	0.68507	CXXC1	0.94424
UHRF1	0.0072372	TAF1	0.050208	ARID3A	0.12467	NUP98	0.23674	L3MBTL4	0.3618	BRD3	0.50933	KMT2E	0.70481	SMARCD1	0.94812
KAT7	0.0096453	ACTR6	0.05149	SUV39H2	0.12695	MBTD1	0.23729	HDAC6	0.36189	ZBTB38	0.50991	PHF8	0.70666	CECR2	0.95108
DOT1L	0.010117	KDM1B	0.0527	USP22	0.13128	CCNT1	0.2398	PHF13	0.36439	CDYL2	0.51365	CRB1	0.70674	SMARCC2	0.95668
CXXC4	0.010334	HAT1	0.052729	ING2	0.13294	ZCWPW1	0.24183	SUV39H1	0.36551	L3MBTL3	0.51446	MBD3	0.70722	NCOA5	0.95931
SND1	0.010334	PCGF6	0.053682	NAT9	0.13685	CTBP1	0.24306	TRDMT1	0.36732	AIRE	0.51966	FXR2	0.71255	HDAC5	0.96278
TET3	0.010514	INO80B	0.054319	PHF20	0.13711	CDY2B	0.24664	CHD2	0.37352	MORF4L2	0.52287	PAGR1	0.71792	CBX7	0.96405
CREBBP	0.0115	HCFC1	0.054626	SMARCA2	0.1373	MTA3	0.24667	WDR61	0.37485	DPF2	0.53133	DCAF4	0.72078	BAZ2A	0.96598
EPC1	0.012459	DNMT3A	0.054695	MBD4	0.13935	RAD54L2	0.24775	WDR82	0.37831	TDRD12	0.53187	YEATS2	0.72328	MCRS1	0.97371
MTA1	0.012602	KDM5B	0.055097	ENY2	0.14003	ATAD3B	0.24778	JARID2	0.37959	ZZZ3	0.54046	NCOA1	0.72417	CSNK2A2	0.97445
MBD5	0.013244	ZNF217	0.0564	MLLT1	0.14141	INO80D	0.24878	SUPT3H	0.38559	DNMT1	0.5407	ACTL6B	0.74157	UBE2A	0.97874
DNMT3L	0.013415	WDR48	0.056479	KAT6B	0.14306	RYBP	0.24937	TDRKH	0.38628	SFMBT1	0.54378	TDRD7	0.74334	PHF1	0.98182
MEAF6	0.014396	PHC2	0.058132	CHD3	0.14487	DPY30	0.25265	BRD8	0.38766	LRWD1	0.54739	PRMT3	0.74591	ATAD3C	0.98261
PRDM2	0.014788	KDM3B	0.062656	NAT6	0.14515	SMYD1	0.25308	JMJD1C	0.3901	ARID5B	0.5474	PHF23	0.74984	HDAC3	0.98294
ARID3B	0.015059	MTF2	0.064419	NRIP1	0.14586	TBL1X	0.25627	ACTR8	0.39164	YEATS4	0.54799	CXXC5	0.74986	JADE2	0.98339
SMARCB1	0.015464	PAXIP1	0.065585	ARID1B	0.1496	NCOA4	0.25755	ZNHIT2	0.39209	SMARCC1	0.54888	GMPS	0.75073	GPS2	0.98466
TDRD9	0.015905	CDY1B	0.065684	TDRD1	0.14973	SIRT6	0.25775	CSNK2A1	0.39216	SMARCD3	0.55983	PHF14	0.75108	UCHL5	0.98725
SIN3A	0.016107	TADA3	0.066542	USP11	0.14976	WHSC1L1	0.25935	ARID2	0.39297	CDC73	0.57457	CBX4	0.75432	HDAC10	0.98725
CHD4	0.016158	CRB3	0.067422	SETD7	0.15103	KDM8	0.25996	PADI1	0.39429	MAX	0.57475	POLE3	0.7548	SCML2	0.98879
JMJD8	0.016368	PRMT9	0.067675	PRMT1	0.15123	MECP2	0.26181	KDM7A	0.39693	RUVBL2	0.57553	PRR15L	0.75942	SET	0.99027
L3MBTL1	0.01751	EZH2	0.068998	ARID1A	0.15263	BTAF1	0.26576	SIN3B	0.40119	MLLT10	0.57562	SMARCAD1	0.77594	UBE3A	0.9908
HDGFL1	0.018293	ING3	0.071441	ARID3C	0.15274	EHMT2	0.2689	NAT8B	0.40175	PHF6	0.57682	KAT8	0.77965	HLTF	0.99258
MBD3L1	0.018758	SUV420H1	0.072673	ZNHIT1	0.15447	RNF2	0.26968	NAA16	0.40371	DZIP3	0.57736	RING1	0.78038	TRRAP	0.99356
KDM6B	0.018768	YWHAB	0.073256	KDM5A	0.15466	BAZ1A	0.27001	ING1	0.40529	MBD3L2	0.57829	SUDS3	0.7831	NSD1	0.99472
TAF6L	0.019043	SSRP1	0.074081	MRGBP	0.16477	CBX6	0.27026	SMYD3	0.40686	TP53BP1	0.58025	REST	0.78397	USP3	0.99496
CHRAC1	0.019436	PHF21A	0.07466	RBBP7	0.16777	KDM2B	0.27358	PWWP2A	0.40765	SIRT5	0.58057	WHSC1	0.78516	MSL2	0.99544
YAF2	0.020094	SMYD2	0.074694	DR1	0.16832	ZCWPW2	0.27654	HDAC4	0.40797	TET1	0.58361	CHD9	0.78694	CBX5	0.99641
ZNHIT6	0.020252	MDC1	0.074928	BRD4	0.16929	RCOR2	0.28156	JMJD7	0.40844	KDM4A	0.5844	SETD8	0.79082	NAA30	0.99792
MBD3L3	0.020783	PAF1	0.078495	CCDC101	0.171	KANSL2	0.28401	PCGF3	0.41498	CTBP2	0.58684	RTF1	0.79904	PCGF1	0.99928
HDAC9	0.021183	CBX8	0.085586	KAT2A	0.17101	NCOA3	0.2856	NAA20	0.41868	KAT2B	0.58785	ZBTB33	0.80413	TBL1XR1	0.99988
PADI6	0.02159	MBD6	0.086573	HDAC8	0.17104	GATAD2B	0.28757	USP7	0.42121	KANSL3	0.59169	ASH2L	0.80426		
YWHAQ	0.023306	TAF10	0.088651	CSRP2BP	0.17183	SETDB1	0.28802	INO80C	0.42333	ERCC6	0.59355	SFN	0.8084		

Supplementary Table 1

Ranking of genes by robust rank aggregation (RRA) scores in a CRISPR screen with OVCAR4 cells treated ± cisplatin (100 nM). Cisplatin was refreshed every 3 days for 11 days before harvesting. Data was analysed by the MAGeCK statistical software package. See also Source Data.

Supplementary Table 2

SCCOHT-1_*A4*_Ca^{2+}-related_genes

CASQ1	MYL4	FSTL1	SLIT1	LRP1	RYR3	TRPA1	SLC6A4
SPOCK1	HMCN2	MAN1A1	GNPTAB	KCNK3	GRID1	SLC6A7	SLC6A6
S100A14	ANXA10	TRPM2	PLCH2	SLCO2A1	ANO2	ABCA1	SLC9A7
S100A9	EGF	ANXA2P2	PLS3	ATP1A2	ATP8A1	SLC45A3	CACNA1S
THBD	FAT2	CLGN	DGKA	ABCC3	ATP2B3	GRIN3A	TRPC6
GJB2	SVEP1	ANXA2	ACTN1	SLCO2B1	CLDN4	CFTR	SLC6A20
S100A3	HEG1	RASEF	CIB4	CNGA3	LRRC38	SLC22A13	SLC22A3
PRSS3	RASGRP1	EFHD2	LPCAT2	KCNK12	SLC6A15	SLC51A	SLC8A3
CDHR4	DUOX2	MYO5A	EGFL6	ATP1A4	SLC20A1	SLC12A3	SCN4A
CACNA1E	PCDHGC5	SLIT3	ARSA	GABRE	SLC3A1	SLC28A3	SLC16A2
PADI3	VCAN	CDHR2	SGCA	GRIN2A	SLC1A7	CACNA1D	
DYSF	CDH16	PLS1	MYL9	SCN5A	SLC26A8	TRPC3	
MGP	PITPNM3	RHBDL3	CAPN14	AQP1	SLC4A1	KCNN4	
CLSTN2	DLK1	CAPN8	CBLB	PDE2A	KCNK9	KCNG1	
LTBP1	DNER	NELL1	CUBN	HTR3C	KCND3	KCNQ5	
ADGRE2	ENPP2	GSN	EPDR1	SLC18A3	SLC37A2	SLC1A3	
S100A16	DUOX1	PKD1L2	CAPN3	KCNK1	NIPAL1	SLC2A1	
FBLN5	RET	ANXA9	CDH24	CHRNA9	KCNF1	JPH2	
CALB2	NECAB1	FBLN2	S100A10	CLCA2	ATP2B4	HPN	
FBN1	MEGF6	CIB3	RASGRP3	ADAMTS8	SLC9A1	GABRR2	
CRB2	LTBP2	SNCB	LRP1B	ATP8B1	CACNA1C	FXYD4	
MYL10	OIT3	ASPH	CALML3	SLC7A8	ANO9	SLC22A5	
PAMR1	MAN1C1	SELL	TTN	NIPAL4	KCNC4	SLC16A1	
JAG1	CRTAC1	PPP3CA	NOTCH2	SLC22A4	KCNE5	SLC34A2	
THBS2	RAB44	PKDREJ	PLCZ1	ANO4	LRRC8C	SLC37A1	
PADI4	EHD4	SPOCK2	SYT1	CALHM1	CLCN1	ATP1B1	
ANXA8	LPL	NID2	CLEC3B	KCNMA1	SLC11A1	SLC19A2	
FAT3	ANXA13	NOX5	PADI6	APOL1	GABRA2	SLC16A12	
CAPN11	LTBP3	GCH1	RCN3	CEACAM1	CACNA1I	PKD1L1	
LOXL2	MYL2	CIB1	NCS1	SLC51B	ITPR2	SLC6A17	
THBS1	HSPG2	DLL1	PCDH11X	SLC7A4	SCNN1B	TRPV2	
RPTN	S100A13	CABP1	DST	SHROOM2	SLC44A1	SLC36A2	
S100A4	S100A1	DGKB	PCDH20	SCNN1A	SLC5A3	SLC6A3	
MATN2	PRRG4	NKD1	MMP12	GABRQ	GRIA1	KCNK5	
PCDH12	CAPN2	REPS2	PLA2G4A	ANO1	SLC13A4	KCNAB1	
MYL7	HPCAL1	EHD2	EDEM1	CACNA1G	FXYD3	SLC38A4	
FSTL4	CDHR1	FBN2	EYS	SLC9A9	SLC22A16	ABCC2	
DNASE1L3	KCNIP2	RASGRP4	PLSCR4	P2RX6	P2RX1	CACNG6	
S100A2	S100A6	HPCAL4	AGRN	KCNK13	ANKH	SLC28A1	
ANXA3	PLCB4	CRACR2B	SULF2	KCNH1	SLC30A3	ATP2B1	
DGKG	VSNL1	CALN1	PLCD4	LRRC8E	KCNE1	KCNA10	
SPARC	BMP1	MATN4	ENPP1	SLC38A3	OTOP2	SLC8B1	
SCUBE3	NOTCH4	CDH2	PCDHA2	SLC13A2	CACNB4	CLIC4	
TGM3	HMCN1	C1R	CPNE6	SLC2A9	SLC34A3	SLC5A1	
EDIL3	NPNT	EPS15	CALM2	SCN2A	ITPR3	SLC4A4	
MCTP1	CCBE1	RGN	TPM4	ASIC2	SLC4A9	SFXN3	
EGFLAM	ADGRE5	PAM	CABP4	CATSPER1	RHBG	SLC45A2	
EHD1	EFHC2	ACTN4	PCDHA10	KCNQ3	BEST1	SFXN5	
SMOC1	ADGRV1	UMODL1	ALOX15B	TMEM37	SLC1A2	SLC6A9	
S100A5	VWA2	CLSTN3	OCM	SLC26A9	AZIN2	SLC30A1	

BIN-67_*A4*_Ca^{2+}-related_genes

FAT2	EGFL6	SLIT3	PCDHA12	SLC2A1	CACNA1S	KCNK1
F7	MMP12	ALOX15B	ACTN1	NIPAL4	KCNMA1	SLC22A16
S100A3	OTOF	CRTAC1	DHH	LRRC38	CACNA1I	LRRC26
CLEC3B	ANXA3	PCDHGC4	MEGF6	HTR3A	CACNG6	CNNM4
RPTN	SUSD1	PCDHB15	S100A13	ATP8B1	SLCO3A1	CLCN1
CAPN8	PRF1	ENPP2	CALN1	SLC6A20	GABRR2	KCNJ8
S100A14	FSTL4	MATN3	DLK2	CNGA3	TMEM37	KCNB1
S100A16	DYSF	PKD2L2	SCUBE2	ITPR3	KCNJ12	SLC22A1
SPARC	PAMR1	RYR1	REPS2	CHRNA9	SLC6A19	CLCA1
CDHR4	ANXA1	TTYH1	ANXA2	SCNN1B	SLC6A6	GRIN3B
S100A9	THBD	CASQ2	SMOC1	SLC9A3	KCNA4	CHRNE
SPOCK1	NECAB2	S100A10	KCNIP2	SLC13A4	SLC13A3	ADAMTS8
CRB2	PKDREJ	EGFLAM	S100A11	CLIC5	SLC5A5	SLC4A11
MYL2	HEG1	DNASE1L3	PCDHGA10	SLC2A9	SLC38A3	GRIN2B
RASGRP4	PCDHAC1	EHD4	PITPNM2	SLC18A3	SLC23A1	P2RX6
FLG	LTBP2	TPO	PCDHA13	ATP6V1B1	ANO4	SLC13A1
ANXA8	ACTN4	DAG1	SVEP1	ANO2	CLIC4	CHRND
MYL3	NAALADL1	TTN	CRELD1	CLCA2	P2RX3	PDE2A
NID2	HMCN2	ANXA13	PCDHGC5	LRRC8E	SCNN1G	TTYH3
AOAH	CRNN	EGF	VIL1	CLIC3	ABCA1	KCNAB2
S100A4	TNNC1	S100A1	CDH23	SLC51A	KCNJ5	KCNK10
FBLN2	TRPM2	PCDH1	CAPN3	SLC6A12	KCNK6	ASIC2
CAPN9	MATN2	SMOC2	HRC	SLC22A7	SLC31A2	CLCNKA
PADI1	MYLPF	PCDHA11	DLK1	SLC37A2	ITGAV	RYR3
S100A2	FSTL1	PKD1L2	AIF1	SLC7A4	SLC6A2	SLC44A2
CASQ1	DLL4	CAPS	SLC26A9	SLC28A1	GRIN2C	LRRC8D
CACNA1E	FBN1	CABP4	GABRE	CNGB1	P2RX7	SLC52A1
ANXA9	S100P	THBS4	CEACAM1	KCNA10	MFSD2A	GAS6
PITPNM3	ADGRE1	MAN1C1	SLC12A3	SLC10A6	SLC26A4	KCNQ3
MYL10	ACTN3	ADGRL4	SLC5A1	CATSPER1	ATP1B2	KCNE5
MYL4	CIB3	NID1	KCNK3	TMEM30B	AZIN2	SLC17A2
SELP	SPOCK2	EHD1	KCNE1	SCN4A	CACNG7	SLC39A4
CLSTN2	VWA2	CDH24	JPH2	BEST2	KCND3	ATP1A1
OC90	SGCA	PCDHA10	SLCO2B1	HPN	GRID1	ANO6
CDH16	VWCE	ANXA4	KCNN4	KCNH1	BEST1	SLC25A29
DGKG	BMP1	FBN3	FXYD4	ANO9	SLC1A2	ATP1B1
CAPN11	EHD2	PCDHGA12	AQP1	ATP1A2	LRRC55	KCNAB1
S100A5	CAPN2	PADI4	P2RX1	GRIA1	ATP2B4	KCNQ4
FBLN5	LPCAT2	AGRN	SLCO2A1	HTR3C	SLC30A1	TRPV4
PLCD4	PADI3	CAPN14	SLC51B	SLC13A5	SLC2A10	SLC25A42
TGM3	NOX5	CAPN12	CLDN4	SLC6A4	CACNA2D4	GABRR1
HSPG2	CPNE6	LTBP3	ANO1	SLC16A12	NIPAL3	GJD3
ITLN1	SCUBE1	SLIT1	SLC7A8	CALHM1	ZACN	KCNJ16
MASP1	EEF2K	NOTCH1	APOL1	SLC26A8	ATP2B2	KCNC4
GSN	OCM	PCDHAC2	SCNN1A	SLC1A7	SLC22A14	
MYL7	PADI2	EFCAB12	GABRP	ATP2B3	BSND	
RASGRP3	NOTCH3	PLSCR4	PKD1L1	SLC5A9	GLRA4	
DUOX1	MGP	PCDHGB7	FXYD3	CACNA1G	SLC22A4	
F10	DGKA	SULF2	SLC16A8	SLC16A3	SLC6A3	
PLCB2	PCDHB4	MYL9	ABCC3	SLC12A2	SLC28A3	

BIN-67_SMARCA4_ChIP_Peaks

OR4F29	PINK1	KIAA0319L	JUN	SLC44A3	NOTCH2NLA	DEDD	NEK7	LINC00467	LOC339529	ITGB1	ADAMTS14	KIF11	GRK5	RASSF10	AMBRA1
LINC01128	SH2D5	TRAPPC3	LINC01135	CNN3	ANKRD35	FCGR2A	ATP6V1G3	SLC30A1	ZBTB18	NRP1	PCBD1	EXOC6	MIR4681	ARNTL	LRP4
AGRN	HP1BP3	STK40	HSD52	ALG14	RNF115	FCRLB	NR5A2	NEK2	C1orf100	PARD3	UNC5B	MYOF	TIAL1	SPON1	C11orf49
RNF223	EIF4G3	GRIK3	FGGY	TMEM56-RWDD3	GPR89A	OLFML2B	CAMSAP2	LPGAT1	DESI2	CUL2	UNC5B-AS1	FRA10AC1	BAG3	RRAS2	DDB2
MXRA8	ECE1	LINC01137	MIR4711	PTBP2	PRKAB2	MIR4654	TMEM9	PPP2R5A	EFCAB2	CREM	VSIR	PLCE1	INPP5F	CYP2R1	SLC39A13
GNB1	NBPF3	INPP5B	NFIA	DPYD-AS1	CHD1L	MIR556	PKP1	NENF	KIF26B	CCNY	ANAPC16	TBC1D12	MCMBP	CALCA	NUP160
CALML6	ALPL	MIR3659	KANK4	MIR137HG	BCL9	DDR2	TNNI1	ATF3	SMYD3	GJD4	CHST3	PDLIM1	TACC2	INSC	PTPRJ
GABRD	RAP1GAP	MACF1	DOCK7	LINC01776	GJA5	RGS4	PHLDA3	TATDN3	SCCPDH	FZD8	ASCC1	SORBS1	BTBD16	SOX6	TRIM49B
KCNAB2	USP48	KIAA0754	UBE2U	SNX7	GPR89B	RGS5	CSRP1	RPS6KC1	ZNF496	ANKRD30A	DDIT4	CCNJ	PLEKHA1	PLEKHA7	OR4C46
CHD5	HSPG2	PABPC4	CACHD1	MFSD14A	LINC01138	TMCO1	MIR5191	PTPN14	SH3BP5L	MTRNR2L7	NUDT13	TLL2	HMX3	PIK3C2A	TRIM48
RNF207	CDC42	BMP8B	RAVER2	CDC14A	NBPF8	MIR3658	IPO9	KCNK2	PGBD2	ZNF33BP1	PPP3CB	TM9SF3	CHST15	KCNC1	OR5AK4P
HES3	EPHB2	TMCO2	JAK1	COL11A1	NBPF14	FAM78B	ELF3	SPATA17	DIP2C	LINC00999	USP54	LCOR	EEF1AKMT2	SERGEF	TNKS1BP1
PLEKHG5	MIR4253	ZMPSTE24	DNAJC6	RNPC3	NBPF15	POGK	GPR37L1	TGFB2-AS1	PFKP	ACTR3BP5	ZSWIM8	ANKRD2	CTBP2	LDHAL6A	CTNND1
KLHL21	KDM1A	NFYC	LEPROT	VAV3	PPIAL4E	POU2F1	ARL8A	RAB3GAP2	PITRM1	LOC441666	CAMK2G	AVPI1	TEX36-AS1	PTPN5	STX3
CAMTA1	MIR3115	CTPS1	PDE4B	NBPF6	LSP1P5	MPZL1	LGR6	MARK1	KLF6	BMS1	KAT6B	MARVELD1	LOC283038	MRGPRX2	TCN1
SLC45A1	HTR1D	SCMH1	SGIP1	CLCC1	LINC02591	MPC2	UBE2T	HLX	UCN3	CSGALNACT2	DUPD1	GOLGA7B	EDRF1-DT	NAV2	MS4A10
RERE	ZNF436	EDN2	MIER1	TAF13	FCGR1CP	GPR161	PPP1R12B	DUSP10	IL15RA	LINC00840	ZNF503-AS2	LOXL4	FANK1	LOC100126784	TMEM109
ENO1	ID3	HIVEP3	SLC35D1	KIAA1324	LINC00869	TBX19	SYT2	HHIPL2	PFKFB3	C10orf142	KCNMA1	HPS1	INSYN2A	FIBIN	TMEM132A
ENO1-AS1	MDS2	GUCA2A	GADD45A	EPS8L3	LINC00623	LOC100505918	CYB5R1	TLR5	PRKCQ-AS1	TMEM72	DLG5	CNNM1	PTPRE	BBOX1	VWCE
SLC2A7	LOC284632	SLC2A1-AS1	GNG12	CSF1	HIST2H2AA4	ATP1B1	MYBPH	CCDC185	SFTA1P	RASSF4	DLG5-AS1	GOT1	GLRX3	CCDC34	DDB1
MIR34A	GRHL3	SLC2A1	RPE65	AHCYL1	MTMR11	NME7	CHIT1	CAPN2	LINC00710	SYT15	LINC00856	ABCC2	LINC01164	LGR4	BEST1
SPSB1	STPG1	EBNA1BP2	SRSF11	KCNC4	OTUD7B	GORAB	LINC01136	TP53BP2	USP6NL	GPRIN2	LINC00595	DNMBP	PWWP2B	BDNF	FTH1
CLSTN1	SRRM1	ELOVL1	AK5	CYMP	PLEKHO1	PRRC2C	BTG2	FBXO28	PROSER2	NPY4R	ZMIZ1-AS1	ERLIN1	INPP5A	ELP4	INCENP
UBE4B	STMN1	HYI	ZZZ3	KCNA3	ADAMTSL4	VAMP4	FMOD	MIR320B2	PROSER2-AS1	LINC00842	ZMIZ1	SCD	CYP2E1	PAX6	SCGB1D2
KIF1B	CRYBG2	ST3GAL3	GIPC2	RAP1A	HORMAD1	DNM3	OPTC	LBR	SEPHS1	ANXA8	PPIF	OLMALINC	SCGB1C1	RCN1	SCGB1D4
PEX14	LIN28A	ARTN	ADGRL2	LINC01160	CERS2	DNM3OS	ATP2B4	ENAH	BEND7	ZNF488	ANXA11	PAX2	IFITM1	EIF3M	SCGB1A1
CASZ1	RPS6KA1	IPO13	PRKACB	CTTNBP2NL	MLLT11	PIGC	LAX1	SRP9	FRMD4A	FAM25C	PRXL2A	KAZALD1	IFITM3	QSER1	AHNAK
C1orf127	ARID1A	ERI3	SAMD13	WNT2B	PIP5K1A	TNFSF18	SNRPE	EPHX1	NMT2	FRMPD2	SH2D4B	BTRC	TALDO1	DEPDC7	EEF1G
SRM	SFN	MIR5584	GNG5	MOV10	PI4KB	TNFSF4	SOX13	H3F3AP4	FAM171A1	DRGX	NRG3	NT5C2	CD151	TCP11L1	TUT1
MTOR	TRNP1	KIF2C	SSX2IP	RHOC	CGN	LOC100506023	PLEKHA6	LIN9	PTER	CHAT	CCSER2	RPEL1	MUC5B	HIPK3	INTS5
DISP3	TENT5B	ZSWIM5	LPAR3	AKR7A2P1	C2CD4D	PRDX6	PPP1R15B	ITPKB	RSU1	AGAP7P	GRID1-AS1	NEURL1	BRSK2	KIAA1549L	WDR74
DRAXIN	GPR3	LINC01144	SYDE2	SLC16A1	THEM4	RC3H1	LRRN2	COQ8A	CUBN	SGMS1	MIR346	SH3PXD2A-AS1	LSP1	C11orf91	PLAAT2
MTHFR	SCARNA1	PRDX1	C1orf52	LRIG2	S100A10	KIAA0040	RBBP5	PRSS38	VIM	ZWINT	GRID1	SH3PXD2A	CARS	CD59	SPINDOC
NPPB	EYA3	PIK3R3	BCL10	MAGI3	RPTN	RASAL2-AS1	DSTYK	WNT9A	ST8SIA6	IPMK	WAPL	STN1	OSBPL5	LMO2	CCDC88B
SNORA59B	PTAFR	CYP4A22	DDAH1	PHTF1	CRNN	RASAL2	TMCC2	DUSP5P1	HACD1	TFAM	BMPR1A	COL17A1	APBB1	NAT10	NRXN2
DHRS3	TMEM200B	FOXD2	CCN1	HIPK1	S100A7A	C1orf220	NUAK2	RHOU	STAM	SLC16A9	AGAP11	SFR1	HPX	ABTB2	SF1
KAZN	PTPRU	TRABD2B	SH3GLB1	TSPAN2	S100A7	TOR1AIP1	LEMD1-AS1	RAB4A	NSUN6	CCDC6	GLUD1	ITPRIP	OLFML1	ELF5	EHD1
TMEM51	MATN1-AS1	SLC5A9	PKN2	VANGL1	S100A6	CEP350	MIR135B	ABCB10	NEBL-AS1	LINC01553	NUTM2A	SORCS3	PPFIBP2	EHF	MAJIN
DDI2	SDC3	AGBL4	RBMXL1	MAB21L3	S100A2	QSOX1	CDK18	GALNT2	MIR1915	ANK3	NUTM2A-AS1	ADD3	CYB5R2	MIR1343	ARL2-SNX15
UQCRHL	SNORD103C	DMRTA2	LRRC8B	ATP1A1	AQP10	ACBD6	ELK4	PGBD5	MIR1915HG	RHOBTB1	PAPSS2	SMNDC1	TUB	CD44	POLA2
EPHA2	PUM1	FAF1	LRRC8C-DT	CD58	IL6R	XPR1	SLC26A9	C1orf198	MLLT10	TMEM26	KLLN	DUSP5	LMO1	SLC1A2	TIGD3
FBXO42	NKAIN1	CDKN2C	LRRC8C	MIR320B1	SHE	IER5	FAM72A	FAM89A	DNAJC1	CABCOCO1	PTEN	RBM20	TRIM66	PAMR1	SLC25A45
CROCCP3	SERINC2	OSBPL9	LRRC8D	TRIM45	ZBTB7B	LINC01699	SRGAP2	TRIM67	LOC100130992	ARID5B	RNLS	PDCD4-AS1	ST5	FJX1	NEAT1
MIR3675	LINC01226	ZFYVE9	GEMIN8P4	GDAP2	EFNA1	ZNF648	SRGAP2C	EGLN1	SPAG6	MIR548AV	FAS-AS1	SHOC2	IPO7	MIR3973	MALAT1
NBPF1	TINAGL1	TUT4	ZNF326	WARS2	GBA	RGSL1	MAPKAPK2	DISC2	PIP4K2A	ZNF365	MIR4679-1	GPAM	ZNF143	LDLRAD3	KAT5
CROCCP2	COL16A1	COA7	BARHL2	HAO2	CLK2	SHCBP1L	C1orf116	LINC01354	ARMC3	ADO	LIPA	ACSL5	WEE1	PRR5L	AP5B1
CROCC	ADGRB2	ECHDC2	HFM1	ZNF697	GON4L	LAMC1	YOD1	IRF2BP2	ARHGAP21	JMJD1C	IFIT2	MIR4295	SWAP70	TRAF6	FOSL1
MST1L	KPNA6	PODN	TGFBR3	NOTCH2	LMNA	NMNAT2	CD55	LINC00184	PRTFDC1	JMJD1C-AS1	IFIT1B	TCF7L2	LOC440028	API5	C11orf68
PADI2	MTMR9LP	SLC1A7	EPHX4	FAM72B	SMG5	SMG7-AS1	CD34	LINC01132	ENKUR	REEP3	SLC16A12	CASP7	SBF2	ALKBH3	DRAP1
PADI1	ZBTB8A	LRRC42	GFI1	SRGAP2D	NES	NCF2	PLXNA2	SNORA14B	GPR158	ANXA2P3	LINC00865	VWA2	ADM	ALKBH3-AS1	YIF1A
ARHGEF10L	ZBTB8B	CDCP2	DIPK1A	EMBP1	PEAR1	COLGALT2	CAMK1G	TBCE	ABI1	HERC4	HTR7	AFAP1L2	AMPD3	C11orf96	RIN1
ACTL8	KIAA1522	MRPL37	MTF2	ANKRD20A12P	KIRREL1	RNF2	TRAF3IP3	LYST	LYZL1	MYPN	ANKRD1	ABLIM1	MTRNR2L8	ACCS	NPAS4
PAX7	YARS	SSBP3	CCDC18-AS1	NA	CADM3	HMCN1	C1orf74	LGALS8	PTCHD3P1	KIF1BP	LINC00502	SHTN1	MRVI1	CD82	PC
MIR4695	HPCA	ACOT11	FNBP1L	FAM72D	TAGLN2	RGS1	IRF6	RPS7P5	MIR938	HKDC1	PCGF5	VAX1	ZBED5-AS1	TSPAN18	C11orf86
IFFO2	RNF19B	DHCR24	BCAR3	NBPF20	LINC01133	RGS2	UTP25	RGS7	SVIL	HK1	HECTD2	SLC18A2	CSNK2A3	LINC02685	POLD4
PLA2G2E	ZSCAN20	LOC100507634	MIR760	NBPF9	ATP1A2	GLRX2	SYT14	FH	LYZL2	TSPAN15	PPP1R3C	PDZD8	GALNT18	PRDM11	CLCF1
UBXN10	C1orf94	MIR4422	GCLM	PDE4DIP	PEA15	CDC73	SERTAD4	OPN3	ARHGAP12	COL13A1	TNKS2	EMX2OS	USP47	SYT13	GSTP1
VWA5B1	GJB4	PRKAA2	ABCA4	LOC653513	DCAF8	B3GALT2	HHAT	PLD5	KIF5B	H2AFY2	FGFBP3	PRLHR	DKK3	LOC100507384	UNC93B1
LINC01141	SMIM12	OMA1	ARHGAP29	SEC22B	F11R	DENND1B	KCNH1	LINC01347	EPC1	TYSND1	BTAF1	NANOS1	MICAL2	CREB3L1	ALDH3B1
CAMK2N1	DLGAP3	MYSM1	F3	NBPF10	USF1	LHX9	RCOR3	AKT3	CCDC7	NPFFR1	IDE	PRDX3	TEAD1	CHRM4	NDUFS8

CHKA	MIR4693	ARHGAP32	KRAS	C12orf80	RASSF9	MIR620	STARD13	DOCK9	FSCB	PLEKHH1	DYNC1H1	EIF3J-DT	PIAS1	ASB9P1	HS3ST2
MRGPRF	MSANTD4	TMEM45B	LMNTD1	KRT7	MGAT4C	MED13L	RFC3	UBAC2	C14orf28	PIGH	RCOR1	TRIM69	CORO2B	CHD2	USP31
TPCN2	AASDHPPT	APLP2	RASSF8-AS1	KRT86	C12orf29	MIR4472-2	MIR548F5	PCCA	RPS29	RAD51B	TRAF3	SHF	DRAIC	NR2F2	GGA2
MYEOV	GUCY1A2	C11orf44	RASSF8	KRT81	KITLG	LINC00173	CCNA1	PCCA-AS1	DNAAF2	ZFP36L1	KLC1	SLC28A2	LINC00593	MIR4714	CACNG3
CCND1	CWF19L2	OPCML	BHLHE41	KRT75	LINC02458	HRK	CSNK1A1L	GGACT	KLHDC1	ACTN1-AS1	AHNAK2	SLC30A4	TLE3	IGF1R	RBBP6
ANO1	ELMOD1	LOC283177	SSPN	KRT6A	DUSP6	NOS1	POSTN	TMTC4	NEMF	DCAF5	GPR132	BLOC1S6	UACA	SYNM	TNRC6A
FADD	SLN	WASH8P	ITPR2	KRT71	ATP2B1-AS1	KSR2	LINC00366	NALCN-AS1	ARF6	SUSD6	CRIP2	SQOR	LRRC49	LRRC28	ARHGAP17
CTTN	SLC35F2	IQSEC3	MED21	KRT1	LINC00615	WSB2	NHLRC3	NALCN	LINC01588	MAP3K9	LINC00226	MYEF2	THSD4	MEF2A	KDM8
SHANK2-AS3	RAB39A	NINJ2	STK38L	KRT76	DCN	TAOK3	LHFPL6	ITGBL1	DMAC2L	PCNX1	LINC00221	CTXN2	ADPGK	ASB7	IL4R
SHANK2	EXPH5	ERC1	PPFIBP1	KRT8	EEA1	HSPB8	LINC00332	FGF14	MAP4K5	SIPA1L1	CHEK2P2	DUT	HCN4	ALDH1A3	EIF3CL
FOLR1	DDX10	LINC00942	PTHLH	CSAD	LOC643339	CIT	LINC00598	METTL21C	ATL1	RGS6	HERC2P3	FBN1	INSYN1	CHSY1	EIF3C
RELT	ZC3H12C	ADIPOR2	ERGIC2	RARG	NUDT4	BICDL1	SLC25A15	LINC00551	SAV1	DPF3	NBEAP1	COPS2	PML	TARSL2	SULT1A1
FAM168A	RDX	DCP1B	TMTC1	AAAS	SOCS2	PXN	WBP4	FAM155A	PYGL	DNAL1	LINC01193	DTWD1	ISLR2	WASH3P	RRN3P2
RAB6A	FDX1	CACNA1C-IT3	DDX11	LOC100652999	CRADD	SIRT4	KBTBD6	MYO16-AS1	TRIM9	ELMSAN1	LOC646214	TNFAIP8L3	ISLR	HBQ1	SNX29P2
DNAJB13	C11orf53	CACNA1C	DENND5B	CALCOCO1	MIR5700	ORAI1	MTRF1	COL4A1	TMX1	ENTPD5	CXADRP2	CYP19A1	STRA6	LINC00235	SMG1P2
C2CD3	LAYN	ITFG2-AS1	DENND5B-AS1	HOXC13	KRT19P2	RHOF	MIR5006	COL4A2	FRMD6-AS2	SYNDIG1L	REREP3	DMXL2	CYP11A1	RAB40C	SLC7A5P1
P4HA3	SIK2	PRMT8	AMN1	HOXC9	NDUFA12	CLIP1	DGKH	RAB20	FRMD6-AS1	NPC2	NIPA1	SCG3	SEMA7A	METRN	ASPHD1
LIPT2	PPP2R1B	DYRK4	RESF1	MIR3198-2	VEZT	CLIP1-AS1	ENOX1	LINC00346	NID2	LTBP2	GOLGA8S	TMOD3	ARID3B	UBE2I	TMEM219
XRRA1	CRYAB	NTF3	BICD1	ITGA5	USP44	VPS37B	SMIM2-IT1	ANKRD10	PTGER2	AREL1	HERC2	MAPK6	PPCDC	SNORD60	NPIPB13
TPBGL	IL18	VWF	FGD4	BLOC1S1	PGAM1P5	ABCB9	TSC22D1	TUBGCP3	GNPNAT1	DLST	GOLGA8G	MYO5A	SIN3A	TBC1D24	CD2BP2
ARRB1	PLET1	CD9	DNM1L	CD63	NTN4	PITPNM2	LINC00330	ATP11A	DDHD1	EIF2B2	WHAMMP2	FAM214A	PTPN9	ZG16B	SEPHS2
RPS3	TTC12	PLEKHG6	PKP2	ERBB3	SNRPF	TMED2	NUFIP1	MCF2L	MIR5580	TMED10	APBA2	ONECUT1	SNX33	FLYWCH1	ZNF668
GDPD5	DRD2	TNFRSF1A	ALG10	CTDSP2	LTA4H	ZNF664	GTF2F2	PCID2	BMP4	FOS	FAM189A1	RSL24D1	SCAPER	TNFRSF12A	ITGAD
UVRAG	TMPRSS5	CD27	CPNE8	ATP23	ELK3	SCARB1	KCTD4	CUL4A	CDKN3	LINC01220	TJP1	CCPG1	ACSBG1	THOC6	ZNF267
WNT11	USP28	MLF2	KIF21A	USP15	SLC9A7P1	UBC	SNORA31	TMCO3	CGRRF1	ERG28	CHRFAM7A	PRTG	MORF4L1	TFAP4	LOC390705
EMSY	NNMT	FOXJ2	GXYLT1	MIRLET7I	IKBIP	MIR5188	SIAH3	RASA3	SAMD4A	TTLL5	MTMR10	NEDD4	RASGRF1	HMOX2	TP53TG3B
AQP11	C11orf71	MFAP5	PRICKLE1	PPM1H	SPIC	LINC02372	SUCLA2	UPF3A	SOCS4	ESRRB	OTUD7A	TCF12	ANKRD34C-AS1	UBN1	SLC6A10P
RSF1	LINC00900	RIMKLB	TMEM117	DPY19L2	DRAM1	EP400	LPAR6	OR11H12	MAPK1IP1L	VASH1	CHRNA7	MYZAP	TMED3	PPL	LINC00273
THRSP	BUD13	TMEM52B	NELL2	SRGAP1	IGF1	GALNT9	FNDC3A	BMS1P17	LGALS3	LRRC74A	ULK4P3	POLR2M	MINAR1	ABAT	UBE2MP1
NDUFC2	SIK3	GABARAPL1	ANO6	C12orf66	STAB2	ANKRD20A9P	CDADC1	BMS1P18	TBPL2	IRF2BPL	LINC02256	ALDH1A2	ZFAND6	C16orf72	TP53TG3HP
GAB2	RNF214	YBX3	PLEKHA8P1	TBK1	NT5DC3	MPHOSPH8	RCBTB1	METTL17	KTN1	TMEM63C	FMN1	LIPC	CEMIP	ATF7IP2	RNA5SP411
RAB30	BACE1-AS	ETV6	LINC00938	RASSF3	TTC41P	PSPC1	SPRYD7	ARHGEF40	LINC00520	POMT2	RYR3	ADAM10	IL16	NUBP1	LINC02167
ANKRD42	CD3E	BCL2L14	ARID2	MSRB3	TXNRD1	ZMYM5	DLEU2	ZNF219	PELI2	SPTLC2	AVEN	SLTM	STARD5	CLEC16A	ANKRD26P1
CREBZF	LOC100131626	BORCS5	SCAF11	HMGA2	MIR3922	CRYL1	ST13P4	DAD1	NAA30	NRXN3	PGBD4	LDHAL6B	MEX3B	RMI2	C16orf87
SYTL2	PHLDB1	DUSP16	SLC38A1	LLPH	WASHC4	EEF1AKMT1	WDFY2	OXA1L	SLC35F4	STON2	KATNBL1	MYO1E	CPEB1	LITAF	NETO2
EED	DDX6	GPR19	SLC38A2	TMBIM4	C12orf75	LATS2	DHRS12	AJUBA	ARMH4	KCNK10	SLC12A6	GCNT3	AP3B2	MIR4718	PHKB
CCDC81	MIR4492	APOLD1	SLC38A4	IRAK3	NUAK1	MRPL57	TMEM272	C14orf93	PSMA3	PTPN21	LPCAT4	ANXA2	WHAMM	MIR365A	ABCC12
ME3	BCL9L	DDX47	PCED1B	CAND1	TCP11L2	MICU2	NEK3	ACIN1	DACT1	EML5	ACTC1	RORA	BNC1	MIR3179-3	SIAH1
PRSS23	CCDC84	RPL13AP20	PCED1B-AS1	IFNG	POLR3B	SACS	VPS36	DHRS4	DAAM1	FOXN3-AS2	DPH6	C2CD4A	SH3GL3	MIR3180-3	CBLN1
FZD4	H2AFX	GPRC5A	HDAC7	MDM1	RFX4	ANKRD20A19P	MIR759	CARMIL3	GPR135	TTC7B	C15orf41	C2CD4B	ADAMTSL3	PDXDC1	ZNF423
FZD4-DT	USP2	GPRC5D-AS1	ADCY6	RAP1B	MTERF2	SPATA13	OR7E156P	TM9SF1	L3HYPDH	RIN3	FAM98B	TLN2	SLC28A1	MARF1	TENT4B
GRM5-AS1	NECTIN1	HEBP1	CACNB3	MDM2	CRY1	PARP4	PCDH9-AS2	PRKD1	JKAMP	LGMN	RASGRP1	MIR190A	PDE8A	FOPNL	TOX3
SLC36A4	TRIM29	HTR7P1	RND1	CPM	BTBD11	TPTE2P1	PCDH9-AS3	AP4S1	PCNX4	CHGA	C15orf54	TPM1	AKAP13	ABCC1	FTO
VSTM5	OAF	GSG1	PRKAG1	CPSF6	CMKLR1	MTMR6	PCDH9	HECTD1	PRKCH	ITPK1-AS1	THBS1	RPS27L	KLHL25	MIR3179-2	IRX3
HEPHL1	TMEM136	EMP1	KMT2D	YEATS4	CORO1C	CDK8	DACH1	HEATR5A	FLJ22447	ASB2	SRP14-AS1	RAB8B	AGBL1-AS1	NOMO2	IRX5
PANX1	ARHGEF12	LINC01559	TUBA1A	RAB3IP	SSH1	USP12	KLF5	DTD2	RHOJ	SERPINA6	PAK6	APH1B	LINC00052	ABCC6P1	LPCAT2
IZUMO1R	GRIK4	GRIN2B	TUBA1C	CNOT2	SVOP	RASL11A	LMO7	ARHGAP5	WDR89	SERPINA9	CCDC9B	USP3	NTRK3	ARL6IP1	NUP93
AMOTL1	TBCEL	ATF7IP	KCNH3	PTPRB	TRPV4	LNX2	SCEL	AKAP6	SYNE2	SERPINA5	DISP2	DAPK2	MIR1179	TMC7	NLRC5
ENDOD1	SC5D	ART4	FMNL3	PTPRR	TCHP	POLR1D	RNF219	EAPP	ZBTB25	SERPINA3	KNSTRN	SNX1	AEN	ITPRIPL2	CPNE2
SESN3	UBASH3B	PTPRO	BCDIN3D	TMEM19	C12orf76	GSX1	NDFIP2-AS1	CFL2	PPP1R36	CLMN	C15orf62	CSNK1G1	ISG20	GDE1	RSPRY1
FAM76B	BSX	EPS8	ASIC1	TPH2	PPP1CC	MTUS2	NDFIP2	FAM177A1	PLEKHG3	SYNE3	ZFYVE19	ZNF609	HAPLN3	CRYM	ARL2BP
MIR1260B	HSPA8	DERA	LIMA1	KRR1	CCDC63	SLC7A1	LINC00353	NFKBIA	RAB15	VRK1	DLL4	MTFMT	MFGE8	CRYM-AS1	KIFC3
MAML2	SCN3B	SKP1P2	MIR1293	PHLDA1	MYL2	LINC00544	MIR622	LINC00609	MIR4708	C14orf177	MGA	PARP16	ABHD2	NPIPB3	GINS3
ARHGAP42	ROBO3	MIR3974	DIP2B	NAP1L1	LINC01405	KATNAL1	LINC00410	SFTA3	FUT8-AS1	BCL11B	MAPKBP1	INTS14	RLBP1	SMG1P3	NDRG4
CEP126	ROBO4	RERGL	ATF1	ZDHHC17	OAS3	LINC00426	ABCC4	MIR4503	FUT8	CCDC85C	SPTBN5	MIR4311	MIR9-3	SLC7A5P2	CDH5
YAP1	PKNOX2	PLEKHA5	SLC11A2	MIR1252	TPCN1	USPL1	CLDN10-AS1	SLC25A21-AS1	CCDC196	EML1	EHD4	SNAPC5	RHCG	RRN3P1	BEAN1
BIRC2	PATE2	AEBP2	BIN2	PAWR	PLBD2	ALOX5AP	DNAJC3	LINC00639	GPHN	EVL	TP53BP1	SMAD6	AP3S2	MOSMO	TK2
TMEM123	CDON	PDE3A	GALNT6	PPP1R12A	LHX5	TEX26	UGGT2	SEC23A	FAM71D	DEGS2	PDIA3	SMAD3	IQGAP1	EEF2K	CMTM3
MMP7	ST3GAL4	PYROXD1	NR4A1	OTOGL	RBM19	HSPH1	IPO5	MIA2	MPP5	SLC25A29	FRMD5	C15orf61	ST8SIA2	NPIPB5	CA7
MMP20	ETS1	ST8SIA1	KRT80	PTPRQ	TBX3	FRY	STK24	FBXO33	EIF2S1	WDR25	CASC4	SKOR1	FAM174B	OTOAP1	CBFB

PLEKHG4	PAFAH1B1	NUFIP2	GJC1	AXIN2	DLGAP1	ONECUT2	02-Mar	C19orf33	SNAR-A3	YPEL5	RTN4	RMND5A	CCDC93	BBS5	CFLAR
KCTD19	MIR1253	MIR4523	NMT1	CEP112	DLGAP1-AS4	ATP8B1	MUC16	ACTN4	MYH14	LBH	MTIF2	RNF103-CHMP3	INSIG2	CCDC173	CFLAR-AS1
CTCF	RAP1GAP2	ANKRD13B	PLCD3	PRKCA	PTPRM	NEDD4L	OR1M1	ACP7	VSIG10L	LCLAT1	CCDC88A	LOC285074	EN1	KLHL23	STRADB
CENPT	OR1D5	CORO6	ACBD4	CACNG5	MTCL1	ALPK2	ZNF121	PLEKHG2	NLRP12	CAPN13	PPP4R3B	CYTOR	MARCO	METTL5	CDK15
SLC12A4	SPNS2	SSH2	HEXIM1	CACNG4	TXNDC2	MALT1	C3P1	RPS16	MYADM	EHD3	EFEMP1	MIR4435-1	STEAP3	SP5	SNORD11
NFATC3	GLTPD2	BLMH	MAP3K14	HELZ	GNAL	ZNF532	PDE4A	EID2	MBOAT7	SLC30A6	VRK2	THNSL2	SCTR	METTL8	BMPR2
SMPD3	MINK1	TBC1D29P	ARHGAP27	PITPNC1	MPPE1	SEC11C	LDLR	PSMC4	SYT5	LINC00486	REL	FOXI3	TMEM177	CYBRD1	ICA1L
CDH1	RNF167	CRLF3	PLEKHM1	C17orf58	IMPA2	CCBE1	SPC24	SERTAD1	PTPRH	LTBP1	USP34	TEX37	PTPN4	DYNC1I2	ABI2
TANGO6	LOC728392	TEFM	MAPK8IP1P2	KPNA2	ANKRD62	PMAIP1	TMEM205	SPTBN4	PPP6R1	RASGRP3	XPO1	EIF2AK3	INHBB	METAP1D	RAPH1
MIR1538	PITPNM3	MIR4733	LINC02210	LINC00674	TUBB6	RNF152	ECSIT	LTBP4	KMT5C	CRIM1	FAM161A	MIR4436A	GLI2	DLX2	PARD3B
PDXDC2P-NPIPB14	FBXO39	SUZ12	STH	ARHGAP27P2	AFG3L2	ZCCHC2	ZNF440	NUMBL	SMIM17	FEZ2	MIR5192	LSP1P4	TFCP2L1	ITGA6	NRP2
CLEC18C	TEKT1	C17orf75	KANSL1	SOX9	SPIRE1	PHLPP1	ZNF433	COQ8B	TPO	VIT	TMEM17	GGT8P	CLASP1	RAPGEF4	INO80D
VAC14	GPS2	CCL2	KANSL1-AS1	LINC00673	FAM210A	SERPINB5	ZNF442	CCDC97	RNF144A	EIF2AK2	EHBP1	ACTR3BP2	TSN	MAP3K20	GPR1
IST1	TMEM256-PLSCR3	RFFL	LRRC37A	CDC42EP4	ANKRD20A5P	SERPINB7	IER2	TGFB1	LINC00298	CDC42EP3	UGP2	KCNIP3	BIN1	CDCA7	KLF7
PMFBP1	POLR2A	SLFN5	LRRC37A2	BTBD17	ROCK1	DSEL	CACNA1A	ARHGEF1	LINC00299	RMDN2	PELI1	FAHD2A	PROC	WIPF1	CREB1
ZFHX3	RPL29P2	MMP28	NSF	RAB37	GATA6-AS1	DOK6	CCDC130	LIPE	ID2	ATL2	LINC00309	TRIM43	IWS1	CHN1	METTL21A
HCCAT5	KDM6B	CCL4	RPRML	SLC9A3R1	CTAGE1	CD226	LOC284454	PSG3	MBOAT2	GALM	LGALSL	FAHD2CP	AMMECR1L	ATP5MC3	CCNYL1
LOC283922	PER1	TBC1D3B	EFCAB13	CDR2L	RBBP8	SOCS6	MIR181C	PSG8	ASAP2	SRSF7	AFTPH	DUSP2	SAP130	HNRNPA3	FZD5
CLEC18B	NDEL1	TBC1D3C	MRPL45P2	SLC16A5	CABLES1	LINC01541	NANOS3	PSG8-AS1	YWHAQ	GEMIN6	LINC02579	ARID5A	HS6ST1	MIR3128	PLEKHM3
GLG1	STX8	TADA2A	MIR1203	NT5C	TMEM241	ZNF407	IL27RA	PSG10P	GRHL1	DHX57	SERTAD2	KANSL3	RAB6C	NFE2L2	PIKFYVE
FA2H	USP43	DUSP14	CALCOCO2	MIR3678	LAMA3	ZADH2	LOC100507373	PSG1	RRM2	SOS1	LINC02245	CNNM4	MED15P9	AGPS	MAP2
ZNRF1	GAS7	HNF1B	UBE2Z	CASKIN2	TTC39C	LOC339298	DNAJB1	PSG11	ATP6V1C2	MAP4K3	SLC1A4	ANKRD23	AMER3	PDE11A	UNC80
LDHD	ZNF18	LOC440434	GIP	ITGB4	OSBPL1A	ATP9B	AP1M1	PSG2	KCNF1	MAP4K3-DT	SPRED2	TMEM131	POTEE	RBM45	ACADL
BCAR1	MAP2K4	MRPL45	IGF2BP1	UNC13D	LINC01915	TXNL4A	KLF2	PSG5	PQLC3	TMEM178A	ETAA1	KIAA1211L	MZT2A	OSBPL6	CPS1
TMEM170A	MYOCD	ARHGAP23	GNGT2	WBP2	SS18	WASH5P	MYO9B	PSG6	ROCK2	THUMPD2	C1D	REV1	MIR663B	SESTD1	CPS1-IT1
CHST5	ELAC2	ARL5C	NGFR	TRIM47	KCTD1	PTBP1	OCEL1	PSG9	E2F6	EML4	APLF	LONRF2	ANKRD30BL	CWC22	MIR548F2
SYCE1L	TEKT3	STAC2	ITGA3	TRIM65	GAREM1	GPX4	UNC13A	PRG1	LPIN1	COX7A2L	GKN2	NMS	GPR39	UBE2E3	ERBB4
ADAMTS18	CDRT4	NEUROD2	SPATA20	MRPL38	MAPRE2	MIDN	ARRDC2	SMG9	MIR4262	KCNG3	GKN1	PDCL3	LYPD1	MIR4437	MIR4776-1
CLEC3A	TRIM16	ERBB2	ABCC3	SPHK1	ZNF396	CIRBP	PDE4C	KCNN4	NBAS	MTA3	ANTXR1	NPAS2	MIR3679	ITPRID2	VWC2L
CENPN	ADORA2B	RARA	ANKRD40	SNHG16	INO80C	ADAMTSL5	MIR3188	ZNF227	DDX1	HAAO	GFPT1	TBC1D8	R3HDM1	NUP35	ATIC
MIR4720	TTC19	IGFBP4	TOB1	SNORD1A	GALNT1	UQCR11	GDF15	ZNF235	MYCN	ZFP36L2	SNORA36C	RNF149	MIR128-1	ZC3H15	04-Mar
PLCG2	MIR1288	TNS4	SPAG9	ST6GALNAC2	C18orf21	BTBD2	LRRC25	IGSF23	FAM49A	LINC01126	AAK1	CREG2	UBXN4	ITGAV	IGFBP5
HSBP1	ZNF287	SMARCE1	UTP18	SNHG20	SLC39A6	MKNK2	REX1BD	BCL3	LINC00954	THADA	ANXA4	MAP4K4	CXCR4	FAM171B	DIRC3
NECAB2	MPRIP	KRT10	HLF	SEC14L1	FHOD3	DOT1L	TMEM59L	CBLC	SDC1	DYNC2LI1	GMCL1	SLC9A4	GTDC1	MIR3129	TMBIM1
SLC38A8	TOM1L2	KRT20	MMD	SEPTIN9	TPGS2	SPPL2B	CRTC1	PPP1R13L	PUM2	SLC3A1	LINC01816	FHL2	ZEB2	COL5A2	TUBA4B
COTL1	DRC3	KRT23	TMEM100	TNRC6C	CELF4	LMNB2	COMP	ERCC1	HS1BP3	SIX3	SNRPG	NCK2	RND3	WDR75	DNAJB2
USP10	SLC5A10	KRT34	PCTP	LINC01993	SLC14A2	GADD45B	HOMER3	FOSB	APOB	SIX2	FAM136A	UXS1	RBM43	SLC40A1	DNPEP
ZDHHC7	GRAP	LOC100505782	ANKFN1	SOCS3	SLC14A1	GNG7	GATAD2A	RTN2	KLHL29	LINC01121	TGFA	LIMS1	ARL5A	ANKAR	SPEG
C16orf74	GRAPL	KRT32	NOG	CYTH1	SIGLEC15	SLC39A3	ZNF737	VASP	ATAD2B	PRKCE	ADD2	MIR4265	STAM2	INPP1	TMEM198
IRF8	EPN2	KRT13	TRIM25	TIMP2	PSTPIP2	ZNF77	ZNF675	EML2	MFSD2B	EPAS1	VAX2	MIR4266	FMNL2	MFSD6	MIR4268
LINC02135	SLC47A2	KRT15	CUEDC1	LGALS3BP	C18orf25	S1PR4	HAVCR1P1	FBXO46	EFR3B	RHOQ	DYSF	SEPTIN10	NR4A2	NEMP2	ACSL3
FOXL1	AKAP10	KRT19	RNF43	ENGASE	LOXHD1	SMIM24	LINC00662	NOVA2	POMC	CRIPT	CYP26B1	LIMS3-LOC440895	GPD2	NAB1	SCG2
KLHDC4	SPECC1	KRT16	TRIM37	RBFOX3	PIAS2	ATCAY	UQCRFS1	IGFL4	DNMT3A	SOCS5	EXOC6B	MIR4436B1	ACVR1	GLS	AP1S3
SLC7A5	MAP2K3	KRT42P	CLTC	RNF213	SKOR2	DAPK3	PLEKHF1	LOC93429	DTNB	LINC01118	SPR	MALL	UPP2	NABP1	WDFY1
BANP	KCNJ12	GAST	VMP1	BAIAP2	SMAD2	MAP2K2	CCNE1	IGFL1	KIF3C	LINC01119	SFXN5	MTLN	PKP4	CAVIN2	MRPL44
ZNF469	FLJ36000	JUP	MIR21	BAHCC1	ZBTB7C	CREB3L3	URI1	HIF3A	GAREM2	MCFD2	RAB11FIP5	LINC01106	DAPL1	SLC39A10	FAM124B
SNAI3	MTRNR2L1	STAT5B	TBC1D3P1-DHX40P1	NPLOC4	CTIF	SIRT6	ZNF507	AP2S1	KCNK3	TTC7A	STAMBP	BCL2L11	BAZ2B	STK17B	DOCK10
PIEZO1	MIR4522	STAT5A	BCAS3	SECTM1	MIR4743	SH3GL1	RHPN2	ARHGAP35	MAPRE3	STPG4	MTHFD2	MIR4435-2HG	LY75	HECW2	MIR548AR
SPATA2L	WSB1	STAT3	C17orf82	HEXD	SMAD7	DPP9	GPATCH1	NPAS1	PREB	CALM2	SEMA4F	ANAPC1	PLA2R1	GTF3C3	LOC646736
SPIRE2	TBC1D3P5	CAVIN1	MED13	OGFOD3	MIR4744	MIR7-3HG	LRP3	NOP53	TRIM54	KCNK12	TACR1	ZC3H8	ITGB6	PGAP1	NGEF
TUBB3	KSR1	ATP6V0A1	MRC2	ROCK1P1	LIPG	ZNRF4	CEBPG	SNAR-A1	MPV17	MSH6	EVA1A	TTL	MIR4785	COQ10B	TRPM8
NXN	LYRM9	PLEKHH3	10-Mar	THOC1	ACAA2	RFX2	KIAA0355	PLA2G4C	EIF2B4	FBXO11	GCFC2	FLJ42351	RBMS1	MOB4	SPP2
ABR	TMEM97	LINC00910	TANC2	TYMS	MYO5B	TNFSF9	WTIP	EMP3	SUPT7L	FOXN2	TMSB10	IL1A	TANK	SATB2	SH3BP4
MYO1C	SARM1	ETV4	SMARCD2	ADCYAP1	ME2	FLJ25758	ZNF30	KDELR1	MRPL33	PPP1R21	KCMF1	IL1B	PSMD14	TYW5	TWIST2
SLC43A2	FAM222B	MEOX1	TEX2	NDC80	SMAD4	INSR	CD22	CYTH2	LOC100505716	STON1-GTF2A1L	TCF7L1	PAX8-AS1	GRB14	SPATS2L	LINC01881
MIR132	DHRS13	SOST	SMURF2	LPIN2	DCC	ARHGEF18	CAPNS1	SULT2B1	FOSL2	PSME4	SH2D6	PAX8	COBLL1	KCTD18	SRXN1
MIR212	SEZ6	CFAP97D1	PLEKHM1P1	MYL12A	SNORA37	STXBP2	ZNF529	FTL	PLB1	ACYP2	PARTICL	WASH2P	TTC21B	AOX1	FKBP1A
SMG6	PIPOX	C17orf53	LRRC37A3	MYL12B	TXNL1	CTXN1	SIPA1L3	PPFIA3	TOGARAM2	C2orf73	ATOH8	DDX11L2	CERS6	AOX2P	NSFL1C
MNT	TIAF1	RUNDC3A	AMZ2P1	TGIF1	LINC-ROR	CD320	PPP1R14A	RRAS	CLIP4	SPTBN1	ST3GAL5	SLC35F5	ABCB11	BZW1	LOC100289473
METTL16	CRYBA1	FAM171A2	RGS9	DLGAP1-AS1	ST8SIA3	ANGPTL4	SPINT2	SIGLEC16	ALK	EML6	KDM3A	ACTR3	DHRS9	ORC2	SIRPA

PDYN	SALL4	DOP1B	TTC28-AS1	TBC1D22A	TRIM71	FAM107A	MYLK	GPR87	RTP4	SLC2A9	THEGL	AP1AR	RAPGEF2	C	TNPO1
ZNF343	TSHZ2	CLDN14	TTC28	LINC00898	CRTAP	PTPRG	KALRN	MBNL1	LPP	WDR1	HOPX	TIFA	ANP32C	LINC02120	FCHO2
CDC25B	ZNF217	HLCS	CCDC117	ZBED4	FBXL2	C3orf14	ITGB5	MBNL1-AS1	TPRG1	RAB28	REST	NEUROG2	01-Mar	TARS	TMEM174
AP5S1	SUMO1P1	RIPPLY3	ZNRF3-AS1	MAPK11	UBP1	MAGI1	HEG1	RAP2B	CLDN1	LINC01085	IGFBP7-AS1	LARP7	SMIM31	RXFP3	ARHGEF28
SMOX	BCAS1	PIGP	RHBDD3	PLXNB2	PDCD6IP	SLC25A26	MIR548I1	C3orf79	IL1RAP	CPEB2-DT	EPHA5-AS1	MIR302B	KLHL2	AMACR	HEXB
LINC01433	MIR4756	DYRK1A	NF2	MAPK8IP2	TRANK1	LRIG1	TXNRD3	ARHGEF26	OSTN	CPEB2	CENPC	ANK2	CPE	C1QTNF3	NSA2
ADRA1D	CYP24A1	ERG	CABP7	RPL23AP82	EPM2AIP1	MIR4272	PLXNA1	PLCH1	CCDC50	C1QTNF7	YTHDC1	CAMK2D	PALLD	RAI14	FAM169A
PRNP	FAM209B	LINC00114	ASCC2	SUMF1	MLH1	SUCLG2	PODXL2	SSR3	MB21D2	FGFBP1	UTP3	ARSJ	SH3RF1	TTC23L	GCNT4
SLC23A2	TFAP2C	PSMG1	MTMR3	EGOT	LRRFIP2	EOGT	ABTB1	TIPARP	DPPA2P3	TAPT1-AS1	RUFY3	METTL14	CLCN3	DNAJC21	HMGCR
GPCPD1	BMP7	C2CD2	LIF	ITPR1	GOLGA4	ARL6IP5	MGLL	LINC00880	HES1	CLRN2	CXCL8	MYOZ2	HPF1	UGT3A1	POLK
CHGB	RAE1	UMODL1-AS1	MTFP1	BHLHE40-AS1	ITGA9	LINC00870	CNBP	CCNL1	LINC00884	SLIT2	MTHFD2L	USP53	LINC02275	NADK2	F2R
PLCB4	RBM38	LINC00313	OSBP2	BHLHE40	MIR26A1	RYBP	COPG1	RSRC1	TMEM44-AS1	ADGRA3	AREG	PDE5A	AADAT	NIPBL	F2RL1
ANKEF1	PMEPA1	HSF2BP	SMTN	EDEM1	SLC22A13	SHQ1	SNORA7B	MLF1	FAM43A	DHX15	BTC	ANXA5	GALNT7	CPLANE1	AGGF1
SNAP25-AS1	RAB22A	PDXK	LIMK2	LMCD1-AS1	XYLB	GXYLT2	MBD4	IQCJ-SCHIP1	LINC01968	PI4K2B	SCARB2	IL21	WDR17	EGFLAM	ZBED3-AS1
SLX4IP	STX16	TRAPPC10	PISD	SSUH2	TTC21A	EBLN2	IFT122	SCHIP1	XXYLT1	SLC34A2	STBD1	FGF2	ASB5	PTGER4	TBCA
JAG1	PRELID3B	PTTG1IP	DEPDC5	OXTR	CSRNP1	GBE1	PLXND1	IL12A	PPP1R2	SEL1L3	SHROOM3	NUDT6	SPCS3	FBXO4	SCAMP1
LOC339593	HRH3	PICSAR	YWHAH	RAD18	MOBP	LINC00971	TMCC1	KPNA4	SDHAP2	SMIM20	MIR4450	SPRY1	DCTD	GHR	LHFPL2
BTBD3	LAMA5	POFUT2	AP1B1P1	THUMPD3	ZNF620	POU1F1	ATP2C1	ARL14	SDHAP1	RBPJ	SEPTIN11	LINC01091	WWC2-AS2	LOC648987	JMY
BFSP1	CABLES2	COL18A1	SLC5A4	SETD5	CTNNB1	EPHA3	NEK11	PPM1L	TFRC	CCKAR	CNOT6L	ANKRD50	WWC2	LOC100132356	SERINC5
OVOL2	COL9A3	COL18A1-AS1	SYN3	CAMK1	ULK4	PROS1	CPNE4	SERPINI1	PCYT1A	TBC1D19	MRPL1	SLC25A31	CLDN24	CCL28	ZFYVE16
ZNF133	DIDO1	SPATC1L	TIMP3	CIDEC	TRAK1	DHFR2	ACPP	GOLIM4	PAK2	STIM2	ANXA3	LARP1B	CDKN2AIP	PAIP1	MTRNR2L2
DZANK1	EEF1A2	YBEY	HMOX1	JAGN1	ACKR2	LINC00879	NPHP3-AS1	EGFEM1P	SENP5	ARAP2	HNRNPD	PGRMC2	LOC389247	NNT-AS1	RASGRF2
DTD1	LKAAEAR1	DIP2A	MB	EMC3	POMGNT2	EPHA6	BFSP2	MIR551B	MELTF	RELL1	SCD5	JADE1	IRF2	HCN1	SSBP2
FOXA2	NPBWR2	PRMT2	APOL6	IRAK2	ABHD5	ARL6	CDV3	MECOM	DLG1	TBC1D1	SEC31A	SCLT1	CASP3	EMB	ATP6AP1L
CST3	LINC00266-1	CCT8L2	RBFOX2	LINC00606	TOPAZ1	CRYBG3	TOPBP1	ACTRT3	BDH1	KLF3	COQ2	PABPC4L	SLC25A4	PARP8	MIR3977
SYNDIG1	MIR3648-1	HSFY1P1	MYH9	TIMP4	TGM4	CLDND1	SLCO2A1	SLC7A14	ANKRD18DP	KLF3-AS1	HPSE	PCDH18	CFAP97	ISL1	SCARNA18
APMAP	TPTE	TMEM121B	TXN2	TSEN2	TMEM158	CPOX	AMOTL2	RPL22L1	ZNF595	TLR6	GPAT3	SLC7A11	SNX25	PELO	EDIL3
LOC284798	BAGE3	CECR2	CACNG2	MKRN2	LARS2-AS1	DCBLD2	PPP2R3A	EIF5A2	PCGF3	FAM114A1	WDFY3	LINC00499	SORBS2	ARL15	MEF2C
ENTPD6	ANKRD30BP2	MIR648	C1QTNF6	NUP210	LIMD1	COL8A1	MSL2	MIR569	CPLX1	MIR574	MIR4451	NOCT	FAT1	HSPB3	ARRDC3-AS1
PYGB	ANKRD20A11P	MICAL3	CARD10	FBLN2	FYCO1	FILIP1L	STAG1	PLD1	FGFRL1	TMEM156	MAPK10	ELF2	TRIML1	SNX18	NR2F1-AS1
NANP	SAMSN1	LINC01634	TRIOBP	LINC00620	XCR1	MIR548G	SLC35G2	FNDC3B	LOC100130872	KLHL5	SLC10A6	SETD7	FRG1	ESM1	NR2F1
MIR663A	NRIP1	USP18	MICALL1	WNT7A	ALS2CL	TBC1D23	IL20RB	NCEH1	NSD2	UGDH	C4orf36	MGST2	DUX4L8	MIR5687	FAM172A
MIR663AHG	USP25	GGT3P	MAFF	TMEM43	CCDC12	ADGRG7	CLDN18	ECT2	NELFA	UBE2K	AFF1	MAML3	PLEKHG4B	RNF138P1	KIAA0825
FRG1BP	MIR99AHG	DGCR6	GTPBP1	XPC	SETD2	NFKBIZ	ARMC8	LINC00578	HGFAC	PDS5A	KLHL8	TBC1D9	SDHA	PLPP1	SLF1
DEFB115	MIR99A	TXNRD2	SUN2	LSM3	KIF9-AS1	MIR548A3	MRAS	PIK3CA	ADRA2C	N4BP2	NUDT9	RNF150	PP7080	ANKRD55	ARSK
HM13-AS1	MIRLET7C	ARVCF	APOBEC3A_B	SLC6A6	SCAP	ALCAM	PIK3CB	ZNF639	FAM86EP	LOC344967	HERC6	INPP4B	LPCAT1	MAP3K1	RFESD
ID1	MIR125B2	MED15	APOBEC3B	SH3BP5	MAP4	CBLB	FOXL2	GNB4	TMEM128	CHRNA9	HERC3	GAB1	SDHAP3	ACTBL2	RHOBTB3
MIR3193	CXADR	SERPIND1	CBX7	METTL6	PLXNB1	MBD4	MRPS22	USP13	NSG1	APBB2	FAM13A	FREM3	TENT4A	PLK2	LINC01554
BCL2L1	BTG3	TUBA3FP	PDGFB	BTD	P4HTM	LINC00636	RBP1	PEX5L	STX18-AS1	LIMCH1	TIGD2	HHIP-AS1	ROPN1L	PDE4D	ELL2
FOXS1	D21S2088E	POM121L8P	SNORD43	ANKRD28	CCDC36	CD47	PXYLP1	CCDC39	CYTL1	ATP10D	SNCA	SMAD1	ANKRD33B	NDUFAF2	PCSK1
XKR7	APP	RIMBP3C	RPS19BP1	GALNT15	DAG1	IFT57	ZBTB38	SOX2-OT	STK32B	TXK	ATOH1	MMAA	DAP	ZSWIM6	CAST
KIF3B	CYYR1	PPM1F	MRTFA	MIR3714	BSN	PHLDB2	RASA2	SOX2	LINC01587	TEC	PDLIM5	C4orf51	DNAH5	KIF2A	ERAP1
DNMT3B	N6AMT1	TOP3B	MCHR1	TBC1D5	MST1R	C3orf52	ATP1B3	LINC01994	EVC2	SLAIN2	BMPR1B	ZNF827	TRIO	IPO11	ERAP2
SNTA1	BACH1	ZNF280B	RANGAP1	SATB1	SEMA3F	CCDC80	ATR	ATP11B	EVC	FRYL	RAP1GDS1	LSM6	OTULIN	SREK1IP1	LNPEP
ZNF341	TIAM1	BCR	ZC3H7B	EFHB	GNAI2	BOC	PLS1	B3GNT5	CRMP1	OCIAD2	EIF4E	POU4F2	ANKH	ADAMTS6	LOC100289230
CHMP4B	SOD1	IGLL1	TOB2	KAT2B	SEMA3B	NAA50	SLC9A9-AS1	LINC00888	JAKMIP1	CWH43	DAPP1	ARHGAP10	11-Mar	NLN	PAM
ITCH	LINC00159	VPREB3	CSDC2	VENTXP7	TUSC2	GRAMD1C	PLSCR2	KLHL6	PPP2R2C	LRRC66	H2AFZ	LRBA	MYO10	ERBIN	PPIP5K2
DYNLRB1	MIS18A	GSTT2	SNU13	UBE2E2	MAPKAPK3	ZBTB20	GYG1	PARL	KIAA0232	USP46	FLJ20021	PRSS48	BASP1	SREK1	EFNA5
TP53INP2	EVA1C	ADORA2A	TNFRSF13C	MIR548AC	MIR4787	GAP43	CP	VWA5B2	TBC1D14	SNORA26	NFKB1	FAM160A1	LINC02111	MAST4	FER
UQCC1	TCP10L	UPB1	RNU12	UBE2E1	MANF	TUSC7	TM4SF1	CAMK2N2	SORCS2	RASL11B	MANBA	GATB	CDH18	CD180	PJA2
VSTM2L	PAXBP1-AS1	TOP1P2	ATP5MGL	RPL15	TLR9	UPK1B	TM4SF4	CHRD	MIR4798	FIP1L1	UBE2D3	FBXW7	GUSBP1	PIK3R1	MAN2A1
SLC32A1	C21orf62	TMEM211	PACSIN2	NKIRAS1	SEMA3G	B4GALT4	WWTR1	IGF2BP2	AFAP1-AS1	RPL21P44	SLC9B2	TRIM2	CDH9	OCLN	LINC01848
GTSF1L	IL10RB	KIAA1671	BIK	THRB	TKT	ARHGAP31	WWTR1-AS1	ETV5	AFAP1	CHIC2	TET2	TMEM131L	PURPL	GUSBP3	STARD4
OSER1	IFNGR2	CRYBB2P1	SULT4A1	TOP2B	DCP1A	STXBP5L	COMMD2	DGKG	ABLIM2	PDGFRA	PPA2	GRIA2	LSP1P3	SERF1A	EPB41L4A
LINC01260	TMEM50B	MYO18B	PNPLA3	NEK10	ACTR8	FAM162A	ANKUB1	TBCCD1	SH3TC1	KDR	GIMD1	GASK1B	PDZD2	GTF2H2B	APC
LINC00494	ITSN1	ASPHD2	PARVB	SLC4A7	SELENOK	KPNA1	RNF13	EIF4A2	ACOX3	SRD5A3	PAPSS1	TMEM144	MIR4279	SMA5	REEP5
PREX1	LINC00649	HPS4	PHF21B	CMC1	ARHGEF3	DTX3L	LOC646903	ADIPOQ-AS1	GPR78	PDCL2	SGMS2	C4orf46	GOLPH3	GUSBP9	DCP2
PTGIS	KCNE1	MIR548J	MIR4762	CMTM8	SPATA12	PARP15	TSC22D2	ST6GAL1	CPZ	NMU	SEC24B	FNIP2	MTMR12	MAP1B	MCC
SLC9A8	RUNX1	MIAT	LOC730668	CMTM7	SLMAP	SEC22A	SELENOT	RTP1	USP17L10	CEP135	EGF	C4orf45	ZFR	MIR4803	CCDC112
TMEM189-UBE2V	CBR3-AS1	MN1	PRR34	CMTM6	FLNB	HACD2	SIAH2	MASP1	MIR548I2	AASDH	FAM241A	MIR3688-1	SUB1	MRPS27	FEM1C

TMED7	ARHGEF37	GMDS	HIST1H2BD	C6orf223	GABRR2	MYB	FNDC1	OSBPL3	MLXIPL	SYPL1	ZYX	DOCK5	VXN	YWHAZ	CDC37L1
AP3S1	TIGD6	LINC01600	HIST1H3E	TMEM151B	UBE2J1	AHI1	SOD2	HOXA1	STX1A	NAMPT	ZNF786	EBF2	SGK3	ZNF706	RCL1
HSD17B4	PDGFRB	SERPINB9P1	HIST1H2BI	MIR4642	RRAGD	LINC00271	IGF2R	HOXA5	ABHD11	CCDC71L	ZNF282	PPP2R2A	PPP1R42	GRHL2	JAK2
SRFBP1	ARSI	SERPINB6	HCG11	SUPT3H	BACH2	MAP7	MAP3K4	HIBADH	CLDN4	PIK3CG	ZNF777	BNIP3L	CPA6	NCALD	PDCD1LG2
SNX24	RPS14	RIPK1	LINC00240	RUNX2	MIR4464	MAP3K5	AGPAT4	JAZF1	TMEM270	PRKAR2B	ZNF746	PNMA2	PREX2	RRM2B	ERMP1
PPIC	NDST1	BPHL	ZNF391	CLIC5	FBXL4	IFNGR1	PACRG	CHN2	ELN	HBP1	ATP6V0E2-AS1	DPYSL2	C8orf34	ODF1	IL33
CEP120	SYNPO	TUBB2A	HIST1H2BL	PLA2G7	FAXC	TNFAIP3	PACRG-AS1	PRR15	LIMK1	COG5	LRRC61	STMN4	LINC01592	KLF10	UHRF2
CSNK1G3	SMIM3	PXDC1	ZKSCAN8	ADGRF1	USP45	PERP	CAHM	MIR550A3	CLIP2	BCAP29	REPIN1	CLU	SULF1	AZIN1	GLDC
ZNF608	TNIP1	FAM50B	ZSCAN9	CD2AP	PRDM13	ARFGEF3	QKI	SCRN1	GTF2IRD2	LAMB1	GIMAP5	ELP3	NCOA2	BAALC	KDM4C
MEGF10	CCDC69	PRPF4B	LINC01623	OPN5	SIM1	PBOV1	RNASET2	MTURN	GTF2IP1	NRCAM	NOS3	ZNF395	TRAM1	BAALC-AS1	PTPRD
CTXN3	ATOX1	ECI2	HLA-L	MMUT	HACE1	HEBP2	TCP10L2	AQP1	CCL26	IFRD1	ASIC3	FZD3	SBSPON	RIMS2	LURAP1L
LINC01184	NMUR2	CDYL	IER3	C6orf141	LIN28B-AS1	MIR3145	LINC01558	LOC100130673	MDH2	MDFIC	PRKAG2	EXTL3	STAU2-AS1	ABRA	MPDZ
HINT1	MFAP3	PPP1R3G	DDR1	PAQR8	PREP	FLJ46906	AFDN	DPY19L1P1	SRRM3	TFEC	KMT2C	KIF13B	STAU2	NUDCD1	LINC01235
RAPGEF6	GALNT10	MIR3691	HCG22	EFHC1	PRDM1	ECT2L	SMOC2	BMPER	ZP3	TES	FABP5P3	LINC00589	UBE2W	SYBU	LINC00583
CSF2	LARP1	LYRM4	PSORS1C1	TRAM2	ATG5	REPS1	THBS2	NPSR1-AS1	RSBN1L	CAV2	ACTR3B	MIR3148	GDAP1	MED30	NFIB
SLC22A5	HAVCR1	FARS2	CDSN	GSTA3	CRYBG1	ABRACL	DLL1	HERPUD2	CD36	CAV1	DPP6	SARAF	PKIA	EXT1	TTC39B
IRF1	ADAM19	LY86	MICA	GSTA4	BEND3	HECA	FAM20C	EEPD1	GNAT3	MET	HTR5A	RBPMS	HEY1	COL14A1	BNC2
IL4	RNF145	CAGE1	C6orf47	ICK	OSTM1	CITED2	WI2-2373I1.2	KIAA0895	SEMA3C	CAPZA2	INSIG1	GTF2E2	MRPS28	MTBP	ADAMTSL1
SHROOM1	UBLCP1	DSP	NEU1	MIR5685	LINC00222	LINC01625	SUN1	AOAH-IT1	PCLO	ST7	SHH	PPP2CB	TPD52	MRPL13	HAUS6
FSTL4	LOC285626	PIP5K1P1	TNXB	GCLC	SESN1	FILNC1	GET4	ELMO1	SEMA3E	ST7-AS1	ESYT2	NRG1-IT3	ZBTB10	ZHX2	MLLT3
UBE2B	LINC01845	SCARNA27	NOTCH4	KLHL31	CD164	MIR3668	ADAP1	POU6F2-AS1	SEMA3A	ST7-AS2	OR4F21	FUT10	PAG1	DERL1	IFNA1
CDKN2AIPNL	ADRA1B	EEF1E1	TAP2	LRRC1	PPIL6	MIR4465	MICALL2	LOC646999	KIAA1324L	TSPAN12	ANGPT2	RNF122	SNX16	TBC1D31	MTAP
PCBD2	CCNG1	TFAP2A-AS1	TAP1	COL21A1	GPR6	NMBR	MAFK	LINC00265	DBF4	FAM3C	DEFB1	BRF2	CA13	KLHL38	CDKN2B-AS1
C5orf66-AS2	TENM2	MIR5689	PSMB9	DST	WASF1	ADGRG6	TFAMP1	SUGCT	SRI	WASL	DEFA6	RAB11FIP1	REXO1L1P	ANXA13	CDKN2B
TGFBI	WWC1	GCNT2	COL11A2	ZNF451	SLC22A16	LOC153910	MIR4655	MRPL32	STEAP2	SND1	DEFA5	EIF4EBP1	PSKH2	FER1L6-AS1	TEK
SMAD5	PANK3	ELOVL2	DAXX	BAG2	SLC16A10	HIVEP2	CHST12	STK17A	FZD1	SND1-IT1	FAM90A7P	PLPP5	ATP6V0D2	TMEM65	EQTN
TRPC7	SLIT3	SMIM13	LINC00336	PRIM2	TRAF3IP2	ADAT2	MIR4648	COA1	LRRD1	MIR129-1	MIR548I3	LETM2	WWP1	NSMCE2	MOB3B
SPOCK1	MIR585	ERVFRD-1	ITPR3	GUSBP4	CCN6	PHACTR2	SDK1	BLVRA	PEX1	METTL2B	FAM86B3P	C8orf86	RIPK2	TRIB1	ACO1
NPY6R	SPDL1	NEDD9	LEMD2	KHDRBS2	LAMA4	STX11	FOXK1	URGCP	FAM133B	FLNC	PRAG1	TACC1	OSGIN2	LRATD2	B4GALT1
FAM13B	DOCK2	TMEM170B	MLN	PTP4A1	RFPL4B	UTRN	AP5Z1	SPDYE1	CDK6	SMO	CLDN23	ADAM9	NBN	PCAT1	SPINK4
CTNNA1	FOXI1	ADTRP	HMGA1	ADGRB3	MARCKS	EPM2A	TNRC18	GCK	BET1	STRIP2	MFHAS1	SFRP1	DECR1	POU5F1B	NFX1
SIL1	KCNIP1	HIVEP1	ANKS1A	FAM135A	HDAC2	GRM1	FSCN1	OGDH	CASD1	UBE2H	MIR4660	ANK1	CALB1	MYC	AQP7
SPATA24	GABRP	EDN1	TCP11	OGFRL1	NT5DC1	RAB32	RNF216	SNHG15	PEG10	ZC3HC1	TNKS	KAT6A	LINC00534	MIR1204	SUGT1P1
TMEM173	FGF18	TBC1D7	ZNF76	MIR30A	DSE	STXBP5-AS1	EIF2AK1	IGFBP3	PPP1R9A	CPA2	MSRA	AP3M2	TMEM64	PVT1	ANKRD18B
PSD2	FBXW11	GFOD1	PPARD	LINC00472	RWDD1	SASH1	CYTH3	TNS3	PON2	CPA4	C8orf74	SLC20A2	SLC26A7	GSDMC	UBE2R2
PURA	SH3PXD2B	RANBP9	FKBP5	RIMS1	ROS1	TAB2	RAC1	C7orf69	MIR591	CPA1	MIR1322	CHRNB3	MIR378D2	FAM49B	UBAP1
CYSTM1	DUSP1	JARID2	LHFPL5	KCNQ5	CEP85L	LATS1	DAGLB	C7orf57	DLX6-AS1	MIR29A	MIR598	HGSNAT	PDP1	ASAP1	ENHO
PFDN1	ERGIC1	GMPR	MAPK14	MIR4282	ASF1A	RAET1E-AS1	CCZ1B	UPP1	BAIAP2L1	MKLN1	XKR6	POTEA	GEM	ADCY8	DNAJB5
HBEGF	CREBRF	ATXN1	PXT1	KCNQ5-AS1	GJA1	RAET1L	C1GALT1	ZPBP	NPTX2	PODXL	BLK	LINC00293	FSBP	EFR3A	UNC13B
ANKHD1-EIF4EBP	NKX2-5	RBM24	SRSF3	DDX43	HSF2	IYD	MIOS	DDC-AS1	KPNA7	PLXNA4	CTSB	EFCAB1	VIRMA	CCN4	RUSC2
VTRNA1-1	LOC285593	CAP2	MTCH1	EEF1A1	SMPDL3A	PLEKHG1	GLCCI1	COBL	ZNF655	AKR1B10	FAM86B1	RB1CC1	DPY19L4	NDRG1	CREB3
PCDHAC2	CPEB4	FAM8A1	FGD2	SLC17A5	RNF217	MTHFD1L	NXPH1	VSTM2A	CASTOR3	AKR1B15	FAM86B2	RGS20	INTS8	MIR30D	HRCT1
PCDHB1	NSG2	KIF13A	PIM1	COX7A2	RNF217-AS1	AKAP12	PHF14	EGFR	AGFG2	BPGM	LOC729732	TCEA1	NDUFAF6	COL22A1	SPAAR
PCDHB5	MSX2	RNF144B	TMEM217	SENP6	TPD52L1	RMND1	THSD7A	PSPH	MUC12	CALD1	MIR3926-2	SOX17	MIR3150A	KCNK9	OR2S2
TAF7	DRD1	MIR548A1	MDGA1	MYO6	HDDC2	ARMT1	ARL4A	LOC650226	TRIM56	AGBL3	FGF20	XKR4	LOC100500773	AGO2	RECK
DIAPH1	CPLX2	ID4	ZFAND3	IRAK1BP1	LINC02523	ESR1	ETV1	ZNF716	SERPINE1	STRA8	MTUS1	SBF1P1	GDF6	PTK2	GLIPR2
GNPDA1	SIMC1	MBOAT1	BTBD9	LCA5	HEY2	IPCEF1	CRPPA-AS1	ZNF733P	COL26A1	STMP1	ATP6V1B2	TMEM68	MTDH	MROH5	GNE
NDFIP1	UNC5A	CDKAL1	KIF6	BCKDHB	NCOA7	TIAM2	CRPPA	LINC01005	CUX1	LUZP6	GFRA2	LINC01606	MATN2	PSCA	MELK
SPRY4	UIMC1	CASC15	UNC5CL	IBTK	HINT3	TFB1M	ANKMY2	ZNF92	PRKRIP1	AKR1D1	HR	LINC00588	RPL30	GML	PAX5
FGF1	PRR7-AS1	HDGFL1	NCR2	UBE3D	MIR5695	ARID1B	TSPAN13	GUSB	ORAI2	KIAA1549	BMP1	CYP7A1	POP1	PLEC	ZBTB5
ARHGAP26	FAM193B	NRSN1	USP49	PGM3	ECHDC1	TMEM242	PRPS1L1	TPST1	RASA4B	ZC3HAV1	PHYHIP	NSMAF	NIPAL2	DGAT1	EXOSC3
ARHGAP26-AS1	HNRNPH1	MRS2	GUCA1B	TBX18	ARHGAP18	SNX9	HDAC9	TMEM248	POLR2J3	KLRG2	PPP3CC	TOX	KCNS2	VPS28	FAM201A
NR3C1	C5orf60	ALDH5A1	TRERF1	NT5E	AKAP7	SYNJ2	ITGB8	MIR4650-2	RASA4	HIPK2	SORBS3	RAB2A	STK3	ZNF251	CNTNAP3
MIR5197	SQSTM1	RIPOR2	UBR2	SNX14	LINC01013	GTF2H5	LOC100506178	STAG3L4	LRRC17	TBXAS1	C8orf58	CHD7	MIR599	DDX11L5	FAM74A1
HMHB1	TBC1D9B	CMAHP	TBCC	SLC35A1	STX7	TULP4	IL6	AUTS2	FBXL13	PARP12	EGR3	ASPH	COX6C	KANK1	SPATA31A3
ADRB2	GFPT2	CARMIL1	BICRAL	AKIRIN2	EYA4	TMEM181	FAM126A	GALNT17	RPL19P12	KDM7A	RHOBTB2	YTHDF3	SPAG1	PUM3	ZNF658
SH3TC2	DUSP22	SCGN	SRF	CNR1	SLC2A12	DYNLT1	KLHL7-DT	MIR3914-1	LHFPL3-AS2	DENND2A	CHMP7	MTFR1	RNF19A	RFX3	GLIDR
ABLIM3	FOXQ1	TRIM38	DNPH1	RNGTT	SGK1	EZR	MALSU1	MIR4650-1	KMT2E-AS1	TMEM178B	R3HCC1	DNAJC5B	ANKRD46	GLIS3-AS1	FGF7P3
AFAP1L1	FOXF2	HIST1H2AC	ABCC10	PNRC1	LINC01010	OSTCP1	IGF2BP3	SBDSP1	PUS7	KIAA1147	NKX2-6	TRIM55	SNX31	GLIS3	ANKRD20A3
CSNK1A1	FOXC1	HIST1H1E	MRPS18A	SRSF12	ALDH8A1	RSPH3	STK31	PMS2P2	ATXN7L1	WEE2-AS1	STC1	CRH	PABPC1	SLC1A1	AQP7P3

LOC642929	SLC35D2	NIBAN2	APOO	ELF4
LINC01189	ZNF367	STXBP1	ZFX	ZNF280C
FAM27C	PRXL2C	ST6GALNAC4	PCYT1B	GPC4
FGF7P6	ZNF782	FAM102A	PPP4R3C	GPC3
SPATA31A5	CTSV	CIZ1	NR0B1	PLAC1
PTGER4P2-CDK2AP2P2	TDRD7	WDR34	SYTL5	MOSPD1
LOC286297	TMOD1	PKN3	BCOR	FHL1
AQP7P1	XPA	PHYHD1	ATP6AP2	ZIC3
FAM27B	TBC1D2	IER5L	LOC100132831	MIR2114
FRG1JP	ANKS6	LINC00963	USP9X	MAMLD1
FRG1HP	COL15A1	PTGES	EFHC2	MTM1
PGM5P2	TGFBR1	FNBP1	FUNDC1	MAGEA4
ANKRD20A4	ALG2	ABL1	DIPK2B	GABRE
LOC100133920	NR4A3	FIBCD1	LOC392452	MAGEA5
FOXD4L5	MSANTD3	FAM78A	MIR222	MAGEA2
FAM122A	CAVIN4	RAPGEF1	KRBOX4	ZNF185
FXN	OR13C2	MED27	ZNF674	ZNF275
BANCR	ABCA1	BARHL1	CHST7	CCNQ
C9orf135	SLC44A1	RALGDS	SLC9A7	BCAP31
MAMDC2	ZNF462	DBH	ZNF630	L1CAM
SMC5	RAD23B	RXRA	SSX3	FLNA
KLF9	KLF4	FCN2	FTSJ1	IKBKG
ZFAND5	ACTL7B	OLFM1	TFE3	GAB3
TMC1	ELP1	QSOX2	SYP	TMLHE
ALDH1A1	MIR32	SEC16A	FAM156A	GYG2P1
ANXA1	TMEM245	MIR4674	PHF8	TTTY3
TRPM6	EPB41L4B	AGPAT2	FOXR2	SPRY3
OSTF1	PALM2	SNHG7	UBQLN2	
PCSK5	AKAP2	TRAF2	SPIN3	
RFK	ECPAS	CACNA1B	ZXDA	
PRUNE2	SHOC1	TUBBP5	SPIN4	
PCA3	MIR4668	ZBED1	MSN	
GNA14	PTBP3	MXRA5	MIR223	
GNAQ	HSDL2	LOC389906	OPHN1	
CEP78	KIAA1958	STS	YIPF6	
TLE1	RGS3	VCX3B	EFNB1	
SPATA31D5P	COL27A1	TBL1X	FAM155B	
SPATA31D1	MIR455	MID1	DLG3	
FRMD3	ATP6V1G1	HCCS	OGT	
GKAP1	TMEM268	MSL3	RPS26P11	
KIF27	TEX48	PRPS2	HDAC8	
RMI1	TNFSF15	FAM9C	CHIC1	
NTRK2	01-Dec	GS1-600G8.3	NEXMIF	
AGTPBP1	ASTN2-AS1	LINC01203	RPS6KA6	
LOC389765	TRIM32	EGFL6	SRPX2	
TUT7	MEGF9	RAB9A	SYTL4	
CTSLP8	GSN	OFD1	NXF3	
SPIN1	GGTA1P	GEMIN8	TCEAL1	
NXNL2	DAB2IP	BMX	ESX1	
MIR4289	TTLL11	PIR	TSC22D3	
UNQ6494	OR5C1	AP1S2	VSIG1	
LOC100129316	RABGAP1	S100G	SNORD96B	
MIR3910-1	LHX2	REPS2	ALG13	
ECM2	NEK6	MIR4768	PLS3	
FBP1	NR6A1	NHS	CUL4B	
MIR2278	OLFML2A	PHKA2-AS1	XIAP	
AOPEP	MAPKAP1	SH3KBP1	STAG2	
ERCC6L2	PBX3	SMPX	OCRL	
LOC158435	ANGPTL2	SMS	APLN	
HSD17B3	GARNL3	SAT1	UTP14A	

Supplementary Table 2

Lists of SMARCA4 regulated genes related to Ca^{2+} and SMARCA4-bound genes in SCCOHT cells. SMARCA4 regulated genes were from ontology terms of ion transmembrane transporter and calcium ion binding in Fig. 2a, b. SMARCA4-bound genes whose loci showed SMARCA4 occupancy within 3 kbp from their transcription start sites were identified from ChIP-seq data in BIN-67 (GSE117734). See also Source Data.

Supplementary Table 3

Primer Name	Sequence (5' to 3')
PTRC_index	ACACTCTTTCCCTACACGACGCTCTTCCGATCTNNNNNNGGCTTTATATATCTTGTGGAAAGGACG
IllSeqR_CR_r	GTGACTGGAGTTCAGACGTGTGCTCTTCCGATCTACTGACGGGCACCGGAGCCAATTCC
P5_Illuseq	AATGATACGGCGACCACCGAGATCTACACTCTTTCCCTACACGACGCTCTTCCGATCT
P7_index_IR_r	CAAGCAGAAGACGGCATACGAGATNNNNNNGTGACTGGAGTTCAGACGTGTGCTCTTCCGATCT
	(NNNNNN = index sequences)
GAPDH_qPCR_For	AAGGTGAAGGTCGGAGTCAA
GAPDH_qPCR _Rev	AATGAAGGGGTCATTGATGG
ITPR3_ qPCR_For	TATGCAGTTTCGGGACCACC
ITPR3_qPCR_Rev	TGCCCTTGTACTCGTCACAC
SMARCA2_ qPCR_For	AGGGGATTGTAGAAGACATCCA
SMARCA2_ qPCR_Rev	TTGGCTGTGTTGATCCATTGG

Supplementary Table 3
Lists of primers used in this study. See also Source Data.
