# Supplementary material for: SMARCA4/2 loss inhibits chemotherapy-induced apoptosis by restricting IP3R3-mediated Ca2+ flux to mitochondria
Source: Nat Commun. 2021 Sep 13;12:5404. doi: 10.1038/s41467-021-25260-9 (PMC8438089; doi:10.1038/s41467-021-25260-9)
Supplement: Supplementary file 3 — Reporting Summary [file 41467_2021_25260_MOESM3_ESM.pdf]

nature research

Corresponding author(s): Julien Prudent and Sidong Huang

Last updated by author(s): Jul 5, 2021

Reporting Summary

Nature Research wishes to improve the reproducibility of the work that we publish. This form provides structure for consistency and transparency in reporting. For further information on Nature Research policies, see our Editorial Policies and the Editorial Policy Checklist.

Statistics

For all statistical analyses, confirm that the following items are present in the figure legend, table legend, main text, or Methods section.

n/a	Confirmed	
☐	☒	The exact sample size (*n*) for each experimental group/condition, given as a discrete number and unit of measurement
☐	☒	A statement on whether measurements were taken from distinct samples or whether the same sample was measured repeatedly
☐	☒	The statistical test(s) used AND whether they are one- or two-sided<br>*Only common tests should be described solely by name; describe more complex techniques in the Methods section.*
☒	☐	A description of all covariates tested
☐	☒	A description of any assumptions or corrections, such as tests of normality and adjustment for multiple comparisons
☐	☒	A full description of the statistical parameters including central tendency (e.g. means) or other basic estimates (e.g. regression coefficient) AND variation (e.g. standard deviation) or associated estimates of uncertainty (e.g. confidence intervals)
☐	☒	For null hypothesis testing, the test statistic (e.g. *F*, *t*, *r*) with confidence intervals, effect sizes, degrees of freedom and *P* value noted<br>*Give P values as exact values whenever suitable.*
☒	☐	For Bayesian analysis, information on the choice of priors and Markov chain Monte Carlo settings
☒	☐	For hierarchical and complex designs, identification of the appropriate level for tests and full reporting of outcomes
☐	☒	Estimates of effect sizes (e.g. Cohen's *d*, Pearson's *r*), indicating how they were calculated

Our web collection on statistics for biologists contains articles on many of the points above.

Software and code

Policy information about availability of computer code

Data collection	1. Annexin V and phase contrast images: IncuCyte® S3 Software (v2016B)<br>2. Western blots and colony formation images: Epson Perfection V700 Photo (Epson)<br>3. RT-qPCR: QuantStudio5 (ThermoFisher)<br>4. IHC images: Aperio Scanscope XT (Leica Biosystems), Lumenera INFINITY X CMOS Camera (Teledyne Lumenera)<br>5. Annexin V flow cytometry: Guava easyCyte HT (Sigma), guavaSoft (2.5)<br>6. Cell viability Assay: InfiniteM200Pro (Tecan)<br>7. Heatmap: pheatmap package in R (4.0.3)<br>8. Cytosolic and mitochondrial Ca2+ contents: ANDOR Spinning Disk dragonfly confocal with Fusion software (ANDOR)
Data analysis	1. CRISPR screen: xcalibr (https://github.com/NKI-GCF/xcalibr), MAGeCK (0.5.8)<br>2. Cytosolic and Mitochondrial Ca2+: Fiji (2.0.0)<br>3. Statistics and graphing: GraphPad Prism 8<br>4. Transcriptome analysis: STAR (2.6.1c), featureCounts (v1.6.4), HTSeq (0.6.1), DESeq2 (1.19.38), clusterProfiler (v3.12.0), RStudio (1.2.1335)<br>5. Annexin V images: IncuCyte® S3 Software (v2016B)<br>6. IHC image analysis: Aperio ImageScope (12.3.3)

For manuscripts utilizing custom algorithms or software that are central to the research but not yet described in published literature, software must be made available to editors and reviewers. We strongly encourage code deposition in a community repository (e.g. GitHub). See the Nature Research guidelines for submitting code & software for further information.

Data

Policy information about availability of data

All manuscripts must include a data availability statement. This statement should provide the following information, where applicable:

- Accession codes, unique identifiers, or web links for publicly available datasets
- A list of figures that have associated raw data
- A description of any restrictions on data availability

Original data for IC50 of chemotherapy drugs are from Genomics of Drug Sensitivity in Cancer (https://www.cancerrxgene.org/). mRNA expression data of SMARCA4/2 and ITPR3 are obtained from the Cancer Cell Line Encyclopedia (https://portals.broadinstitute.org/ccle) for cell lines and downloaded from UCSC Xena (https://xenabrowser.net/datapages/) for TCGA tumors of lung and ovarian cancer patients. Out of 13 SCCOHT patient tumors, RNA-seq data of 10 cases were obtained from a previous study (PMID: 26343384) and that of the other three cases will be deposit before publication. Source data for RNA-seq, microarray, ChIP-seq and ATAC-seq can be found using the accession number GSE120297, GSE117735, GSE121755, GSE109010 and GSE109020. All other data are included in main and supplemental figures and tables.

Field-specific reporting

Please select the one below that is the best fit for your research. If you are not sure, read the appropriate sections before making your selection.

☒ Life sciences ☐ Behavioural & social sciences ☐ Ecological, evolutionary & environmental sciences

For a reference copy of the document with all sections, see nature.com/documents/nr-reporting-summary-flat.pdf

Life sciences study design

All studies must disclose on these points even when the disclosure is negative.

Sample size	Sample sizes for in vitro experiments (at least 3 independent experimental replicates in most experiments unless otherwise indicated) were chosen based on the standard practices of the field. For public data set analyses, sample sizes were determined based on what was available publicaly, authors had no influence over how sample sizes were chosen for the design of these studies. Cell line drug sensitivity data: PMID:23180760, PMID: 27397505. Cell line mRNA: PMID: 22460905. TCGA tumors: https://www.biorxiv.org/content/10.1101/326470v6. Director's Challenge Consortium for the Molecular Classification of Lung Adenocarcinoma (PMID 18641660), KMPlotter (PMID 24367507), and UT Lung SPORE (GSE42127, 23357979)
Data exclusions	No data was excluded from analysis.
Replication	Numbers of replicates for each experiment are indicated in manuscript. All experiments were successfully replicated in at least 2 independent experiments (details of replication are included in figure legends of individual experiments)
Randomization	Mice were randomized into 4 different groups, 5 mice for each group. When tumors reached a volume of approximately 150-200mm3, mice were enrolled for receiving either vehicle or indicated inhibitors. Randomization is not applicable for in vitro experiments as genetic knockouts or wild type cells with different treatments cannot be randomized. Randomization is not applicable for dataset analyses as groups were taken from publicly available data.
Blinding	The person administering the drug or placebo was not blinded to the drug condition due to the complexity of experiments and limited personnel. However, the subsequent measurements were blinded to the treatment information.

Reporting for specific materials, systems and methods

We require information from authors about some types of materials, experimental systems and methods used in many studies. Here, indicate whether each material, system or method listed is relevant to your study. If you are not sure if a list item applies to your research, read the appropriate section before selecting a response.

Materials & experimental systems

n/a	Involved in the study	
☐	☒	Antibodies
☐	☒	Eukaryotic cell lines
☒	☐	Palaeontology and archaeology
☐	☒	Animals and other organisms
☐	☒	Human research participants
☒	☐	Clinical data
☒	☐	Dual use research of concern

Methods

n/a	Involved in the study	
☒	☐	ChIP-seq
☐	☒	Flow cytometry
☒	☐	MRI-based neuroimaging

Antibodies

Antibodies used	Methods, Compounds and antibodies<br>Antibodies against calregulin (Cat# sc-166837), HSP90 (Cat# sc-13119) and β-Actin (Cat# sc-47778) were from Santa Cruz Biotechnology; antibodies against SMARCA2 (Cat# 11996), cleaved PARP(Cat# 5625) and cleaved caspase-3 (Cat# 9664) were from Cell Signaling; Antibody against MICU2 (Cat# ab-101465), VDAC1 (Cat# ab-14734) and GRP75 (Cat# ab-2799) were from Abcam; Antibody against SMARCA4 (Cat# A300-813A) were from Bethyl Laboratories (A300-813A); Antibody against IP3R3 (Cat# 610312) was from BD Pharmingen; Antibody against vinculin (Cat# V4505) was from Sigma-Aldrich; Antibody against MCU (Cat# HPA016480) was from Atlas; Antibody against MICU1 (Cat# orb-323178) was from Biorbyt.<br>Secondary Antibodies: Antibody against Rabbit IgG (Cat# 1706515) from BioRad, Antibody against Mouse IgG (Cat# 1706516) from BioRad.
Validation	calregulin - Santa Cruz sc-166837 validation stated on supplier's website https://www.scbt.com/p/calregulin-antibody-a-9<br>HSP90 - Santa Cruz sc-13119 validation stated on supplier's website https://www.scbt.com/p/hsp-90alpha-beta-antibody-f-8<br>β-Actin - Santa Cruz sc-47778 validation stated on supplier's website https://www.scbt.com/p/beta-actin-antibody-c4<br>SMARCA2 - Cell Signaling # 11996 validation stated on supplier's website https://www.cellsignal.com/products/primary-antibodies/brm-d9e8b-xp-rabbit-mab/11966<br>cleaved PARP - Cell Signaling #5625 validation stated on supplier's website https://www.cellsignal.com/products/primary-antibodies/cleaved-parp-asp214-d64e10-xp-rabbit-mab/5625<br>cleaved caspase-3 - Cell Signaling # 9664 validation stated on supplier's website https://www.cellsignal.com/products/primary-antibodies/cleaved-caspase-3-asp175-5a1e-rabbit-mab/9664<br>MICU2 - abcam ab-101465 validation stated on supplier's website https://www.abcam.com/micu2-antibody-ab101465.html<br>VDAC1 - abcam ab-14734 validation stated on supplier's website https://www.abcam.com/vdac1porin-antibody-20b12af2-ab14734.html<br>GRP75 - abcam ab-2799 validation stated on supplier's website https://www.abcam.com/grp75mot-antibody-jg1-ab2799.html<br>SMARCA4 - bethyl A300-813A validation stated on supplier's website https://www.bethyl.com/product/A300-813A/BRG1+SMARCA4+Antibody<br>IP3R3 - BD pharmingen 610312 validation stated on supplier's website https://www.bdbiosciences.com/en-us/products/reagents/microscopy-imaging-reagents/immunofluorescence-reagents/purified-mouse-anti-ip3r-3.610312<br>vinculin - sigma-aldrich V4505 validation stated on supplier's website https://www.sigmaaldrich.com/CA/en/product/sigma/v4505<br>MCU - Atlas HPA016480 validation stated on supplier's website https://www.atlasantibodies.com/products/antibodies/primary-antibodies/triple-a-polyclonals/mcu-antibody-hpa016480/<br>MICU1 - Biorbyt orb-323178 validation stated on supplier's website https://www.biorbyt.com/micu1-antibody-orb323178.html

Eukaryotic cell lines

Policy information about cell lines

Cell line source(s)	OVCAR4: Dr. E. Wang (University of Calgary, Calgary, originally from NCI); HEC116: Dr. LM. Postovit (Queen's University, Kingston, originally from Japanese Collection of Research Bioresources Cell Bank); BIN-67: Dr. S.R. Goldring (Hospital for Special Surgery, New York, originally derived from patients with ovarian carcinoma treated at the Dana-Farber Cancer Institute (Boston, MA)); SCCOHT-1:Dr. R. Hass (Medical University Hannover, Hannover, generated by Dr. R. Hass); PC9:Dr. R. Bernards (Netherlands Cancer Institute, Amsterdam, originally from Immuno-Biological Laboratories (IBL), Tokyo, Japan); H1703: ATCC, CRL-5889; H1299: ATCC, CRL-5803; H3255:ATCC, CRL-2882; H1437: ATCC, CRL-5872; H1915:ATCC,CRL-5904; HCC827: ATCC, CRL-2868; H838: ATCC, CRL-5844; A549: ATCC, CCL-185; H2030: ATCC,CRL-5914; H1819: ATCC,CRL-5897; H1568: ATCC,CRL-5876; H661: ATCC,HTB-183; H23: ATCC, CRL-5800; A427: ATCC, HTB-53; H522: ATCC, CRL-5810; H2122: ATCC, CRL-5985; H358: ATCC, CRL-5807; H441: ATCC, HTB-174; H1792: ATCC, CRL-5895
Authentication	All cell lines used were authenticated with STR-GenePrinter
Mycoplasma contamination	All cell line were tested and mycoplasma free.
Commonly misidentified lines<br>(See ICLAC register)	No commonly misidentified cell lines were used in the study.

Animals and other organisms

Policy information about studies involving animals; ARRIVE guidelines recommended for reporting animal research

Laboratory animals	8–12-week-old in house bred male NOD.Cg-Prkdcscid Il2rgtm1Wjl/SzJ (NSG) mice.
Wild animals	This study did not involve wild animals.
Field-collected samples	This study did not involve samples collected from the field.
Ethics oversight	Animal experiments were carried-out according to standards outlined in the Canadian Council on Animal Care Standards (CCAC) and the Animals for Research Act, R.S.O. 1990, Chapter c. A.22, and by following internationally recognized guidelines on animal welfare. All animal procedures (Animal Use Protocol) were approved by the Institutional Animal Care Committee according to guidelines of the Canadian Council of Animal Care. All animal experiments were carried-out at the Goodman Cancer Research Center of McGill University.<br>Housing conditions:

April 2020

Temp: 16 degrees min - 24 degrees max
Humidity: 15% low - 60% high
Photoperiod: 7am-7pm light, 7pm-7am dark
60 air exchanges per hour
Top filter: Allentown Polysulfone microbarrier tops with Remay filter paper
Corncob bedding brand: Bulk Tote Corncob Bedding
Supplier: Envigo RMS (Canada) Limited
Food:
Commercial name: Global soy protein-free, irradiated
Brand: Teklad Cat # 2020SX Supplier: Envigo RMS (Canada) Limited
Water:
Reverse Osmosis, Chlorinated
Shredded paper commercial name: Fibercore, Brand: Envirodri (25lbs bag), Supplier: Cedarlane Laboratories Ltd.

Note that full information on the approval of the study protocol must also be provided in the manuscript.

Human research participants

Policy information about studies involving human research participants

Population characteristics

Small cell carcinoma of ovary, hypercalcemic type (SCCOHT) patient tumors - SCCOHT is a very rare diagnosis. It is so rare that there is no specific entry in the IARC book "Cancer Incidence in Five Continents", but this cancer is included in "other" ovarian tumors. The rates for 'other" ovarian tumors are 0.3 per 100,000 women for USA (Whites and Blacks), 0.2 in Canada, 0.3 in China, 0.1 in India, 0.2 in Germany and 0.1 in Australia. Thus there is very little international variance, and almost all cases have a single genetic cause (SMARCA4 pathogenic variants). These cases which are from all over the world (mostly US, Canada, Europe, Australia). The average diagnosis age of the SCCOHT patients in this study is 24.8 years; all patients are women; 15 Stage I, 7 Stage II, 13 stage III, 2 Stage IV, 23 not available (NA); treatment - 19 chemotherapy, 8 chemotherapy + radiation therapy (RT), 1 high dose chemotherapy (HDC), 1 chemotherapy + RT + HDC, 28 NA.
High grade serous ovarian cancer (HGSC) patient tumors- all HGSC patients are women; FIGO stage 1 (13.2%), stage 2 (17.0%), stage 3 (62.3%), stage 4 (7.5%); the average age is 59 years; chemotherapy used in first line of treatment consisted of standard carboplatin and paclitaxel.
Lung adenocarcinoma (LUAD) patient tumors - There were 53 (53.5%) women and 46 men (46.5%). The mean age at time of surgical resection was 66.7 years (±9.6 years). The surgical resections performed included: lobectomy in 81 (81%) patients, 4 (4%) pneumonectomy (4%), 3 (3%) segmentectomy, 11 (11%) wedge resection, and 1 (1%) chest wall resection. The (most common) adenocarcinoma subtypes included not otherwise specified (NOS), mixed subtypes, acinar adenocarcinoma, and mucin-secreting adenocarcinoma. The final pathological stage (coded according to 7th classification) included 67 (67%) Stage I, 16 (16%) Stage II, 16 (16%) Stage III and 1 (1%) Stage IV. 11 patients (11.1%) were treated with adjuvant chemotherapy.

Recruitment

SCCOHT- Because of this low incidence, we reached out to colleagues who had reported cases, or received cases where our opinion had been requested. There is no pre-selection for these cases, which are from all over the world (mostly US, Canada, Europe, Australia). HGSC - All consecutive patients undergoing surgery for gynecologic cancer in the division of gynecologic oncology at the Jewish General Hospital. We do not see any self-selection bias or other biases that might affect the results.
LUAD - The cohort consisted of a group of 100 consecutive patients who underwent surgical resections for lung adenocarcinoma between 2009 and 2011 at the McGill University Health Centre, and in whom lung cancer tissue was collected. No selection biases.

Ethics oversight

Studies on SCCOHT patient tumors were approved by the Institutional Review Board (IRB) at McGill University, McGill IRB # A08-M61-09B. Studies on pathologist-confirmed (B.A.C.) ovarian HGSC samples were approved by the ethics boards at the University Hospitals Network and the Jewish General Hospital respectively - all consecutive patients undergoing surgery for gynecologic cancer in the division of gynecologic oncology at our institution gave informed written consent (IRB protocol #15-070) and tissue samples were kept in the gynecologic oncology tumor bank (IRB protocol #03-041). The studies on of 100 resected LUAD patient tumors was approved by the ethics boards at the McGill University Health Centre (F11HRR, 17212).

Note that full information on the approval of the study protocol must also be provided in the manuscript.

Flow Cytometry

Plots

Confirm that:

☒ The axis labels state the marker and fluorochrome used (e.g. CD4-FITC).

☒ The axis scales are clearly visible. Include numbers along axes only for bottom left plot of group (a 'group' is an analysis of identical markers).

☒ All plots are contour plots with outliers or pseudocolor plots.

☒ A numerical value for number of cells or percentage (with statistics) is provided.

Methodology

Sample preparation

Methods, Annexin-V and Propidium Iodide (PI) Flow Cytometry
OVCAR4: Dr. E. Wang (University of Calgary, Calgary, originally from NCI) and H1703: ATCC, CRL-5889 cells were cultured in Roswell Park Memorial Institute 1640 Medium (Thermo Fisher Scientific, Cat# 11875-093) with 7% fetal bovine serum (Sigma, Cat# F1051), 1% penicillin/streptomycin (Thermo Fisher Scientific, Cat# 15140-122) and 2mM L-glutamine (Thermo Fisher

Scientific, Cat# 25030-081) prior to treatment conditions stated in manuscript.
Cells were harvested after treatment and washed in cold phosphate buffered saline and resuspended in 1X Annexin Binding Buffer (BMS500BB) to 1 million cells/mL prior to addition of fluorescent stains.

Instrument	Guava easyCyte HT (Sigma)
Software	guavaSoft version 2.5
Cell population abundance	Guava easyCyte HT does not support cell sorting functions
Gating strategy	Gating was performed using untreated cells from each experiment. Gating parameters for florescence were determined based on the Annexin V-/Propidium Iodide-, Annexin V+/PI-, Annexin V-/PI+, and Annexin V+/PI+ populations of these untreated cells. The forward and side scatter plots were used to assess cell size and shape and no secondary populations were found in any of our samples.

☒ Tick this box to confirm that a figure exemplifying the gating strategy is provided in the Supplementary Information.
